# Modulation of actin polymerization affects nucleocytoplasmic transport in multiple forms of amyotrophic lateral sclerosis

**DOI:** 10.1038/s41467-019-11837-y

**Published:** 2019-08-23

**Authors:** Anthony Giampetruzzi, Eric W. Danielson, Valentina Gumina, Maryangel Jeon, Sivakumar Boopathy, Robert H. Brown, Antonia Ratti, John E. Landers, Claudia Fallini

**Affiliations:** 1 Department of Neurology, University of Massachusetts Medical School, Worcester, MA 01605 USA; 2 Istituto Auxologico Italiano, IRCCS, Department of Neurology – Stroke Unit and Laboratory of Neuroscience, Milan, Italy; 3 Department of Medical Biotechnology and Translational Medicine, Università degli Studi di Milano, Milan, Italy

**Keywords:** Molecular neuroscience, Amyotrophic lateral sclerosis

## Abstract

Amyotrophic lateral sclerosis (ALS) is a fatal neurodegenerative disease of unknown etiology. Although defects in nucleocytoplasmic transport (NCT) may be central to the pathogenesis of ALS and other neurodegenerative diseases, the molecular mechanisms modulating the nuclear pore function are still largely unknown. Here we show that genetic and pharmacological modulation of actin polymerization disrupts nuclear pore integrity, nuclear import, and downstream pathways such as mRNA post-transcriptional regulation. Importantly, we demonstrate that modulation of actin homeostasis can rescue nuclear pore instability and dysfunction caused by mutant PFN1 as well as by *C9ORF72* repeat expansion, the most common mutation in ALS patients. Collectively, our data link NCT defects to ALS-associated cellular pathology and propose the regulation of actin homeostasis as a novel therapeutic strategy for ALS and other neurodegenerative diseases.

## Abstract (web summary)

Although defects in nucleocytoplasmic transport (NCT) may be central to the pathogenesis of ALS, the molecular mechanisms modulating the nuclear pore function are still largely unknown. Here, authors show that genetic and pharmacological modulation of actin polymerization disrupts nuclear pore integrity and can be targeted to rescue nuclear pore instability and dysfunction caused by mutant PFN1 as well as by C9ORF72 repeat expansion

## Introduction

Amyotrophic lateral sclerosis (ALS) is a neurodegenerative disease of unknown etiology characterized by progressive loss of motor neurons (MNs). Most ALS cases are sporadic and ~10% are familial, yet the two classes are clinically indistinguishable suggesting that similar pathways may be responsible for the MN degeneration. Defects in nucleocytoplasmic transport (NCT) have been observed in both cellular and in vivo models of ALS and reinforced by pathological evidence in familial and sporadic ALS patients^1–7^. Nuclear deficiency of RNA-binding proteins (RBPs) such as TDP-43 and FUS is a pathological hallmark of the disease^8^, strongly supporting a link between NCT and ALS pathogenesis.

NCT is a tightly regulated process that actively controls the separation and exchange between cytoplasmic and nuclear proteins and RNAs. It is centered on the function of the nuclear pore complex (NPC), a multiprotein complex spanning the whole nuclear envelope and comprised of about 30 different nucleoporins^9^. Other key players controlling NCT are the small GTPase Ran, its GTPase-activating protein RanGAP1, and the carrier proteins importins and exportins. The cellular distribution of these factors confers directionality to the transport^10^, while the structural integrity and density of the NPCs across the nuclear envelope modulate the efficiency of the NCT. Interestingly, some nucleoporins are the longest-lived proteins in the cell, and they are never or very slowly replaced once the NPC is formed in post-mitotic neurons^11,12^.

Toxic insults such as oxidative stress and protein aggregation have been shown to negatively impact NCT^13,14^. Many mutant ALS-linked proteins show an increased tendency to aggregate, including SOD1, TDP-43, FUS, and Profilin1 (PFN1). PFN1 is a small actin-binding protein that positively regulates actin polymerization in a formin-dependent manner^15^. Several mutations in PFN1 have been identified in ALS patients (C71G, M114T, G118V, A20T, T109M, Q139L, E117G), representing less than 1% of all familial cases^16,17^. Nonetheless, mutations in PFN1 and other cytoskeletal-related proteins such as TUBA4A and KIF5A suggest that defects to the cytoskeletal structure and/or function may play an important role in triggering neurodegeneration^18–20^. ALS-linked mutations in PFN1 render the protein unstable and aggregation prone, leading to the formation of cytoplasmic inclusions^16,21^. The ability of mutant PFN1 to associate with -actin is also impaired, and mutant PFN1 MNs show morphological abnormalities, such as smaller growth cones and shorter axons^16^. The C71G and G118V mutations have consistently shown more severe phenotypes compared to other mutations in in vitro assays, and transgenic animals expressing these mutations develop ALS-like disease and die prematurely^22,23^. However, the molecular mechanisms leading to MN degeneration due to mutations in PFN1 are still unknown. Here, we demonstrate that alterations to the actin cytoskeleton caused by mutant PFN1 disrupt the NCT and consequently the normal function of ALS-relevant RBPs, leading to MN dysfunction. Interestingly, modulating actin homeostasis was able to rescue the NCT defects caused by not only mutant PFN1 but also *C9ORF72* repeat expansion, suggesting this pathway could represent a novel therapeutic strategy for ALS.

## Results

### Mutations in PFN1 impair nucleocytoplasmic transport

**Fig. 1:**
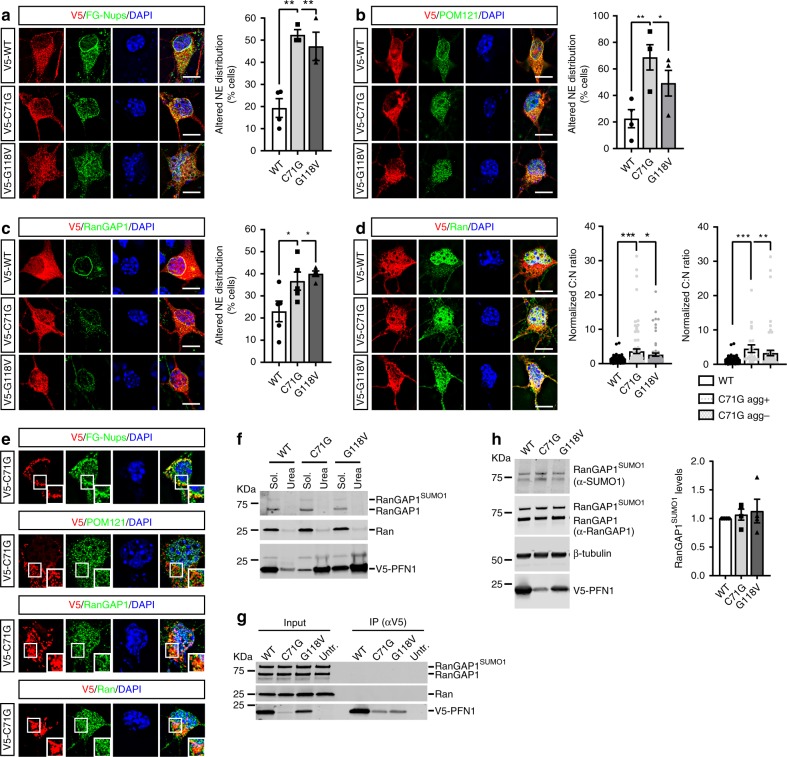
Mutant PFN1 alters the composition and density of NPCs. **a**, **c** Antibody against FG-Nups (**a**, green; mAb414), POM121 (**b**, green), and RanGAP1 (**c**, green) localization to the NE (identified based on DAPI staining) is altered in a higher percentage of MNs expressing V5-tagged mutant PFN1 vs PFN1^WT^ control (red). **d** Ran (green) cytoplasm to nucleus (C:N) ratio is increased in MNs expressing V5-WT or mutant PFN1 (red), regardless of the presence of aggregates (agg), indicating possible functional defects in the segregation of cytoplasmic and nuclear proteins. **e** PFN1^C71G^ -positive cytoplasmic inclusions (red) as described in Wu et al. (2011) in MNs are not positive for FG-Nups, POM121, RanGAP1, or Ran (green), suggesting no co-aggregation under these conditions. **f** No difference in the solubility of Ran (middle panel) or RanGAP1 (top panel) caused by the expression of PFN1 mutants when assayed in HEK293 cells using detergent-based cellular fractionation. Triton X-100 (2%) and urea (8M) were used to extract the soluble and insoluble fraction, respectively. **g** Representative blot of a co-immunoprecipitation (co-IP) assay between V5-tagged WT or mutant PFN1 and RanGAP1 (top panel) or Ran (middle panel). No bands were detected in the IP pellet, suggesting lack of interaction. **h** Representative western blot and quantification showing unchanged levels of SUMO1-modified RanGAP1 in the presence of mutant PFN1. Both antibodies against SUMO1 (top panel) and RanGAP1 (second panel) detect a band ~80 KDa corresponding to SUMOylated RanGAP1. V5 antibody (bottom panel) shows the expression of the V5-tagged PFN1 protein, while β-tubulin was used as a loading control. Bars are mean ± SEM; *p < 0.05, **p < 0.01, ***p < 0.001. *N* = 3–5 independent experiments (**a**–**c**), 76–103 cells from 6 independent experiments (**d**), 4 independent experiments (**h**). DAPI (blue) was used to detect the nucleus and assess cell health. Scale bars: 10 µm. See also Supplementary Figs. 1–5, and Supplementary Table 1 for details on statistics

To investigate whether mutant PFN1 toxicity is associated with nucleocytoplasmic transport (NCT) defects, we examined its effects on the distribution of essential factors controlling this process. Wild type (WT) or mutant (i.e., C71G and G118V) V5-tagged PFN1 were transfected in primary motor neurons (MNs) for 4 days. Similar cellular distribution and expression was observed for all constructs. No effect on cell survival was evident at this time point due to the expression of mutant PFN1 (Supplementary Fig. 1). To visualize the localization and composition of the nuclear pore complex (NPC) along the nuclear envelope (NE), we stained MNs expressing WT or mutant PFN1 with antibodies recognizing (1) nucleoporins of the FG-Nup family (i.e., Nup62, Nup153, Nup214, and Nup358; mAb414^24^), (2) Nup358/RanBP2, and (3) the transmembrane Nup POM121, given their essential role in regulating NPC structure and function^25–27^. In PFN1^WT^ cells, all nucleoporins examined displayed a strong, punctate staining around the nucleus, as identified by DAPI staining, similar to mock-transfected controls (Supplementary Fig. 2). In contrast, a significantly higher percentage of mutant PFN1 MNs showed reduced or absent staining at the NE (Fig. 1a, b, Supplementary Fig. 3). Consistent with its known association to the NPC via RanBP2, RanGAP1 localized along the NE in both mock-transfected controls and PFN1^WT^ cells, while its staining pattern was partially or completely disrupted in mutant PFN1 MNs (Fig. 1c, Supplementary Fig. 2). The presence of mutant PFN1 led the transport factor Ran to be abnormally redistributed to the cytoplasm, in contrast to its mostly nuclear localization in PFN1^WT^ cells (Fig. 1d, Supplementary Fig. 2). This effect was more pronounced in cells containing visible inclusions, although MNs with no obvious aggregates still had Ran cytoplasm:nucleus (C:N) ratios significantly higher than PFN1^WT^ values. No co-aggregation of any of the tested proteins with PFN1^C71G^-positive inclusions was observed by immunofluorescence, detected by V5-staining (Fig. 1e), solubility assay (Fig. 1f), or co-immunoprecipitation (Fig. 1g). In addition, no changes in RanGAP1 SUMOylation, which is necessary for its association with the NPC^28^, were detected (Fig. 1h). Similarly, no difference in the overall levels of the tested nucleoporins was observed in all conditions, while a slight reduction in Ran levels was present in PFN1^C71G^ MNs (Supplementary Fig. 4). We did not observe changes to the localization of karyopherins Exportin 1 (XPO1) and Importin-β, though a small reduction in XPO1 levels was detected in PFN1^C71G^ MNs (Supplementary Fig. 5). In all, these data suggest that in the presence of mutant PFN1, NPCs are either reduced in number or structurally compromised because of the lack of essential nucleoporins, and additional key players in NCT are abnormally distributed. Future studies will be required to directly observe and characterize such structural defects.

### Mutant PFN1 alters the structure of the nuclear membrane

**Fig. 2:**
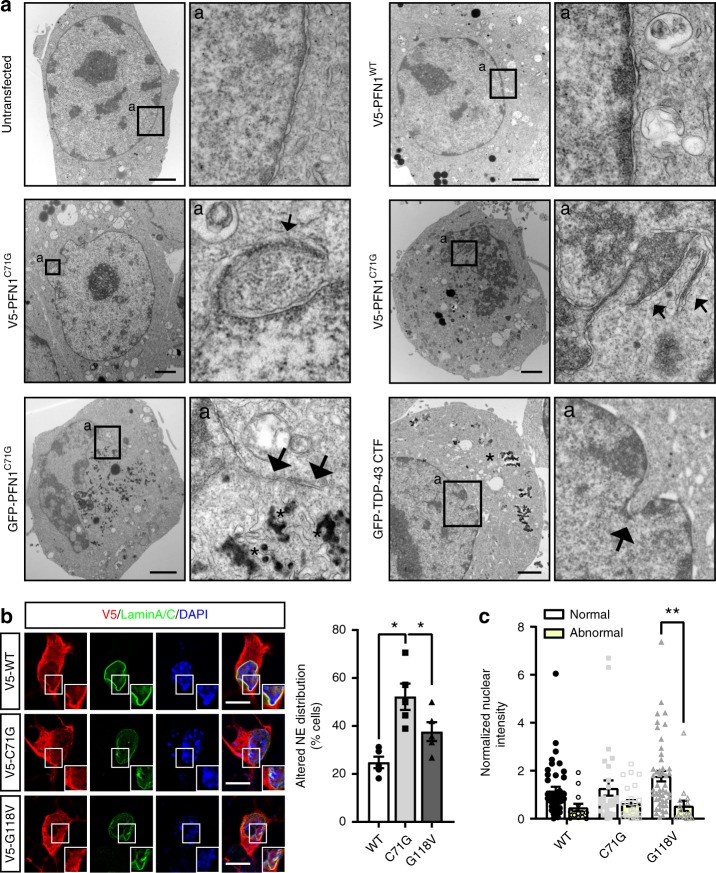
The nuclear membrane integrity is compromised by mutant PFN1. **a** Transmission electron microscopy shows the presence of protrusions and folds (arrows) in Neuro2a cells expressing V5- or GFP-tagged PFN1^C71G^ compared to untransfected (Untr.) or PFN1^WT^ cells, similar to what observed in the presence of TDP-43 C-terminal fragment (CTF). Arrows point to anomalous membrane structures in the nucleus. Aggregates (asterisks) are visible as dark amorphous structures in the cytoplasm. **b** Lamin A/C (green) distribution at the NE is altered in a higher percentage of mutant PFN1 MNs. **c** Overall nuclear levels of Lamin A/C also appear slightly reduced in cells with abnormal staining. Boxes indicate areas enlarged in insets. DAPI (blue) was used to detect the nucleus and assess cell health. Scale bars: 2 µm in (**a**), 10 µm in (**b**). Bars are mean ± SEM; *p < 0.05, **p < 0.01. *N* = 5 independent experiments (**b**) and 14–47 cells from 5 independent experiments (**c**). See also Supplementary Fig. 2 and Supplementary Table 1 for details on statistics

Since the NPC is tightly connected with the nucleoskeleton, we further investigated the effect of mutant PFN1 on the nuclear structure using transmitted electron microscopy. Similarly to what was observed in cells expressing TDP-43 C-terminal fragment^1^, we found that the expression of either V5-tagged or GFP-tagged PFN1^C71G^ in Neuro2a cells led to severe defects in the structure of the nucleus, with the presence of frequent folds, invaginations, and protrusions that were never observed in untransfected or PFN1^WT^-transfected N2a cells (Fig. 2a). This observation was confirmed by immunofluorescence analysis of Lamin A/C, one of several proteins constituting the nuclear lamina such as Lamin B1 and B2, emerin, Lamin B receptor, Nurim, MAN1, LAP1A-C, and LAP2^29^. This analysis showed non-uniform and irregular staining in MNs expressing mutant PFN1 (Fig. 2b). Of note, we noticed that expression of PFN1^WT^ itself caused a slight increase in the percentage of cells with abnormal lamin staining compared to mock-transfected cells (Supplementary Fig. 2). We also found that cells with abnormal Lamin A/C staining were characterized by reduced nuclear levels of the protein (Fig. 2c) but no increase in cytoplasmic levels (Supplementary Fig. S4), suggesting that Lamin A/C may be targeted for degradation as a consequence of its altered localization.

### NPC integrity is compromised in patient-derived lymphoblasts

**Fig. 3:**
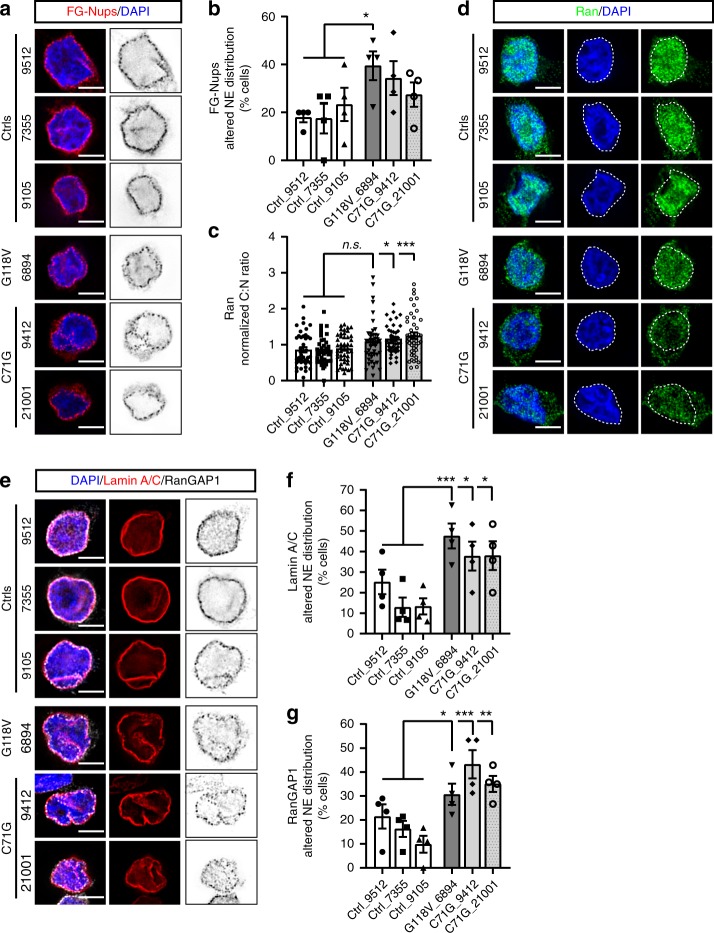
NPC composition is altered in patient-derived lymphoblast cells. Immunofluorescence analyses and quantification of the localization of FG-Nups (**a**, **b**; red), Ran C:N ratio (**c***,* **d**; green), Lamin A/C (**e***,* **f**; red) and RanGAP1 (**e**, **g**; black) reveal alterations to the NPC composition in lymphoblast cells derived from 3 ALS patients carrying PFN1 C71G or G118V mutation compared to 3 control lines. Quantifications show an increase in the percentage of cells with disrupted Lamin A/C and RanGAP1 staining for all lines, while FG-Nups and Ran were significantly altered only in the G118V or C71G mutant lines, respectively. Bars are mean ± SEM. *p < 0.05, **p < 0.01, ***p < 0.001, n.s. non-significant. *N* = 4 independent experiments (**b**, **f**, **g**); 45–48 cells from 3 independent experiments (**c**). DAPI (*blue*) was used to dete*c*t the nucleus and assess cell health. Scale bars: 10 µm. See also Supplementary Fig. 6 and Supplementary Table 1 for details on statistics

To verify that the effect of mutant PFN1 on the nuclear pore occurs also in human ALS patient cells, we performed immunofluorescence assays in immortalized lymphoblast cells derived from 3 controls and 3 patients carrying either the C71G or G118V *PFN1* mutation^16^. Analyses of the localization and staining pattern of FG-Nups, RanGAP1, Ran, and Lamin A/C were performed on all lymphoblast lines. We found that the percentage of cells with abnormal staining of Lamin A/C and RanGAP1 was significantly increased in all three lines endogenously expressing mutant PFN1 (Fig. 3), while FG-Nups—recognized by the mAb414 antibody (AbCam)—showed a significant change only in the G118V line. Further, we found that the nucleocytoplasmic localization of Ran was significantly altered in the C71G lines. No changes in the overall levels of all proteins tested, including endogenous PFN1, were detected (Supplementary Fig. 6). To test whether the changes in Ran localization were caused by alteration to the integrity of the nuclear membrane, we assessed the ability of a large inert molecule (i.e., 70 KDa-Dextran) to bypass the NPC and accumulate in the nucleus. No increase in the presence of leaky nuclei was observed suggesting that disruption to nuclear membrane integrity is not an early event caused by mutant PFN1, at least under these experimental conditions (Supplementary Fig. 6). Together, these data support the hypothesis that endogenous levels of mutant PFN1 directly affects the nuclear pore structure/stability in ALS patient cells, possibly leading to neuronal degeneration.

### Nuclear import is greatly reduced by mutant PFN1

**Fig. 4:**
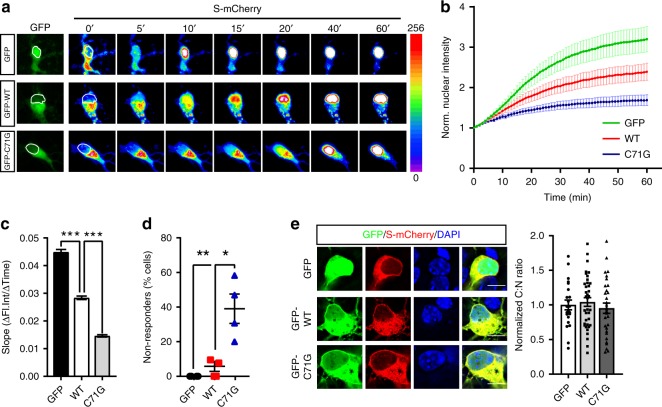
Mutant PFN1 alters the efficiency of nuclear import. **a**, **b** Time-lapse images and quantification of S-mCherry nuclear import dynamics in cortical neurons expressing GFP or GFP-tagged PFN1 (green) and treated with LMB. S-mCherry levels are shown as 16-bit heat map. Dashed lines indicate the nucleus. **c**, **d** Regression analysis of S-mCherry nuclear import kinetics (**c**) and percentage of cells whose nuclear levels did not rise above 1.2 folds over initial values upon the application of LMB (i.e., non-responders, **d**). **e** S-mCherry (red) C:N ratio is not affected by mutant PFN1 (green) in untreated cells. GFP alone, used as control, localizes to both nucleus and cytoplasm. DAPI (blue) was used to detect the nucleus and assess cell health. Scale bars: 10 µm. Bars are mean ± SEM; *p < 0.05, **p < 0.01, ***p < 0.001. *N* = 44 cells from 4 independent experiments (**b**, **c**), 4 independent experiments (**d**), 22–37 cells from 3 independent experiments (**e**). See Supplementary Table 1 for details on statistics

To further explore the functional consequences of the structural defects caused by mutant PFN1 on the NCT, we measured the rate of nuclear import by live cell imaging using a NLS-NES-mCherry (Shuttling (S)-mCherry) reporter^30^. Cortical neurons were co-transfected with GFP or GFP-tagged PFN1 and S-mCherry. S-mCherry localized mainly to the cytoplasm in all conditions due to the stronger effect of the nuclear export signal (NES) compared to the nuclear localization signal (NLS) (Fig. 4e). Thirty-six hours after transfection, cells were treated with Leptomycin B (LMB)—a selective inhibitor of Exportin 1—to inhibit nuclear export, leading to a measurable time-dependent accumulation of the reporter in the nucleus. We found that the expression of mutant PFN1 led to a significant reduction in import rates compared to both GFP- and PFN1^WT^-transfected cells (Fig. 4a–c), and an increase in the percentage of non-responder cells (39% in C71G vs 6% WT) (Fig. 4d). Expression of PFN1^WT^ also reduced import rates compared to the GFP control, but to a lesser degree compared to mutant PFN1. In all, these data suggest that mutant PFN1’s ability to affect nuclear stability, by altering the composition and/or the number of functional NPCs, leads to severe nuclear import defects.

### Mutant PFN1 alters mRNA post-transcriptional regulation

**Fig. 5:**
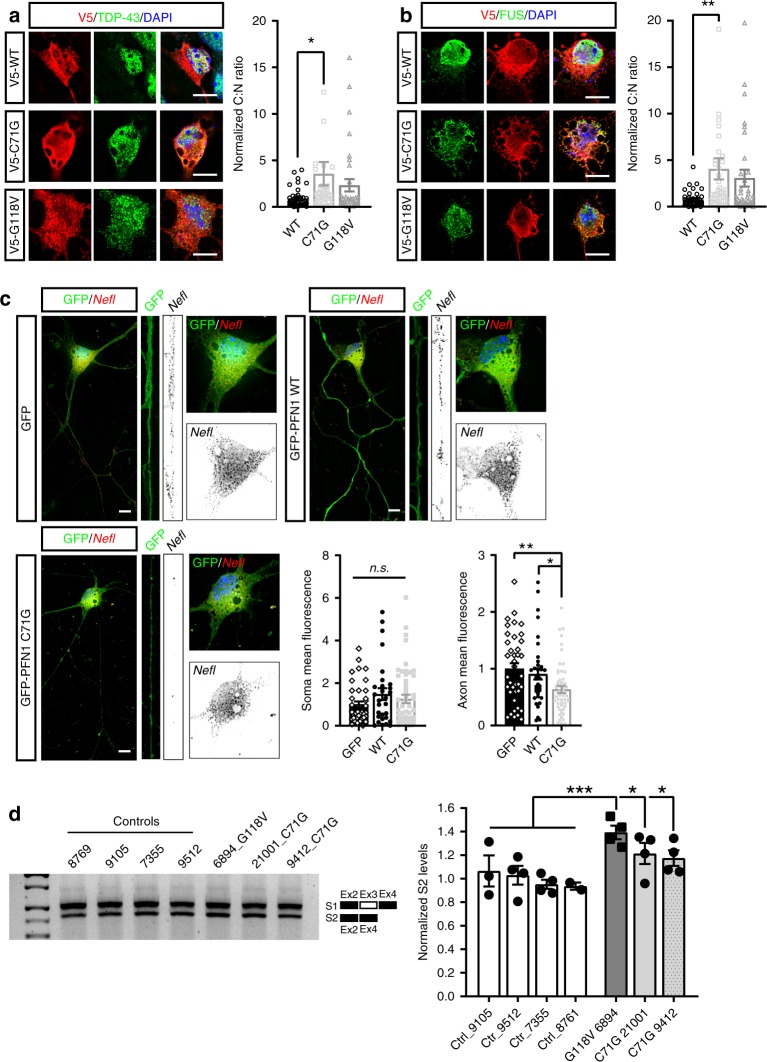
Mutant PFN1 perturbs RBPs cellular distribution and function. **a**, **b** Mutant PFN1 (red) causes redistribution of nuclear RBPs TDP-43 (**a**, green) and FUS (**b**, green) to the cytoplasm, as quantified by their cytoplasm to nucleus (C:N) ratios. **c** RNA-FISH analysis reveals that the *Nefl* mRNA (*black*) levels in the axon, but not in the cell soma, are significantly reduced due to the expression of GFP-PFN1^C71G^ (green) compared to GFP or GFP-PFN1^WT^-expressing MNs. **d** Representative DNA gel and quantification of the levels of the *POLDIP3* S2 variant over total *POLDIP3* levels (S1 + S2) in controls versus mutant PFN1 lymphoblast lines. DAPI (blue) was used to detect the nucleus and assess cell health. Scale bars: 10 µm. Bars are mean ± SEM; *p < 0.05, **p < 0.01, ***p < 0.001. *N* = 34–47 cells from 3–4 independent experiments (**a***,* **b**), 29–53 cells from 4 independent experiments (**c**), 4 independent experiments (**d**). See also Supplementary Figs. 5–10, and Supplementary Table 1 for details on statistics

One of the main classes of proteins that shuttle between the nucleus and the cytoplasm are RBPs which control the post-transcriptional fate of mRNAs. Thus, we investigated the impact of mutant PFN1-dependent disturbance to NCT on the distribution of RBPs by quantifying the nuclear and cytoplasmic levels of the mostly nuclear proteins TDP-43 and FUS, and of the mostly cytoplasmic proteins SMN and FMRP. As expected, the majority of TDP-43 and FUS was nuclear in WT-expressing MNs, while mutant PFN1 expression led to a shift in the C:N ratio of both proteins (Fig. 5a, b). Again, no effect of PFN1^WT^ expression was observed on the localization of the proteins compared to untransfected controls (Supplementary Fig. 7). On the contrary, there was no quantifiable changes in the distribution of the mostly cytoplasmic FMRP and SMN proteins, although we observed fewer SMN-positive nuclear gems (Supplementary Fig. 8). We also observed a significant reduction in TDP-43 localization to the proximal motor axon (Supplementary Fig. 7) and an increase in TDP-43 aggregation, possibly mediated by TDP-43’s illegitimate interaction with mutant PFN1 (Supplementary Fig. 9). Together, these data suggest that mutant PFN1 perturbs the distribution of ALS-relevant nuclear RBPs by destabilizing the NPC and impairing nuclear import. To investigate the downstream consequences of PFN1-mediated mislocalization of such RBPs, we focused as a proof of principle on TDP-43’s well-studied regulatory activity on the axonal localization of the *neurofilament L* (*Nefl*) mRNA^31^ and on the splicing of *POLDIP3* pre-mRNA^32^. Quantitative fluorescence in situ hybridization was performed in MNs expressing either GFP or GFP-tagged WT or C71G PFN1 (Fig. 5c, Supplementary Fig. 10). While no difference in the somatic levels of the *Nefl* mRNA was detected, its axonal levels were severely reduced in C71G-expressing MNs. Immortalized lymphoblast cell lines derived from 4 controls and 3 patients carrying *PFN1* mutations^16^ were used to evaluate the abundance of two alternatively spliced *POLDIP3* variants—S1 and S2—by RT-PCR (Fig. 5d). In all ALS lines, the relative levels of isoform S2 were significantly increased over control cells. Together these data suggest a loss of function for TDP-43 in mutant PFN1 cells, possibly due to its nucleocytoplasmic redistribution.

### Nuclear export inhibitor improves ALS relevant phenotypes

**Fig. 6:**
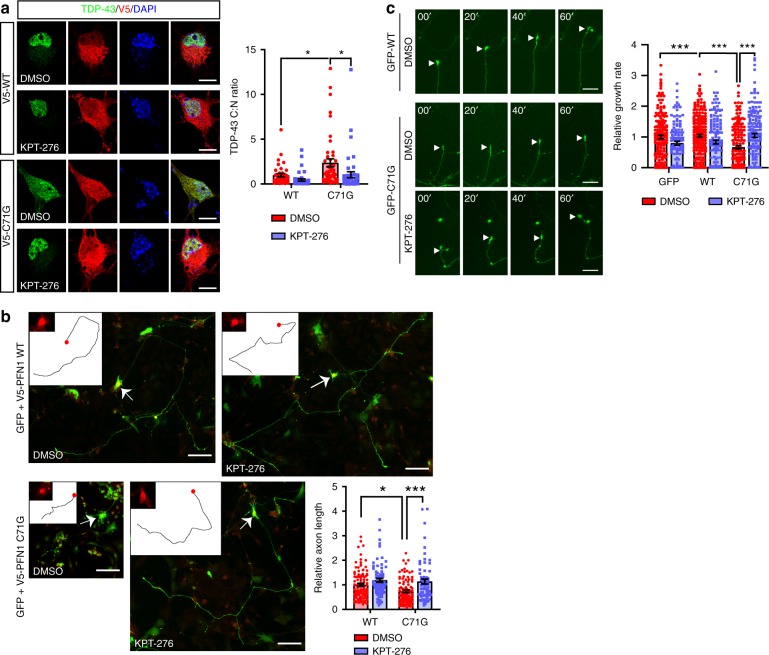
Inhibition of nuclear export rescues PFN1-dependent cellular defects. **a** KPT-276 treatment rescues TDP-43 (green) C:N ratio in MNs expressing mutant PFN1 (red). **b** MNs expressing PFN1 C71G had significantly shorter axons compared to WT-expressing cells. This defect was fully rescued by KPT-276 treatment. Insets show the tracing of the primary axon and the expression of V5-PFN1 in the cell body (red, arrows). **c** Representative time lapse images and quantification of axon outgrowth in the presence or absence of KPT-276. A full rescue of the outgrowth defects was observed following KPT-276 treatment. DAPI (blue) was used to detect the nucleus and assess cell health. DMSO was used as vehicle control. Scale bars: 10 µm. Bars are mean ± SEM; *p < 0.05, ***p < 0.001. *N* = 40–46 cells from 4 independent experiments (**a**), 67–107 cells from 4 independent experiments (**b**), 116–207 cells from 4 independent experiments (**c**). See also Supplementary Table 1 for details on statistics

To assess the causality between NCT disturbance, RBP mislocalization, and MN pathology, we investigated the potential of nuclear export inhibitor KPT-276, a well-established selective inhibitor of XPO1^6,33,34^, to rescue PFN1-dependent defects. First, PFN1^WT^ or PFN1^C71G^ expressing MNs were treated with 50 nM KPT-276 or DMSO for 6 h prior to fixation and the C:N ratio of TDP-43 was determined. KPT-276 treatment was able to fully rescue TDP-43 cytoplasmic mislocalization (Fig. 6a), confirming successful inhibition of nuclear export. Next, we sought to determine the potential of KPT-276 treatment to rescue axonal outgrowth defects previously described in mutant PFN1 MNs^16^. MNs expressing mutant PFN1 and treated with vehicle alone had significantly shorter axons compared to PFN1^WT^ cells, while KPT-276 treatment fully rescued the defect (Fig. 6b). Similar rescue was observed by measuring the rate of axon growth by live cell imaging over the course of 1 h (Fig. 6c, Supplementary Movie 1). Together, these data support a direct link between NCT, mRNA regulation, and PFN1-dependent ALS cellular defects.

### Modulation of actin polymerization modifies NCT

**Fig. 7:**
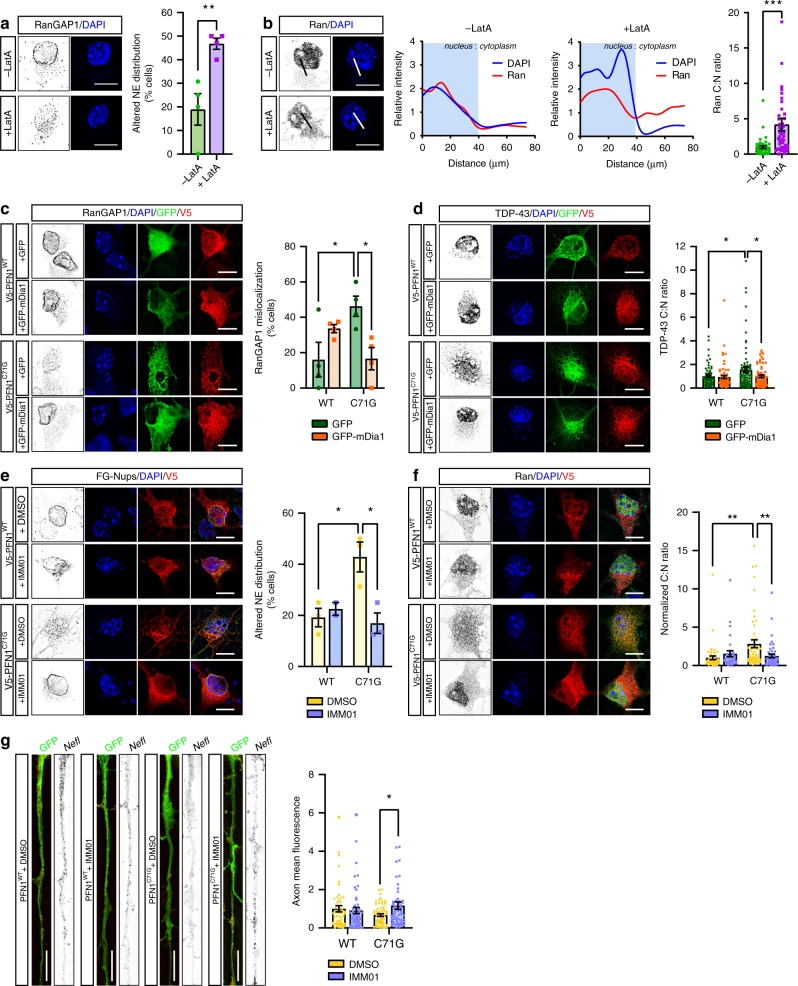
Actin homeostasis is a significant modulator of NPC structure and function. **a**, **b** RanGAP1 (**a**) and Ran (**b**) localization is disrupted by Latrunculin A (LatA) treatment. Line plots of Ran and DAPI intensity are shown. **c**, **d** Representative images and quantification show rescue of RanGAP1 (**c**, black) and TDP-43 (**d**, black) mislocalization due to the overexpression of GFP-mDia1 (green) in MNs expressing PFN1^C71G^ (red). **e**–**g** Representative images and quantification show rescue of FG-Nups (**e**, black), Ran (**f**, black), and *Nefl* mRNA (**g**, black) mislocalization following treatment with IMM01 compared to DMSO alone in MNs expressing PFN1^C71G^. Scale bars: 10 µm. Bars are mean ± SEM. *p < 0.05, **p < 0.01, ***p < 0.001. *N* = 4 independent experiments (**a***,* **c**), 45–50 cells from 4 independent experiments (**b**), 61–76 cells from 5 independent experiments (**d**), 3 independent experiments (**e**), 31–52 cells from 3 independent experiments (**f**), 53–60 cells from 4 independent experiments (**g**). See also Supplementary Figs. 11 and 12 and Supplementary Table 1 for details on statistics

PFN1’s main cellular function is to promote actin polymerization by facilitating formin-based actin nucleation and elongation, a function impaired by the presence of ALS-linked mutations^16,21^. Thus, we hypothesized that PFN1-mediated disruption of the actin network could directly interfere with protein nuclear shuttling by affecting nuclear stability. Supporting this hypothesis, we observed a severe mislocalization of RanGAP1, FG-Nups, and Ran in MNs treated with the actin depolymerizing drug Latrunculin A (LatA) (Fig. 7a, b and Supplementary Fig. 11), resembling the phenotypes identified in mutant PFN1 MNs. LatA-treated MNs also had smaller nuclei with condensed DNA, suggesting the disruption of the actin cytoskeleton interfered with normal nuclear morphology. No increased cell death or apoptosis was observed under these treatment conditions (Supplementary Fig. 11). To further this observation, we used a complementary approach and attempted to rescue NPC defects in PFN1^C71G^ MNs by positively modulating actin polymerization. To this end, we took advantage of the ability of formins to promote actin polymerization, albeit at a slower rate, even in the absence of functional PFN1^35^. Overexpression of a constitutively active form of the formin mDia1 (FH1-FH2 domains), here on called mDia1, in mutant PFN1 MNs was indeed able to restore normal actin homeostasis (Supplementary Fig. 12), without changing the aggregation propensity of PFN1^C71G^. Importantly, while mutant PFN1 MNs expressing GFP alone had significantly higher frequency of disrupted RanGAP1 staining, as previously observed (see Fig. 1), the expression of GFP-mDia1 fully rescued the defect (Fig. 7c). Similarly, GFP-mDia1 expression was able to rescue TDP-43 mislocalization (Fig. 7d) in mutant PFN1 MNs. As a parallel approach, we induced actin polymerization using the small molecule Intramimic-01 (IMM01). This is a specific activator of formin function that acts by binding to formin and preventing its autoinhibition^36^. Treatment of primary MNs with low concentrations of IMM01 (i.e., 0.1 µM) for 24 h led to the rescue of FG-Nups defective localization at the nuclear envelope, as well as of the Ran gradient (Fig. 7e, f). No induction of apoptosis was detected under these conditions (Supplementary Fig. 11). Importantly, we found that cytoskeletal remodeling induced by IMM01 treatment was also able to rescue the defects in the axonal localization for the *Nefl* mRNA (Fig. 7g).

**Fig. 8:**
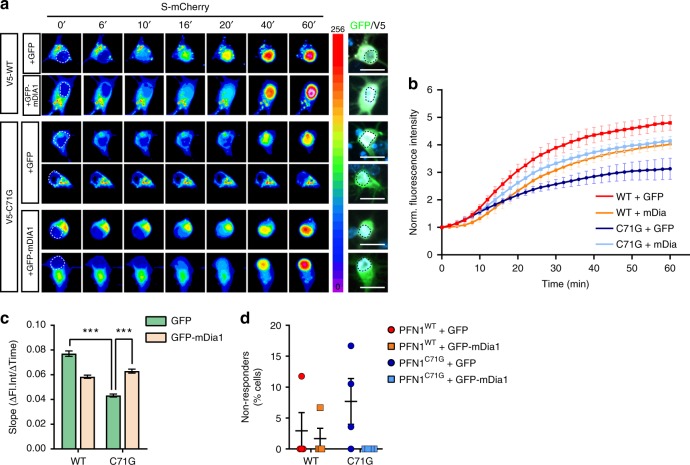
Formin mDia1 overexpression rescues nuclear import defects. **a**, **b** Time-lapse images and quantification of S-mCherry nuclear import dynamics upon treatment with LMB in cortical neurons expressing GFP or GFP-mDia1 (green) and V5-tagged PFN1 (white). S-mCherry levels are shown as 16-bit heat map. Dashed lines indicate the nucleus. Cells were post-fixed and stained to verify the expression of V5-PFN1 constructs (white). **c**, **d** Regression analysis of S-mCherry nuclear import kinetics (**c**) and percentage of non-responder cells (**d**). DAPI (blue) was used to detect the nucleus and assess cell health. Scale bars: 10 µm. Bars are mean ± SEM. ***p < 0.001. *N* = 43–50 cells from 4 independent experiments. See also Supplementary Table 1 for details on statistics

To assess whether actin modulation could also rescue the function of the nuclear pore, we again measured import dynamics of S-mCherry in the presence of mDia1 overexpression. A significant improvement of the import dynamics was observed in PFN1^C71G^ neurons following mDia1 expression (Fig. 8), suggesting that changes in actin homeostasis can influence the stability of the NPC and/or NE, affecting protein shuttling. Together, these data support the existence of a direct link between actin polymerization, NCT, and mRNA post-transcriptional regulation, and suggest that these pathways could be central to the onset and/or progression of the degenerative process in PFN1-linked ALS.

### Modulation of actin rescues NCT defects in C9ORF72-ALS

**Fig. 9:**
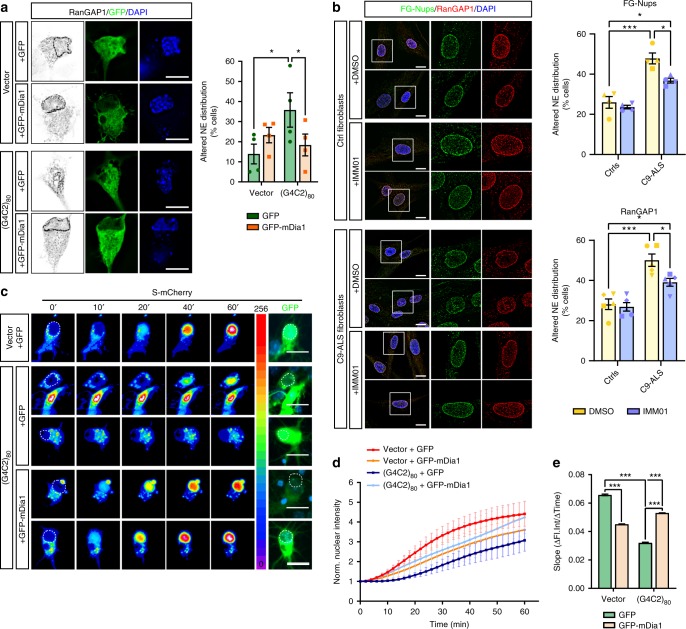
Actin modulates NPC function in C9ORF72-ALS. **a** Significant rescue of RanGAP1 (black) mislocalization to the NE in MNs expressing the *C9ORF72* repeat expansion (G4C2)_80_ was achieved due to the overexpression of GFP-mDia1 compared to GFP alone (green). **b** Fibroblasts derived from 3 ALS patients carrying *C9ORF72* repeat expansions show increased mislocalization of both RanGAP1 and FG-Nups compared to 3 healthy controls. Treatment of these cells with IMM01 significantly rescued the defects. DMSO was used as vehicle control. **c**–**e** Time-lapse images (**c**), quantification (**d**) and regression analysis (**e**) of S-mCherry nuclear import dynamics in cortical neurons expressing GFP or GFP-mDia1 (green) and (G4C2)_80_. S-mCherry levels are shown as 16-bit heat map. Dashed lines indicate the nucleus. DAPI (blue) was used to detect the nucleus and assess cell health. Scale bars: 10 µm. Bars are mean ± SEM. *p < 0.05, ***p < 0.001. *N* = 4 (**a**) and 5 (**b***,* **e**) independent experiments, and 51–64 cells from 5 independent experiments (**c***,* **d**). See also Supplementary Figs. 13 and 14 and Supplementary Table 1 for details on statistics

Next, we sought to investigate whether modulating the actin cytoskeleton could be beneficial in other forms of ALS characterized by defects to the nuclear pore function, such as those caused by a repeat expansion in *C9ORF72* gene. Thus, we transfected MNs with a synthetic construct expressing 80 GGGGCC repeats (G4C2)_80_, that had been previously shown to induce the presence of RNA foci and dipeptide formation^37^. The expression of this construct did not result in any obvious difference in F-actin levels at the growth cone under our experimental conditions (Supplementary Fig. 13). However, impaired actin dynamics due to decreased functional cofilin were previously described in primary MNs and patient-derived cells and tissues harboring a G4C2 repeat expansion in the *C9ORF72* locus^38^. As anticipated, expression of (G4C2)_80_ repeats in MNs led to the loss of RanGAP1 localization to the NE with no change to its overall levels (Fig. 9a and Supplementary Fig. 13)^6^. Interestingly, overexpression of the constitutively active form of mDia1 fully rescued the phenotype. To investigate whether these effects would occur also in a more relevant disease model such as ALS patient-derived cells, we treated fibroblasts obtained from 3 patients carrying the *C9ORF72* repeat expansion and 3 healthy controls with the formin activator IMM01. We found that a higher percentage of the C9-ALS fibroblasts had disrupted or abnormal staining for both proteins (Fig. 9b), as previously reported^1,2,6^. Similar to what was observed with mDia1 overexpression, IMM01 treatment was able to rescue defects in the localization of RanGAP1 and FG-Nups, without changes to the propensity of the cell to form *C9ORF72*-positive nuclear foci (Supplementary Fig. 14). Notably, mDia1 expression was also able to rescue functional import defects in cortical neurons expressing (G4C2)_80_ repeats as detected by S-mCherry dynamics (Fig. 9c–e). Together, these results suggest that modulation of actin homeostasis directly modifies the stability of the NPC and/or NE, protecting it from disruption caused by ALS-associated gene mutations.

## Discussion

ALS is a heterogeneous disease and mutations in a wide array of genes are associated with ALS pathogenesis^20^. Although a common mechanism linking these different gene mutations may not be immediately apparent, recent findings from several groups point at defects in NCT as central to ALS pathology^39^. Here, we explore the hypothesis that alterations in cytoskeletal stability caused by ALS-associated mutations in the actin-binding protein PFN1 would similarly impact the function of the NPC.

Our immunofluorescence data in primary MNs show that mutant PFN1 leads to a severe impairment of the NPC structure and/or number, marked by the loss of nucleoporins from the nuclear envelope, as well as of its function, as indicated by the dissolution of the nucleocytoplasmic gradient of Ran, reduced import rates, and cytosolic mislocalization of nuclear RBPs TDP-43 and FUS. No changes in the distribution of cytoplasmic shuttling proteins (i.e., SMN, FMRP, S-mCherry, and PFN1 itself) were detected, suggesting that mutant PFN1 may have a stronger effect on nuclear import versus export. The presence of PFN1 aggregates correlated with the severity of the phenotypes observed but did not directly cause these defects. Indeed, while the more aggregation prone PFN1^C71G^ mutant led to more severe phenotypes compared to PFN1^G118V^, the same defects were observed in both lines and in the absence of visible inclusion. Importantly, similar defects to the NPC were also observed in patient-derived lymphoblast cells, which we have previously shown do not contain any PFN1 positive inclusions^16^. Lymphoblast cells are actively dividing and thus reassembling the nuclear envelope and refreshing the pool of nuclear pores at every cell division, which could explain why the observed phenotypes were more subtle compared to post-mitotic neurons. However, we cannot fully exclude a dose-dependent effect of mutant PFN1 expression on the mislocalization of these nucleoporins. Regardless, our data support the hypothesis that mutant PFN1 expression causes alteration to the nuclear pore possibly leading to neuronal degeneration.

To investigate how mutant PFN1 disrupts the NCT, we tested whether it directly affected RanGAP1 protein levels or its association with the nuclear pore, as suggested in flies where loss of function of PFN1 ortholog *Chic* was associated with selective RanGAP1 irregular localization to the NE^40^. However, we did not detect any change in RanGAP1 levels or SUMOylation, which is necessary for its binding to the NPC. Next, we investigated whether PFN1 aggregation could sequester NCT factors, thus causing impairment in nuclear shuttling as shown for synthetic amyloid aggregates and TDP-43^1,13^. Again, we were not able to detect increased insolubility or co-aggregation of the factors tested with PFN1 inclusions. Additionally, while TDP-43 aggregates are cross-seeded by PFN1^16,41^, we observed mislocalization of nucleoporins and Ran even in the absence of PFN1 inclusions, suggesting that TDP-43 mislocalization is a consequence rather than the cause of NCT disturbance. Finally, we excluded from our analyses any of the rare cells with spontaneous stress granule-like formation due to PFN1 overexpression^42^, thus eliminating the possibility that stress granules would induce NCT defects in our experimental system^14^.

A more likely scenario is that mutant PFN1-dependent disruption of the actin cytoskeleton destabilizes the nuclear membrane, leading to NCT defects. This is supported by the observation that actin depolymerization caused by Latrunculin A was sufficient to cause severe changes in the cellular distribution of Ran, FG-Nups, and RanGAP1. Consistently, our data suggest that excessive actin polymerization is also detrimental to the NPC function. Indeed, PFN1^WT^ overexpression by itself caused minor defects to the staining profile of Lamin A/C and reduced nuclear import rates compared to GFP-transfected controls. Further induction of actin polymerization via mDia1 activation in PFN1^WT^ cells also worsen many of the observed phenotypes, leading to NPC structural and functional changes. Restoring normal actin homeostasis in mutant PFN1 cells, by genetic or pharmacological induction of mDia1 function was able to structurally and functionally rescue the NPC. Intriguingly, we found that formin-dependent rescue was not limited to mutant PFN1 cells, but extended to cells expressing the *C9ORF72* repeat expansion, the most common mutation found in ALS and FTD patients, in which NPC defects have been largely reported^2,3,6^. Although the mechanisms driving this rescue are not fully understood yet, we speculate that actin may modulate the integrity of the NE via the function of the linker of nucleoskeleton and cytoskeleton (LINC) complex^43^, a multiprotein complex that physically connects lamins with actin and microtubules. Of note, mutations in LINC components cause cerebellar ataxia^44,45^, suggesting that this complex plays an important role in the maintenance of neuronal function and survival, possibly through the modulation of NPC function. A similar pathogenic mechanism has been recently suggested for tau-dependent neurodegeneration, where altered microtubule polymerization led to increased nuclear invaginations and NCT defects^46–48^. Future work will be required to reveal how changes to actin and, more generally, cytoskeleton homeostasis affect the LINC complex and its functional consequences on the NCT and how this pathway could be employed as a strategy to modulate NCT function.

It remains an open question whether the NCT defects observed in our model and others are a cause or a mere effect of the degenerative process. Our data argue for the NPC dysfunction being a primary driving force of ALS-associated cell toxicity. Indeed, we found that pharmacological inhibition of nuclear export rescued PFN1-dependent cellular defects, such as stunted axon growth. Together with previous observation connecting deficiencies in NCT to axonal guidance defects in flies^49^, our data are particularly interesting as they functionally link the cytoskeleton with NCT and mRNA post-transcriptional regulation. Axon growth is a complex process that requires active cytoskeletal growth^50^, fueled by the increased expression, transport, and local translation of several cytoskeletal-related mRNAs^51,52^. Defects in the localization and local translation of mRNAs at the tip of axons and dendrites has been described in other neurological diseases, such as SMA and Fragile X syndrome^53,54^, supporting the relevance of these defects in disease pathogenesis. Our data suggest that PFN1-dependent impairment of NCT and consequent mislocalization/loss of function of nuclear RBPs leads to impaired mRNA regulation, of which *POLDIP3* pre-mRNA splicing and *Nefl* mRNA axonal localization are two examples. Rescue of the NPC function via modulation of the actin cytoskeleton also leads to rescue in the *Nefl* mRNA localization to the axon, which support our hypothesis that PFN1-mediated and actin-dependent disruption of NCT may have severe downstream consequences on general mRNA regulation, leading to a vicious cycle that eventually leads to MN degeneration. Rescue in either of these pathways would positively impact the others possibly preserving MNs in ALS.

In conclusion, our results suggest a model whereby modulations to actin dynamics can affect the stability and function of the nuclear pore, adding cytoskeletal function to the list of modulators of NCT, together with protein aggregation and oxidative stress^1,14^. The defects in NCT observed in ALS MNs could be particularly relevant to disease pathogenesis as it may compromise many cellular pathways relying on the integration between signaling and changes in gene expression and/or RNA processing, such as neurotrophic factors signaling or oxidative stress^55–57^. Failure to integrate these signaling cascades would decrease the cell’s ability to respond to environmental challenges, leading to the accumulation of toxic insults. Regulating nuclear export or restoring NPC function via modulation of the cytoskeleton-nucleus connection could represent feasible therapeutic strategies for multiple forms of ALS and other neurodegenerative diseases beyond ALS.

## Methods

### Primary motor neuron culture, transfection, and treatments

Primary motor neurons (MNs) were isolated from E12.5 mouse embryonic spinal cords dissociated in 0.1% trypsin (Worthington) at 37 °C for 12 min. MNs were purified using a 6% Optiprep (Sigma-Aldrich) density gradient and plated on glass coverslips coated with 0.5 g/L poly-ornithine (Sigma-Aldrich) and laminin (Thermo Fisher). Cells were grown at 37 °C and 5% CO_2_ in Neurobasal medium (Thermo Fisher) supplemented with 0.25% Glutamax, 2% B27, 2% horse serum, and 10 ng/ml BDNF, GDNF, and CNTF. MNs at 2 days in vitro (DIV) were transfected using 1.75 µl NeuroMag reagent (OZ Biosciences) + 0.5 µg DNA. Complete growth medium was replaced with serum-free neurobasal medium 1 h prior and after transfection. The paramagnetic nanobeads used for transfection can linger inside the cell and in the extracellular space for several days and can be detected as DAPI positive dots. The V5-PFN1 plasmids were described in Wu et al. (2011), while the (G4C2)_80_ construct was described in ref. ^37^. For the GFP-PFN1 constructs, WT or mutant PFN1 was PCR amplified from the V5 constructs and cloned in the pEGFP-C1 backbone using BamHI sites. The FH1-FH2 domains of murine mDia1 (aa 553–1192) were PCR amplified using specific primers and cloned into the pEGFP-C1 plasmid using XhoI and BamHI restriction sites. GFP or GFP-mDia1 were co-transfected in a 1:2 ratio with PFN1 or *C9ORF72* plasmids. Mock transfection controls were performed by subjecting the cells to all the steps of transfection without adding any plasmid DNA. For actin depolymerization assays, 2DIV MNs were treated with 0.1 mg/L Latrunculin A (Cayman) for 3 days. For nuclear export inhibition, MNs were treated with 50 nM KPT-276 (Selleck) or equal volume of DMSO (Sigma Aldrich) 1 day after transfection. For formin induction, MNs were treated with 0.1 µM IMM01 (EMD Millipore) for 24 h before fixation at 6DIV.

### Lymphoblast cells culture

Immortalized lymphoblastoid cell lines obtained from 3 ALS patients carrying PFN1 mutations and 4 controls^16^ were cultured in RPMI medium supplemented with 15% fetal bovine serum (FBS) for a maximum of 10 passages. Cells were seeded on a poly-ornithin coated coverslip at the density of 300 cell/mm^2^. Thirty minutes after plating, cells were fixed in 4% paraformaldehyde and processed for immunofluorescence assays as described below.

### Cortical neuron culture, transfection, and treatments

Primary cortical neurons were isolated from E15 mouse embryos dissociated in 0.05% trypsin-EDTA (Thermo Scientific) at 37 °C for 12 min. 320,000 cells/ml were plated on poly-D-lysine (0.125 mg/ml, Sigma Aldrich) and lamin (5 µg/ml, Corning) coated 96-well glass plates and grown at 37 °C and 5% CO_2_ in Neurobasal medium (Thermo Fisher) supplemented with 2% B27 and 1% Glutamax (Thermo Scientific). Four DIV neurons were transfected with 0.2 µl Lipofectamine 2000 (Thermo Scientific) and 100 ng DNA following manufacturer’s recommendations. A ratio of 4:1 was used for the GFP and S-mCherry vectors. Thirsty-six hours after transfection, Leptomycin B (10 mg/L) was added to the culture medium immediately before image acquisition. Cells were imaged using a Nikon TiE widefield microscope equipped with temperature- and CO_2_-controlled environmental chamber. Movies were acquired with a ×20 lens at a rate of 1 frame every 1 or 2 min for 1 h. For rescue experiments, neurons were fixed immediately after acquisition and stained with V5 antibody to detect V5-PFN1 expression.

### Fibroblast culture, treatment, and immunofluorescence

Fibroblasts were obtained from skin biopsies of 3 ALS patients carrying mutations in *C9ORF72* gene and of 3 healthy donors, sex- and age-matched with ALS cases, after informed consent and approval by the IRCCS Istituto Auxologico Italiano ethics committee. *C9ORF72* mutation was validated in primary fibroblasts by repeat-primed PCR as previously described^58^ and repeat expansion size determined by Southern blot analysis (range 600–1500 units). Fibroblasts were grown in RPMI 1640 medium (EuroClone) containing 2 g/l glucose and supplemented with 10% FBS (Sigma Aldrich), 2 mM L-glutamine, 2,5 μg/ml amphotericin B (Sigma Aldrich), 100 units/ml penicillin and 100 μg/ml streptomycin (Gibco). 10.000 cell/well were seeded on glass coverslip in 24-well plates and after 24 h, 50% of medium was replaced with fresh medium and IMM01 (Sigma Aldrich) at the final concentration of 0.1 µM. After 24-h treatment, cells were fixed with 4% paraformaldehyde for 20 min, permeabilized with 0.3% Triton X-100/1× PBS for 5 min and processed for immunofluorescence as described below.

### Immunofluorescence and image acquisition

Cells were fixed with 4% paraformaldehyde for 15 min. Fixed motor neurons were treated with hot 10 mM citrate buffer, pH 6 for 20 min before permeabilization with 0.2% Triton-X 100 for 5 min. Cells were blocked with 5% bovine serum albumin for 45 min and hybridized with the appropriate antibodies overnight at 4 °C. Anti-mouse and anti-rabbit donkey secondary antibodies or phalloidin conjugated with either Alexa Fluor 647, Alexa Fluor 594, Alexa Fluor 555, Alexa Fluor 546, or Alexa Fluor 488 (Jackson Immunoresearch and Thermo Fisher) were hybridized for 1 h at room temperature. Coverslips were mounted onto a glass slide using Prolong Gold mounting medium (Thermo Fisher) or FluorSave mounting medium (Calbiochem) and imaged using an epifluorescence microscope (Nikon Ti E) equipped with a cooled CMOS camera (Andor Zyla) or a Digital sight DS-U3 camera. Images were acquired as Z-stacks (0.2 µm step size) using a ×60 lens unless otherwise specified. For propidium iodide (PI) staining, cells were incubated with 20 µg/ml PI (Thermo Fisher) for 30 min at 37 °C before fixation. As a positive control, cells were heat shocked at 65 °C for 30 min before incubation with PI.

### Transmission electron microscopy

Six-well plates of cultured cells were fixed overnight at 4 °C by adding 1 ml of 2.5% glutaraldehyde in 0.1 M Na cacodylate-HCl buffer (pH 7.2). Fixed samples were washed three times in 0.5 M Na cacodylate-HCl buffer (pH 7.0) and post-fixed for 1 h in 1% osmium tetroxide (w/v) in the same buffer at room temperature. Following post-fixation, the culture dish with adherent cells were enblock stained (20 min) with 1% aqueous uranyl-acetate (w/v). The fixed cell culture dishes were washed again in the same buffer and dehydrated through a graded series of ethanol to 100% and transferred through two changes of 50/50 (v/v) SPIpon resin (Structure Probe, Inc.)/100% ethanol and left overnight to infiltrate. The following morning the cell culture plates were transferred through three changes of fresh SPIpon resin to finish the infiltration and embedding and finally, the dishes were filled with freshly prepared SPIpon resin and polymerized for 48 h at 70 °C. Once fully polymerized, the six-well-plate was cut apart and each well was plunged into liquid nitrogen to separate the SPIpon epoxy block with the embedded cells from the culture dish. The round epoxy disks with the embedded cells were then examined under an upright light microscope and areas of cells were cut from the disks and glued onto blank microtome studs and trimmed for ultramicrotomy. Ultrathin sections (70 nm) were cut on a Reichart-Jung ultramicrotome using a diamond knife. The sections were collected and mounted on copper support grids and contrasted with lead citrate and uranyl acetate and examined on a FEI Tecnai G2 Spirit transmission electron microscope at 100 Kv accelerating voltage and images were recorded at various magnifications using a Gatan 2 K digital camera system.

### Fluorescence in situ hybridization

Motor neurons were fixed in 4% RNase-free paraformaldehyde for 15 minutes and stored in 70% ethanol at 4 °C overnight. Cells were sequentially incubated for 5 min in 1× PBS and wash buffer (2× SSC, 10% formamide), and hybridized in hybridization buffer (10 mg/ml dextran sulfate, 4 mg/ml BSA, 40 µM ribonucleoside vanadyl complexes, 2× SSC, 1% PBS) at 37 °C overnight with 12.5 µM probes and 0.2 mg/ml each salmon sperm DNA and *E. Coli* tRNA. *Neurofilament L* mRNA specific probes were design using Biosearch Technology online tool and conjugated with the Quasar® 570 fluorophore (Table S2). Coverslips were then washed twice for 30 min at 37 °C in wash buffer before mounting them as described above. FISH for *C9ORF72* sense RNA hexanucleotide repeat was performed using the LNA probe 5’TYE563/CCCCGGCCCCGGCCCC (Exiqon) as described in Chew et al.^59^.

### Axon length and outgrowth analysis

Motor neurons were co-transfected as outlined above with green fluorescent protein (GFP) and V5-tagged PFN1 constructs in a 1:2 ratio. KPT-276 (50 nM) or equal volumes of DMSO were added to the culture medium 1 day after transfection and maintained throughout the experiment for a total of 3 days. Cells were fixed and stained to detect V5-PFN1 expression 4 days after transfection. Cells were imaged as individual focal planes using a ×10 lens. GFP was used to identify transfected cells and highlight the whole cell structure. For live imaging of axon outgrowth, KPT-276 (50 nM) or equal volumes of DMSO were added to the culture medium 1 day after transfection and maintained throughout the experiment for a total of ~18–24 h. Cells were imaged at 3 DIV using a Nikon TiE widefield microscope equipped with temperature- and CO_2_-controlled environmental chamber. Movies were acquired with a ×20 lens at a rate of 1 frame every 10 min for 1 h.

### Immunoprecipitations, solubility assays, and western blots

HEK293 cells grown in DMEM + 10% FBS were transfected with V5-PFN1 constructs and lysed with lysis buffer (20 mM Tris, 150 mM NaCl, 1% Triton X-100, protease inhibitor Complete EDTA-free, Roche) 24 or 48 h after transfection. For immunoprecipitations, the detergent-soluble lysates were added to 30 µL of Protein A-Agarose beads (Roche) and 0.3 µg of anti-V5 antibody (Novus) and rocked overnight at 4 °C. Immunoprecipitated complexes were eluted in Laemmli buffer (60 mM Tris-Cl pH 6.8, 2% SDS, 10% glycerol, 5% beta-mercaptoethanol, 0.01% bromophenol blue) and then subjected to western blot analysis. For solubility assays, lysates were sonicated on ice and then centrifuged at 16,000 × *g* for 10 min at 4 °C. The supernatant was collected as the soluble fraction. The pellet was washed three times with lysis buffer, resuspended in 8 M urea, sonicated, and then centrifuged at 16,000 × *g* for 10 min at 4 °C. The supernatant was collected as the insoluble fraction.

Samples were resolved by SDS-PAGE on Mini Protean TGX 4–20% gradient polyacrylamide gels (Bio-Rad) and transferred onto nitrocellulose membranes (Bio-Rad). Membranes were blocked with Odyssey Blocking Buffer (LI-COR) and probed with primary antibodies overnight. Secondary antibodies conjugated with IRDye^®^ infrared fluorophores (LI-COR) were incubated for 1 h at room temperature. Blots were visualized using the Odyssey Infrared Imaging System (LI-COR). Uncropped scans of the blots are shown in Supplementary Fig. 15.

### POLDIP3 splicing assay

Whole RNA was extracted from 5 × 10^6^ lymphoblast cells using TriZol reagent (Thermo Fisher) according to the manufacturer’s instructions. RNA (2 µg) was retrotranscribed using the High-Capacity cDNA Reverse Transcription Kit (Thermo Fisher). RT-PCR was performed using specific primers amplifying exon2-exon4 of human *POLDIP3* mRNA. DNA gels were stained with SYBR Safe dye (Thermo Fisher) and imaged using the ChemiDoc XR+ imager (BioRad).

### Image processing and quantification

Immunofluorescence images were deconvolved using an adaptative blind deconvolution algorithm (Autoquant X3, Media Cybernetics) before analysis. To measure fluorescence intensities, the signals were thresholded and the resulting integrated densities were normalized on the area of the selected region (e.g., cell body, nucleus). Thresholds were kept consistent for all images within experiments. For all experiments, values were normalized to PFN1^WT^ averages so that PFN1^WT^ always = 1 ± SEM. For NCT dynamics, the fluorescence intensity of S-mCherry was measured in the nucleus using ImageJ and normalized to the background for every time point. All values were subsequently normalized to T0. For the analysis of axonal fluorescence intensities, a 100 µm long region of the proximal axon was measured in all conditions. Axon lengths were measured using the ImageJ plugin NeuronJ^60^. The axon was defined as the longest neurite. The rate of axon outgrowth was measured using the ImageJ plugin MTrackJ by tracking the movement of the growth cone in all fields^61,62^. A blinded analysis was performed to assess RanGAP1, RanBP2, POM121, F-Nups, and Lamin A/C localization on 3D stacks of individual optical slices. To avoid misinterpretation of staining profiles, no 3D to 2D compression of the images was performed as suggested^63,64^. For nucleoporin analysis, abnormal staining was considered if the signal was not uniformly distributed around the nucleus with the presence of empty bare segments. For Lamin A/C, abnormal staining was defined as the absence of a think and uniform layer around the nucleus. DAPI was used as a reference for nuclear boundary. For all experiments, raw values were normalized to the mean of the control condition.

### Nuclear dextran assay

Nuclei were isolated from lymphoblasts and then incubated with fluorescently labeled-Dextrans as described with minor modifications^65,66^. Briefly, 10^6^ cells were pelleted by centrifugation for 5 min at 4 °C at 2600 RPM and resuspended in 1 mL of sucrose buffer (0.32 M sucrose, 3 mM CaCl_2_, 2 mM magnesium acetate, 0.1 mM EDTA, 10 mM Tris HCl, 1 mM dithiothreitol, and protease inhibitors (Roche, cat # 11873580001)) with 1% NP-40. After 20 min incubation on ice, the cells were washed in sucrose buffer and centrifuged for 5 min at 2600 RPM at 4 °C. The pellet was washed in 1 mL of TR buffer (20 mM HEPES, 110 mM KOAc, 2 mM Mg(OAc)_2_, 5 mM NaOAc, 0.5 mM EGTA, 250 mM sucrose, and protease inhibitors) and the isolated nuclei were then incubated for 30 min in 100 uL of TRB containing 0.6 mg/mL of 70 KDa RITC-Dextran (Millipore Sigma, R9379), 0.6 mg/mL of 500 KDa FITC-Dextran (Millipore Sigma, 46947), and Dapi and imaged using confocal microscopy (A1R, Nikon). Images were analyzed using image J. RITC-dextran intensity in the nucleus was defined as the ratio of the nuclear mean intensity to the background intensity. Nuclei in which the RITC-dextran intensity was greater than the mean RITC-dextran intensity of all control nuclei plus 2 standard deviations were classified as leaky nuclei.

### Statistical analysis

Statistical analyses were performed using Prism 8 software package (GraphPad). Normality of the samples was assessed using the D’Agostino & Pearson Omnibus test. According to normality, parametric or non-parametric tests were used to assess significance, defined as *p* < 0.05. For nuclear import assays, the slope was calculated on the curve averages as shown in the graphs. Detailed information on all statistical tests performed is listed in Table S1.

### Reporting summary

Further information on research design is available in the Nature Research Reporting Summary linked to this article.

## Supplementary information

Supplementary Information

Peer Review

Reporting Summary

Description of Additional Supplementary Files

Supplementary Movie 1

## Data Availability

The datasets generated during the current study are available from the corresponding author on reasonable request.
