# Supplementary material for: Modulation of actin polymerization affects nucleocytoplasmic transport in multiple forms of amyotrophic lateral sclerosis
Source: Nat Commun. 2019 Aug 23;10:3827. doi: 10.1038/s41467-019-11837-y (PMC6707192; doi:10.1038/s41467-019-11837-y)
Supplement: Supplementary file 1 — Supplementary Information [file 41467_2019_11837_MOESM1_ESM.pdf]

Supplementary Material

Modulation of actin polymerization affects nucleocytoplasmic transport in multiple forms of ALS

Anthony Giampetruzzi, Eric W. Danielson, Valentina Gumina, Maryangel Jeon, Sivakumar Boopathy, Robert H. Brown, Antonia Ratti, John E. Landers, Claudia Fallini

Content:

- **Supplementary Figure 1.** Characterization of V5-PFN1 expression in primary motor neurons.
- **Supplementary Figure 2.** Expression of V5-PFN1^{WT} does not affect the cellular localization of endogenous Nups and RBPs.
- **Supplementary Figure 3.** Mutant PFN1 alters RanBP2 association with the nuclear envelope.
- **Supplementary Figure 4.** Effect of mutant PFN1 expression on the levels of NPC and NCT factors.
- **Supplementary Figure 5.** Karyopherins distribution is not affected by mutant PFN1.
- **Supplementary Figure 6.** Nucleoporin levels and membrane integrity are not altered in PFN1 mutant lymphoblast lines.
- **Supplementary Figure 7.** Mutant PFN1 effect on cellular levels of nuclear RNA-binding proteins.
- **Supplementary Figure 8.** Mutant PFN1 effect on cellular levels of cytoplasmic RNA-binding proteins.
- **Supplementary Figure 9.** Mutant PFN1 causes increased TDP-43 aggregation.
- **Supplementary Figure 10.** Control experiments for TDP-43 dependent mRNA regulation.
- **Supplementary Figure 11.** Characterization of motor neurons following latrunculin A treatment
- **Supplementary Figure 12.** mDia1 overexpression rescues actin polymerization in mutant PFN1 MNs
- **Supplementary Figure 13.** Expression of $(G4C2)_{80}$ does not alter F-actin levels at growth cones.
- **Supplementary Figure 14.** IMM01 does not alter foci formation in C9-ALS fibroblasts.
- **Supplementary Figure 15.** Original uncropped scans for western blot assays.
- **Supplementary Table 1.** Summary of experimental data and statistical analyses.
- **Supplementary Table 2.** List of critical reagents and resources.
- **Supplementary Table 3.** Sequence information for the *Nefl* mRNA FISH probes.

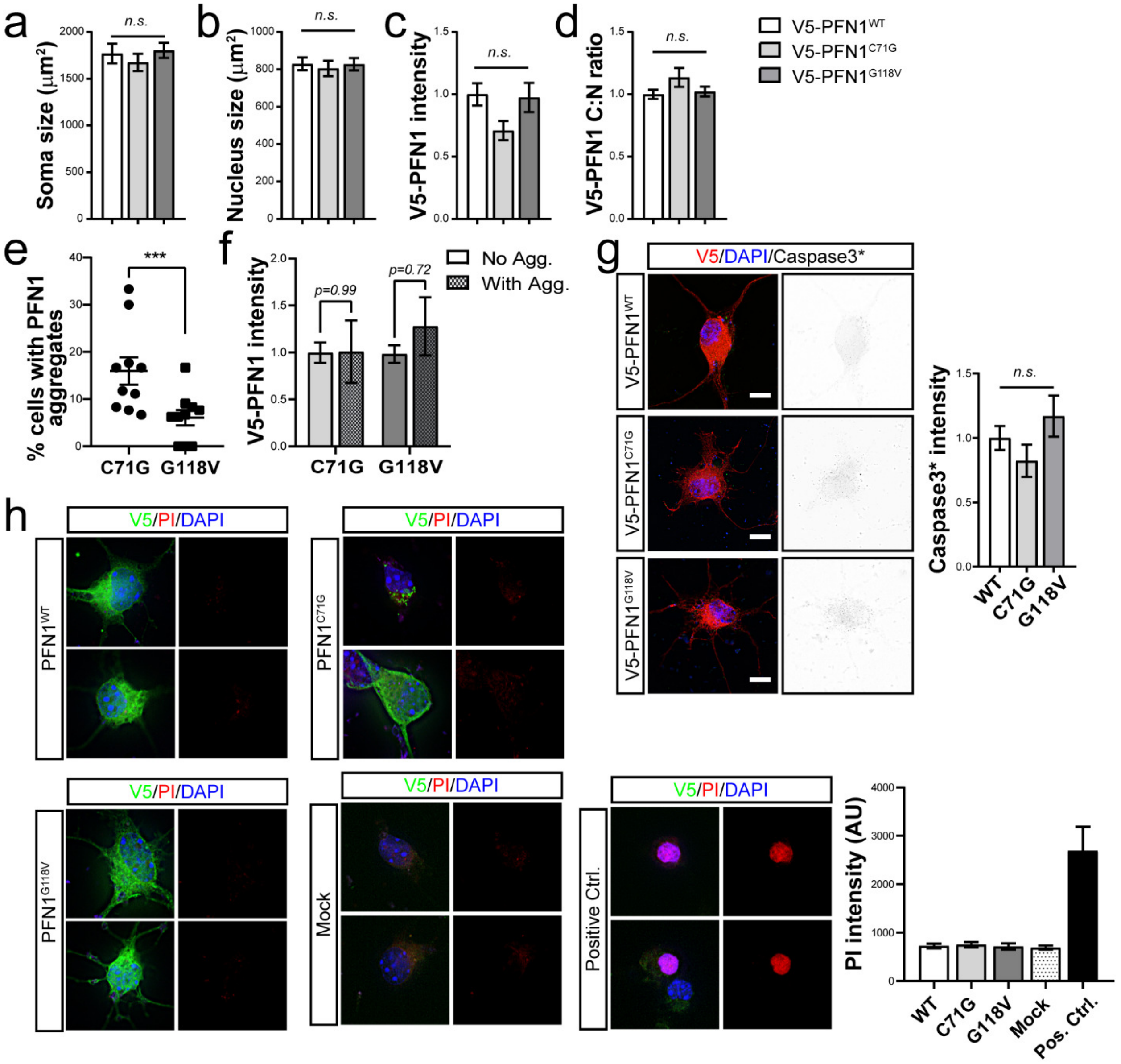

Supplementary Figure 1. Characterization of V5-PFN1 expression in primary motor neurons. *Related to Figure 1.*

a-b. Primary motor neurons were transfected with expression plasmids encoding wild type (WT) or mutant (C71G, G118V) PFN1 (red). No alterations to the size of the cell soma and nucleus were observed due to the expression of mutant PFN1. **c-f.** The expression level and localization of V5-tagged PFN1 were measured and compared. No significant difference was detected across conditions. As expected, PFN1^{C71G} mutant cells had significantly more frequent aggregates compared to PFN1^{G118V} neurons. No difference in PFN1 levels was observed between cells with or without visible aggregates. **g-h.** Finally, no increase in the apoptotic pathway or necrotic death was observed due to the expression of WT or mutant PFN1 as indicated by lack of active Caspase3* staining (g, *black*) or propidium iodide (PI) incorporation (*h*, red). DAPI (*blue*) was used to label the nuclei. Scale bars: 10µm. Bars are mean±SEM; one way-ANOVA; n=54, 45, 60 cells for WT, C71G and G118V respectively for V5-PFN1 analyses; n= 39, 41, 43 cells for WT, C71G and G118V respectively for Caspase3 analysis; n= 21, 23, 21, 14, 9 for WT, C71G, G118V, mock, and positive control (i.e. heat shock), respectively for PI staining.

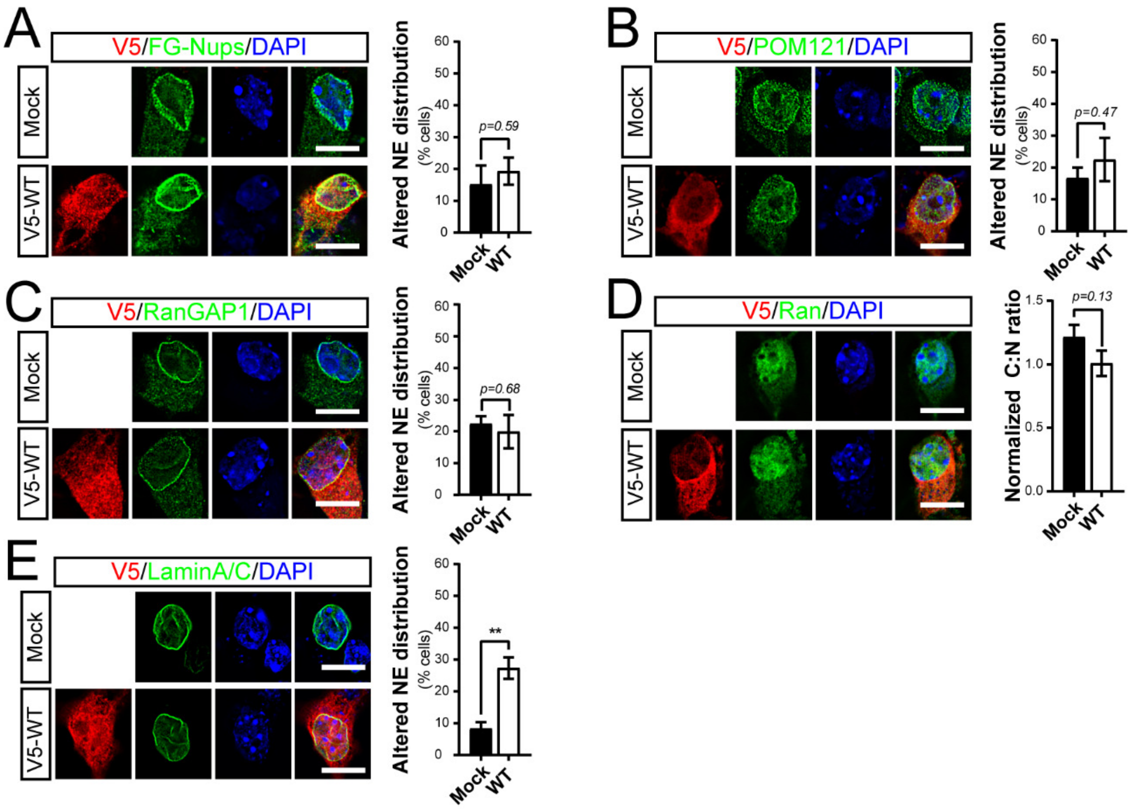

Supplementary Figure 2. Expression of V5-PFN1^{WT} does not affect the cellular localization of endogenous Nups and RBPs. *Related to Figures 1 and 2.*

Primary motor neurons expressing wild type PFN1 (red) were compared to mock transfected controls for the localization of FG-Nup (*A*, *green*), POM121 (*B*, *green*), RanGAP1 (*C*, *green*), Ran (*D*, *green*), and Lamin A/C (*E*, *green*). No difference in the distribution of the endogenous proteins was observed. DAPI (*blue*) was used to label the nuclei. Scale bars: 10µm. Bars are mean±SEM; Student's *t* test; ** $p < 0.01$; n=4 experiments in A-C, E; n= 46 cells in D.

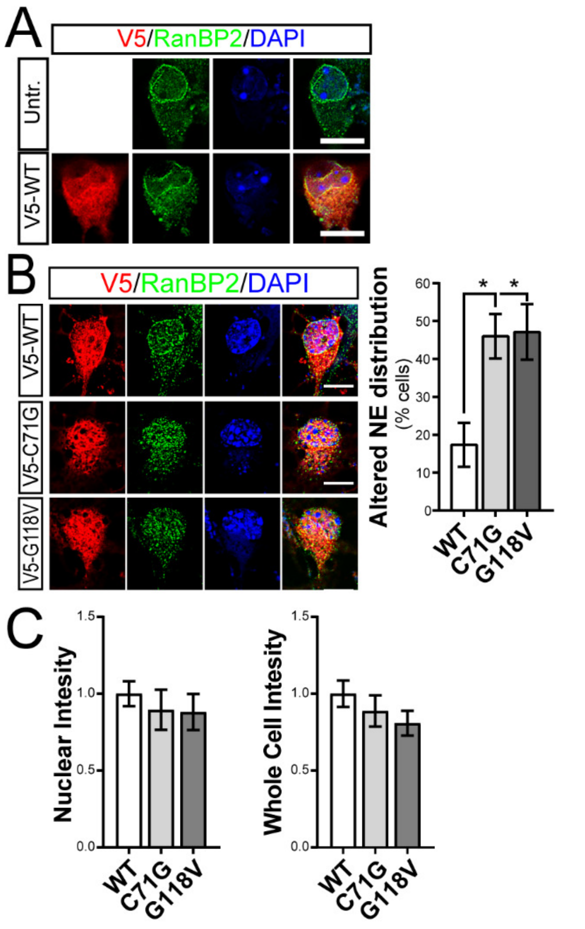

Supplementary Figure 3. Mutant PFN1 alters RanBP2 association with the nuclear envelope. *Related to Figure 1.*

A. No significant changes to RanBP2 localization to the NE were observed due to PFN1 WT expression. **B-C.** Mutant PFN1 causes an increase in RanBP2 mislocalization from the NE but did not alter its overall cellular levels. Bars are mean±SEM; paired one way-ANOVA with Dunnet post *hoc* test; n=5 independent experiments (*B*); n=22, 19, 31 cells from at least 3 independent experiments for WT, C71G and G118V, respectively (*C*).

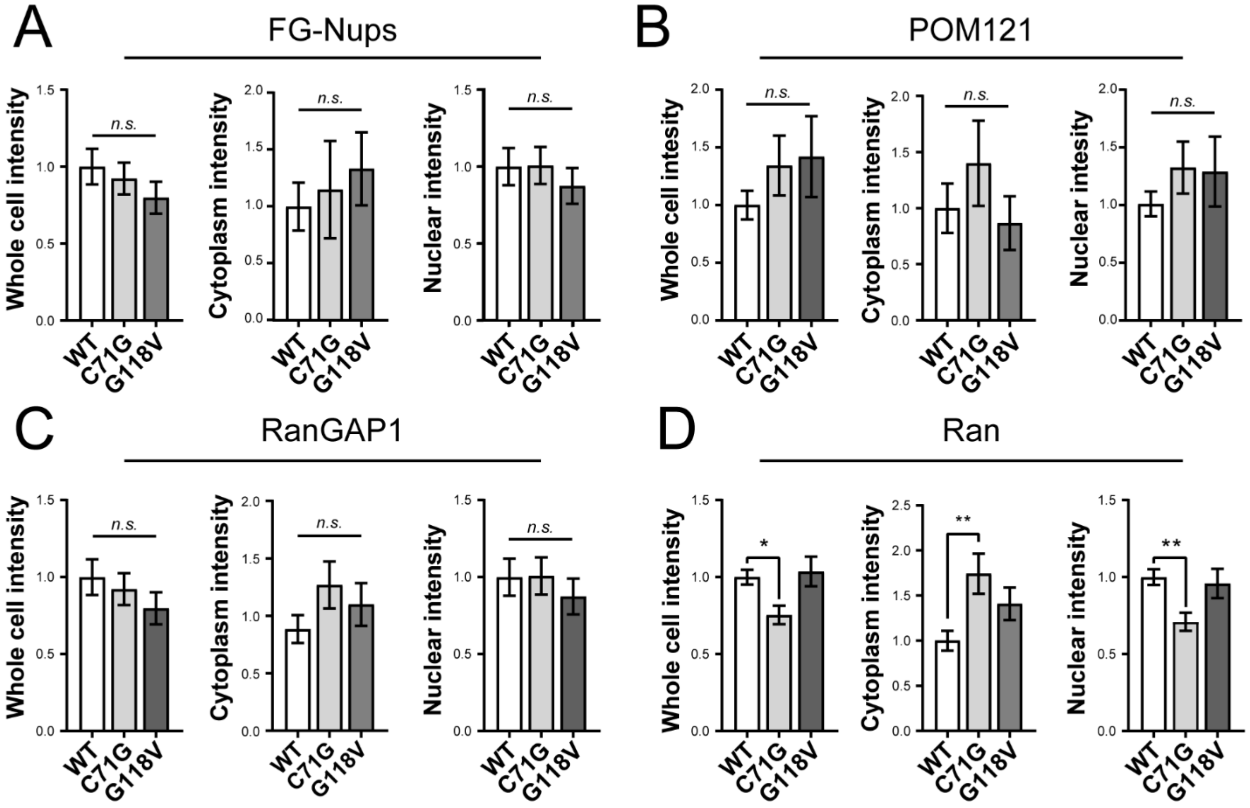

Supplementary Figure 4. Effect of mutant PFN1 expression on the levels of NPC and NCT factors. *Related to Figure 1.*

Fluorescence intensity in the whole cell, cytoplasm, and nucleus for FG-Nups (*A*), POM121 (*B*), RanGAP1(*C*), and Ran (*D*) were measured in motor neurons expressing WT or mutant PFN1. Bars are mean±SEM. One way-ANOVA, * *p*<0.05, ** *p*<0.001, *** *p*<0.001; N= 36, 30, 40 cells (*A*); 42, 34, 20 cells (*B*); 36, 30, 40 cells (*C*); 102, 84, 74 cells (*D*), for WT, C71G, and G118V, respectively.

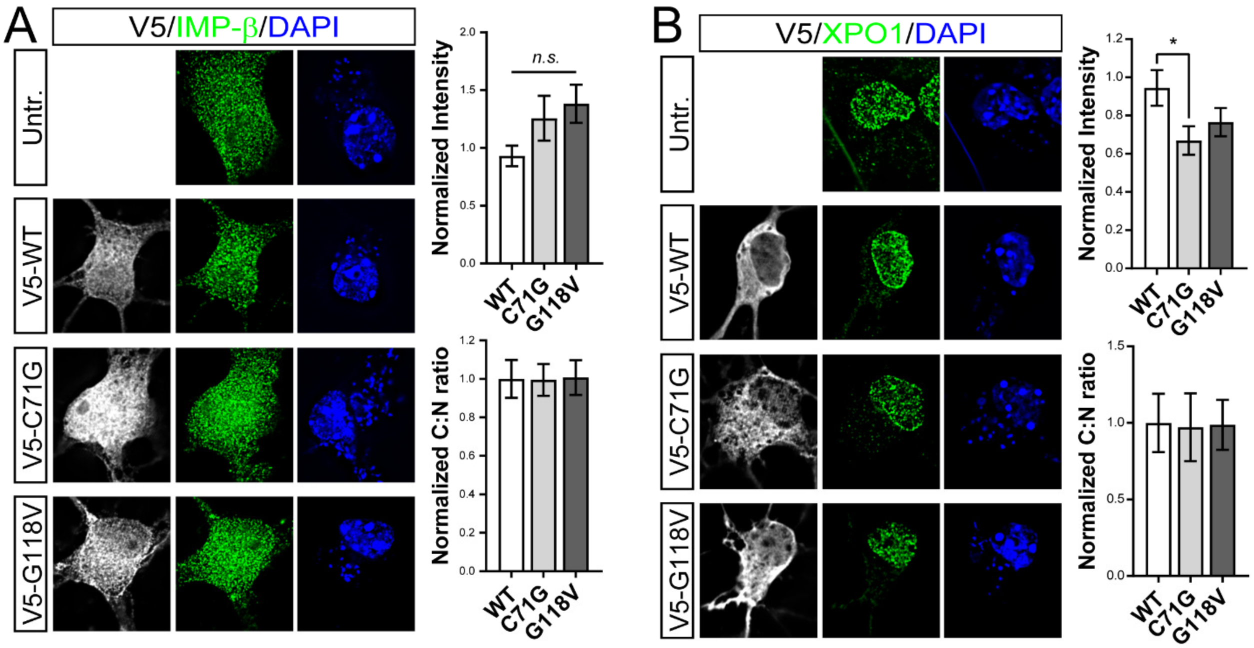

Supplementary Figure 5. Karyopherins distribution is not affected by mutant PFN1. *Related to Figure 1.* **A-B.** Representative images and quantification of Importin-β (*A, green*) and Exportin 1 (XPO1; *B, green*) overall levels (*top graph*) and C:N ratio (*bottom graph*) in motor neurons expressing wildtype or mutant PFN1. No major change in protein distribution was detected. A small but significant reduction in XPO1 overall levels was observed in $PFN1^{C71G}$-MNs. Bars are mean±SEM. One way-ANOVA, * $p<0.05$, N= 29, 26, 29 cells (*A*) and 40, 37, 43 cells (*B*) for WT, C71G, and G118V, respectively.

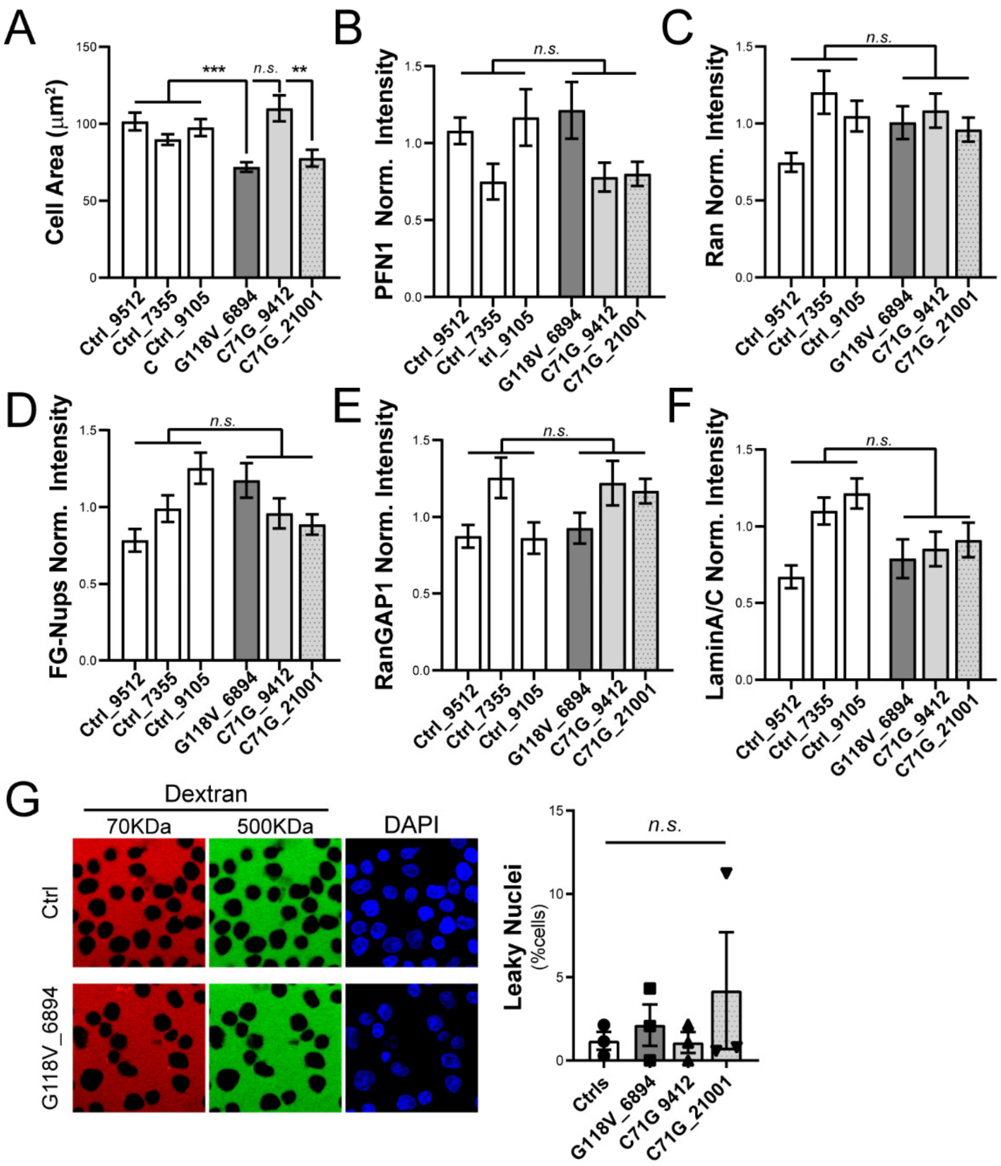

Supplementary Figure 6. Nucleoporin levels and membrane integrity are not altered in PFN1 mutant lymphoblast lines. *Related to Figure 3.*

A-F. Lymphoblast lines from 3 ALS patients and 3 controls were assessed for changes to (*A*) cell size and the overall levels of (*B*) PFN1, (*C*) Ran, (*D*) FG-Nups, (*E*) RanGAP1, and (*F*) Lamin A/C. No significant difference was observed in the levels of the assessed proteins, while a reduction is cell area was observed in 2 out of 3 ALS lines. **G.** The integrity of the nuclear membrane was assessed by the dextran exclusion assay. The nuclear levels of fluorescently conjugated dextrans were measured. Leaky nuclei were defined as having a nuclear signal for the 70KDa dextran greater than 2 standard deviations from the control average. 500KDa dextran signal and absence of DAPI staining were used to exclude nuclei damaged during the isolation. Bars are mean±SEM. One way-ANOVA, *n.s.* non-significant. N= 41-52 from 3 independent experiments from *A-F*; N= 3 experiments for *G*.

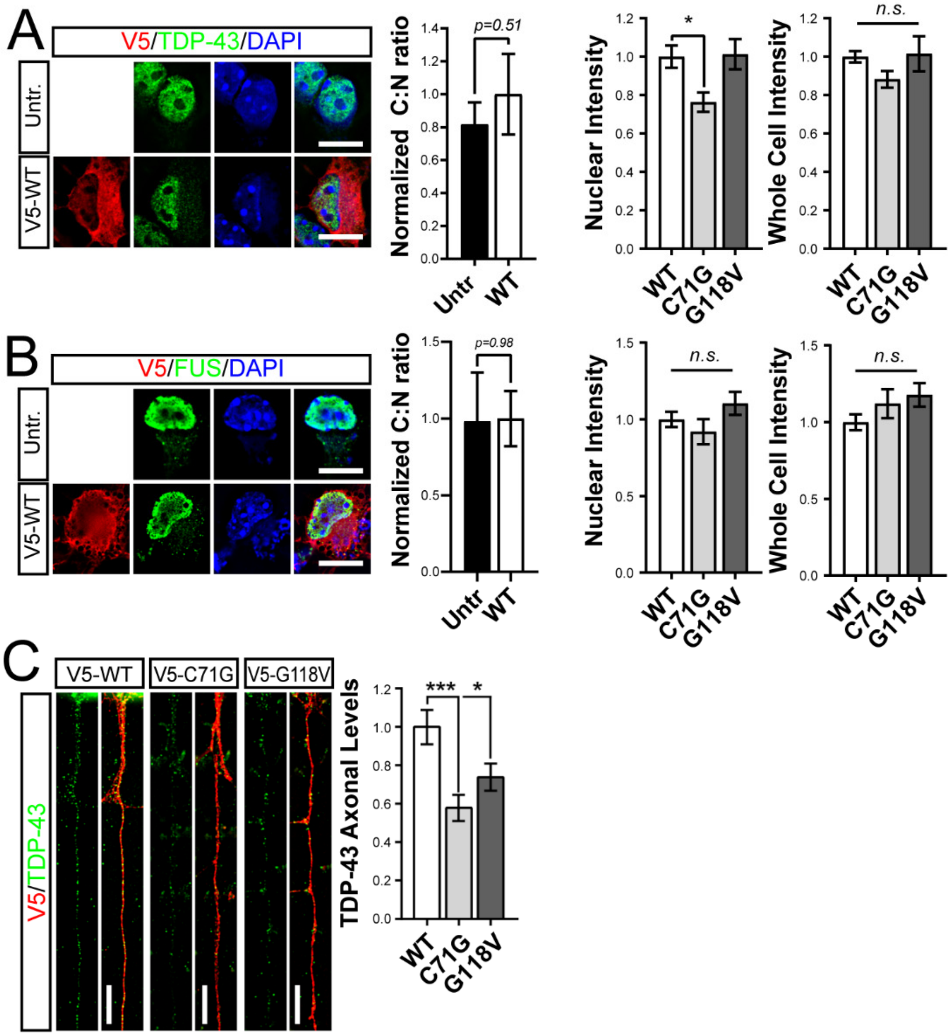

Supplementary Figure 7. Mutant PFN1 effect on cellular levels of nuclear RNA-binding proteins. *Related to Figure 5.*

While PFN1 WT (*red*) has no impact on endogenous distribution of TDP-43 (*A and C, green*) and FUS (*B, green*), mutant PFN1 causes a significant reduction in TDP-43 nuclear (*A*) and axonal levels (*C*). No changes in overall cellular levels were detected for neither TDP-43 nor FUS. Scale bars: 10µm. Bars are mean±SEM; one-way ANOVA with Dunnett's *post hoc* test; *$p<0.05$**, $p<0.01$, ***$p<0.001$; N=43, 37, 31 cells (*A*); 45, 47, 44 cells (*B*); 40, 35, 28 cells (*C*).

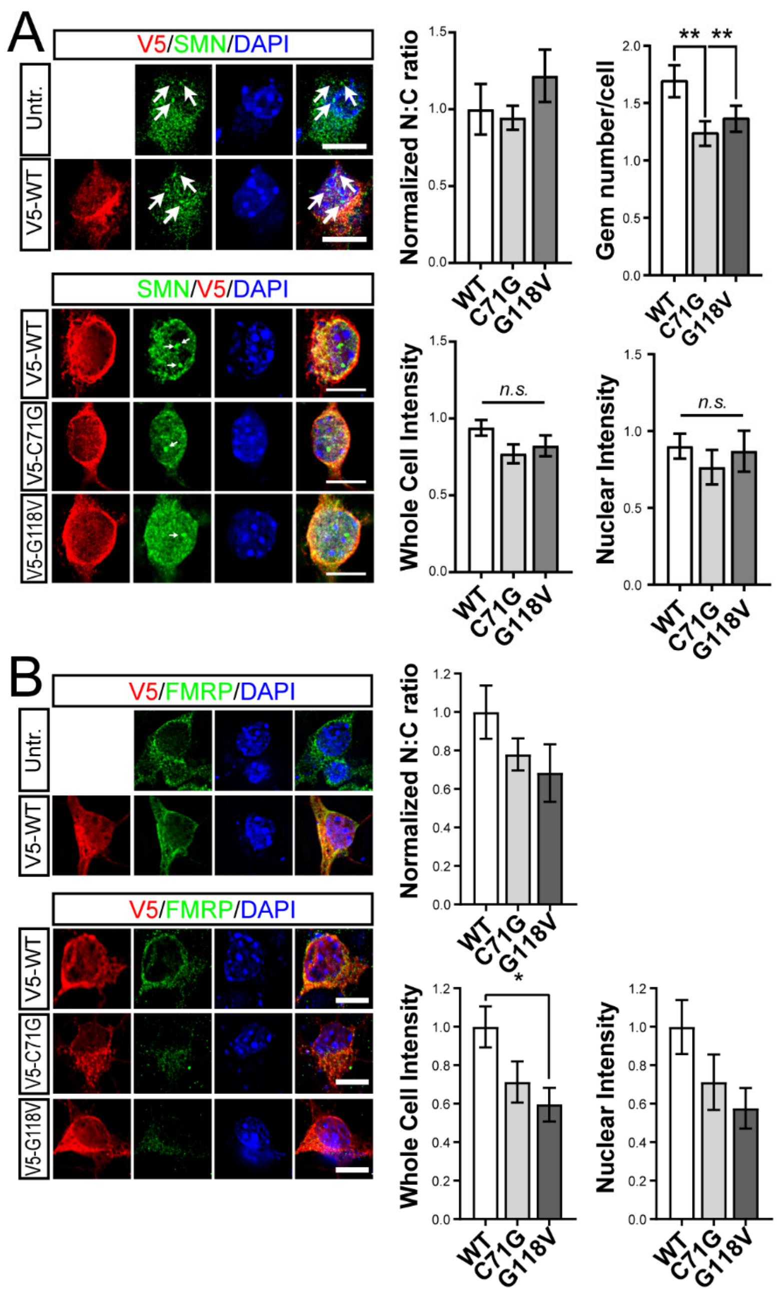

Supplementary Figure 8. Mutant PFN1 effect on cellular levels of cytoplasmic RNA-binding proteins. *Related to Figure 5.*

Representative images and quantification of SMN (*A*, *green*) and FMRP (*B, green*) levels in the cell soma and nucleus of MNs expressing WT or mutant PFN1 (*red*). While no changes in SMN overall levels were detected, a significant reduction in nuclear gems was observed (arrows). A significant decrease in FMRP levels was also detected in $PFN1^{G118V}$-MNs. Scale bars: 10µm. Bars are mean±SEM; one-way ANOVA with Dunnett's *post hoc* test; **p*<0.05**, *p*<0.01; N=43, 38, 43 cells (*A*); 52, 47, 35 cells (*B*).

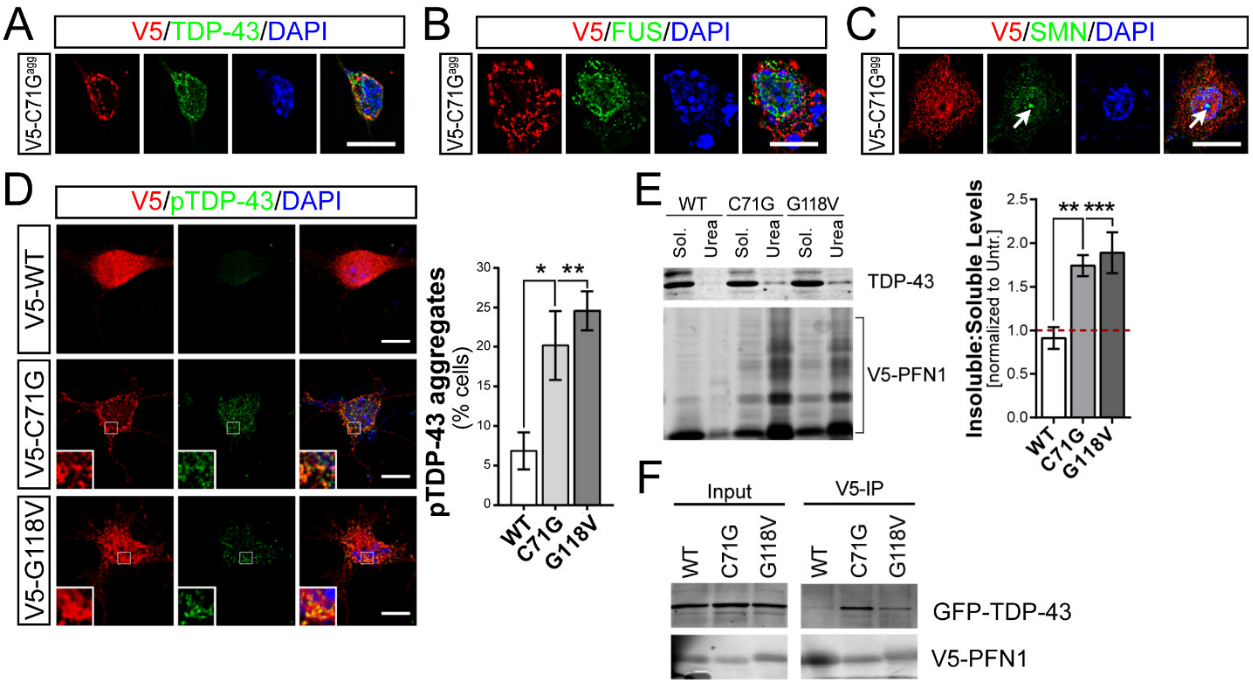

Supplementary Figure 9. Mutant PFN1 causes increased TDP-43 aggregation. *Relative to Figure 5*.
A-C. PFN1^{C71G}-positive aggregates (*red*) colocalize with TDP-43 (*A, green*) but not FUS (*B*, *green*) or SMN (*C*, *green*). **D.** Representative images of phosphorylated TDP-43 (pTDP-43, *green*) levels in MNs expressing V5-tagged WT or mutant PFN1 (*red*). Insets show significant coaggregation between pTDP-43 and mutant PFN1. Quantification of the percentage of cells showing positive pTDP-43 signal is shown. Bars are mean±SEM; paired one-way ANOVA; **p*<0.05, ***p*<0.01. **E.** Increased insolubility of endogenous TDP-43 was confirmed using a detergent-based cellular fractionation in COS7 cells expressing WT or mutant PFN1. Triton X-100 (2%) and urea (8M) were used to extract the soluble and insoluble fraction, respectively. The quantification of the ratio of insoluble to soluble TDP-43 levels is shown. Values are normalized to untransfected cells (Untr., *red dashed line*). Bars are mean±SEM; one-way ANOVA with Dunnett's *post hoc* test; ***p*<0.01, ****p*<0.001. **F.** Representative blot of a co-immunoprecipitation (co-IP) assay between V5-tagged WT or mutant PFN1 and GFP-tagged TDP-43. A specific band corresponding to GFP-TDP-43 was detected in the IP pellet of both mutant PFN1, suggesting enhanced interaction of the soluble proteins. Scale bars: 10µm. DAPI (*blue*) was used to detect the cell nucleus and assess cell health.

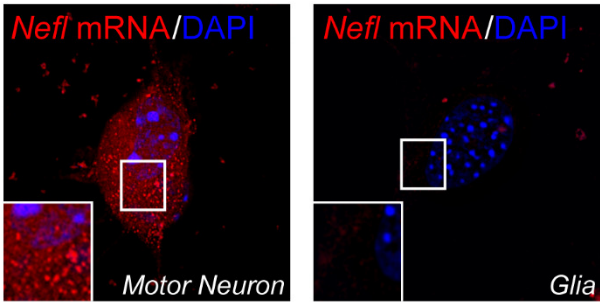

Supplementary Figure 10. Control experiments for TDP-43 dependent mRNA regulation. *Related to Figure 5.* *Nefl* mRNA FISH probes were used to hybridize neurons (*left panel*) or glia (*right panel*). As expected, no signal is present in glia cells, confirming the probes specificity. Insets show enlarged section of the cell body to indicate presence or absence of mRNA puncta. DAPI (*blue*) was used to detect the cell nucleus and assess cell health.

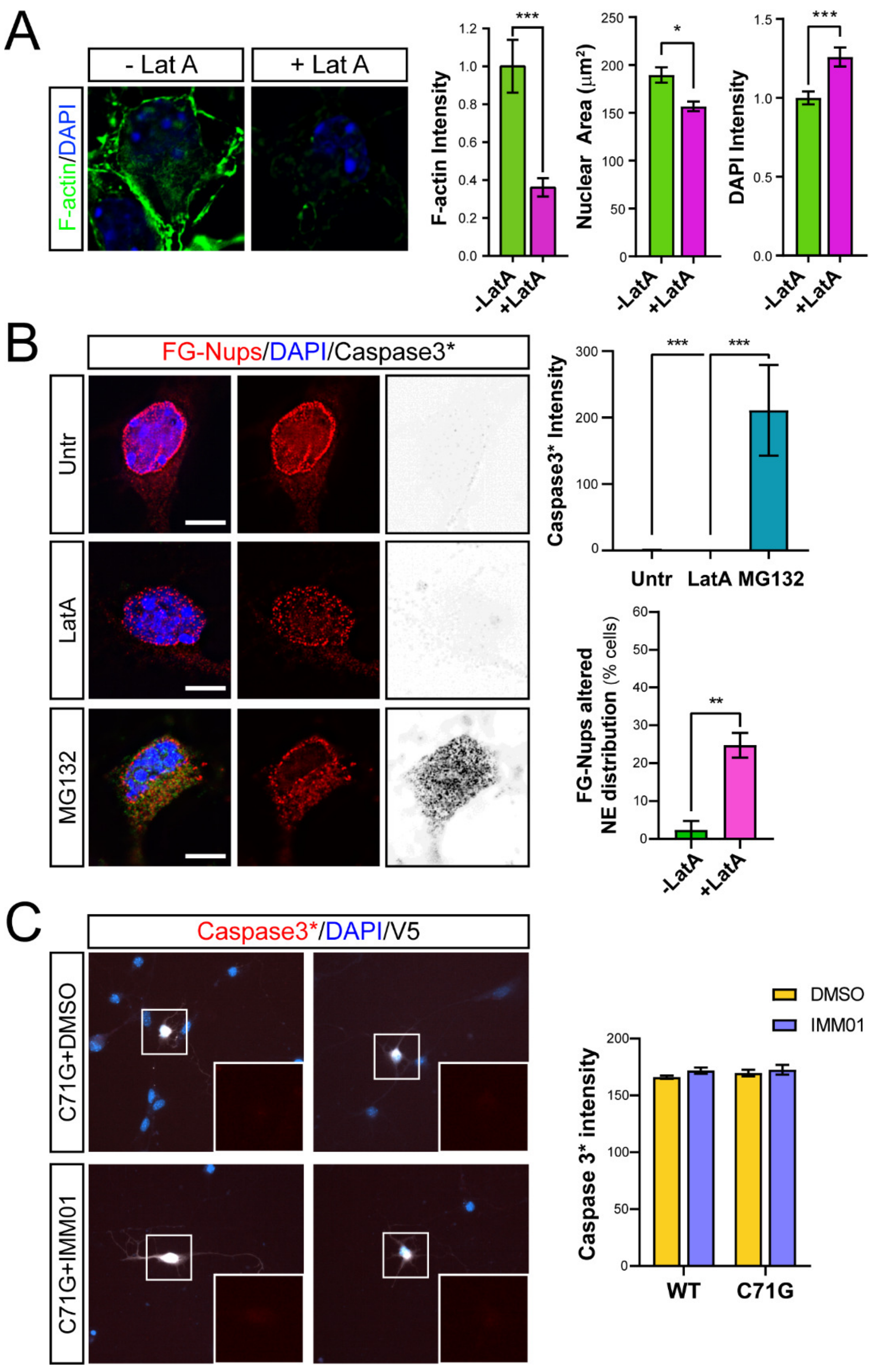

Supplementary Figure 11. Characterization of motor neurons following actin modulation. *Related to Figure 7.*
A. Treatment with 0.1µg/ml Latrunculin A (Lat A) for 3 days reduces cellular levels of F-actin in MNs, leading to smaller and denser nuclei. Phalloidin (*green*) was used to stain and quantify the remaining levels of filamentous F-actin. DAPI (*blue*) labeled the nuclear DNA. Bars are mean ± SEM. Student's *t* test; *$p<0.05$, ***$p<0.001$; n=43 and 51 for - and +LatA, respectively.
B. LatA treatment does not lead to increased apoptosis as detected by active Caspase 3 (Caspase3*) but increases the percentage of cells with abnormal distribution of the FG-Nups. MG132 (1µM for 24 hrs.) was used as positive control. F-actin. Bars are mean ± SEM. One way-ANOVA and Tukey's post hoc test; ***$p<0.001$. N= 36, 38, and 16 cells respectively from 3 independent experiments. Scale bars: 10µm.
C. Treatment of MNs with 0.1µM IMM01 for 24hrs does not lead to increased Caspase3 activation, indicative of apoptosis. V5-PFN1 positive MNs (*white*) show no staining for Caspase3* (*red*). DAPI (*blue*) indicates the nucleus. Insets are magnifications of the area indicated by the white box for Caspase3* staining.

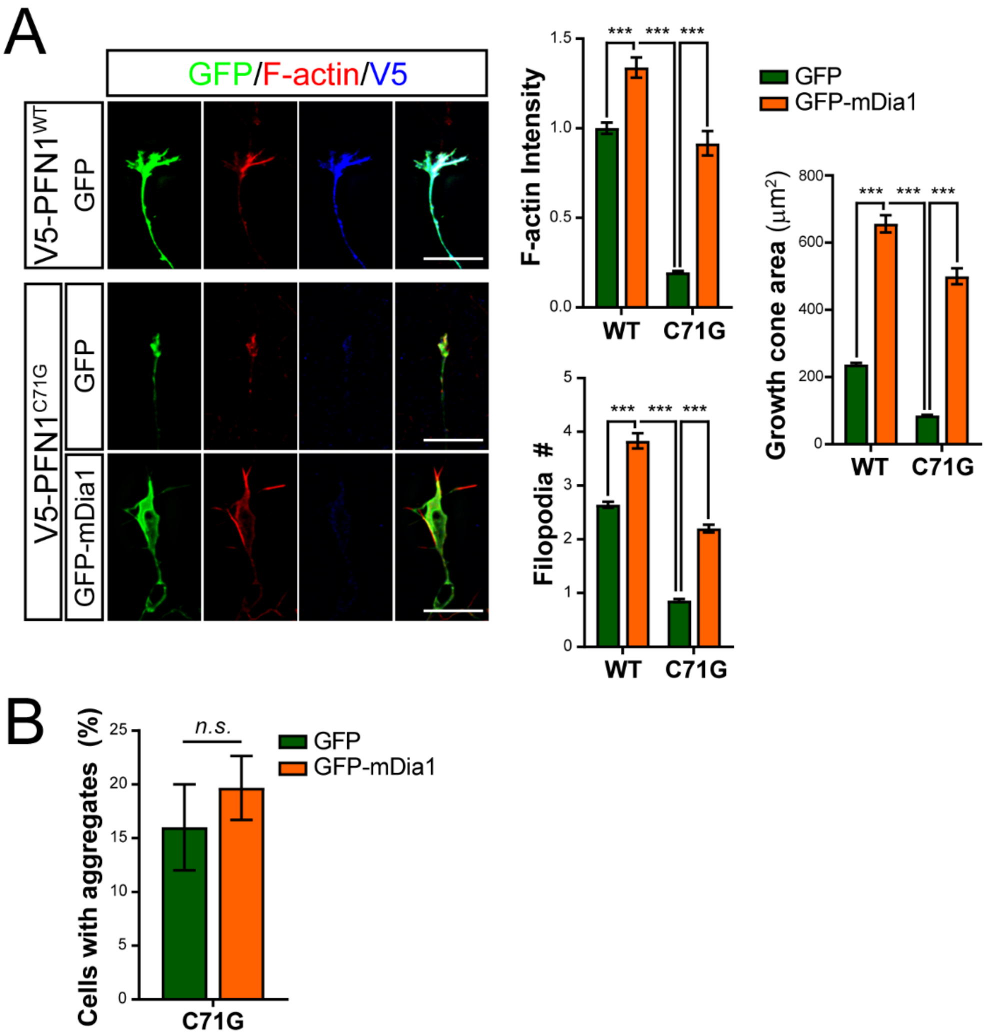

Supplementary Figure 12. mDia1 overexpression rescues actin polymerization in mutant PFN1 MNs. *Related to Figure 7*.

A. Overexpression of the constitutively active form of mDia1 rescues F-actin levels and actin-dependent morphological defects in the growth cone of mutant PFN1 MNs. F-actin (*red*) levels were quantified in the growth cones of WT or C71G-expressing MNs (*blue*). While $PFN1^{C71G}$ caused reduced F-actin levels compared to WT cells, leading to smaller growth cone with fewer filopodia, GFP-mDia1 expression restored normal F-actin polymerization and growth cone morphology. Two way-ANOVA; ****p*<0.001; n=30 cells for all conditions.

B. Overexpression of constitutively active mDia1 does not alter the frequency of $PFN1^{C71G}$ aggregate-containing cells. Paired *t* test; *n.s.*: non-significant; n=3. Scale bars: 5μm.

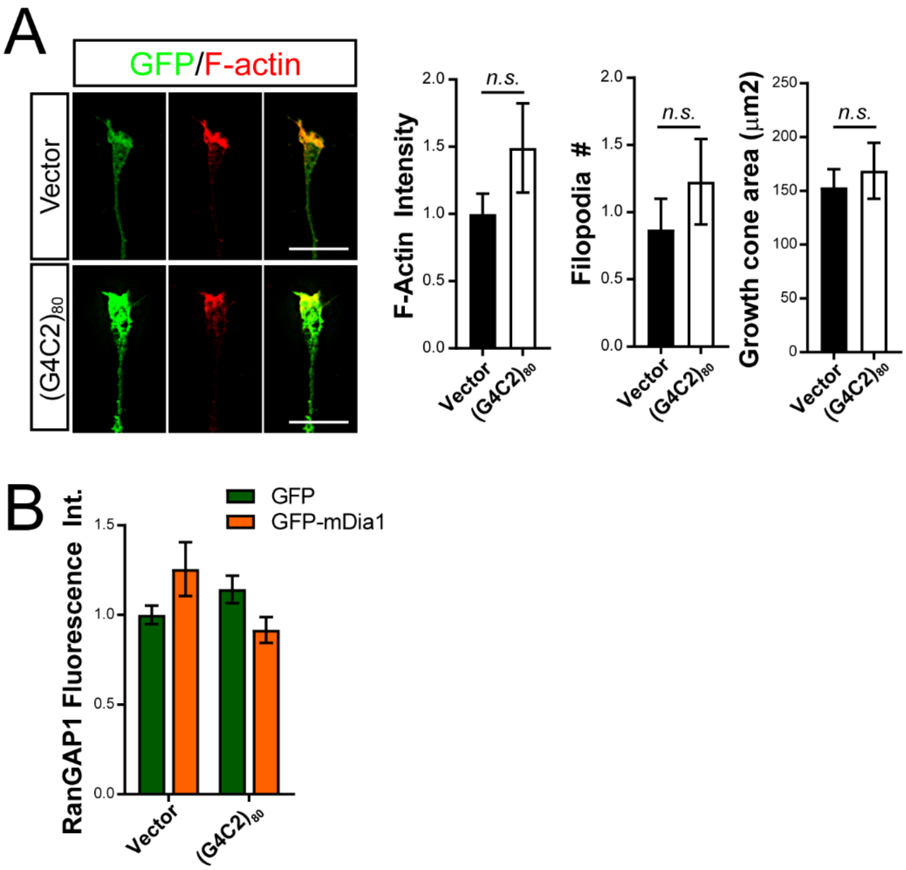

Supplementary Figure 13. Expression of $(G4C2)_{80}$ does not alter F-actin levels at growth cones. *Related to Figure 9*.

A. Expression of the *C9ORF72* repeat-expansion $(G4C2)_{80}$ does not affect F-actin levels and growth cone morphology. Student's *t* test with Welch correction; *n.s.*: non-significant; n= 23 and 31 cells for Vector and $(G4C2)_{80}$, respectively. **B.** Overall levels of RanGAP1 are unchanged in MNs expressing $(G4C2)_{80}$ repeat expansions in the presence or absence of GFP-mDia1. In all, bars are mean±SEM; scale bars: 10μm.

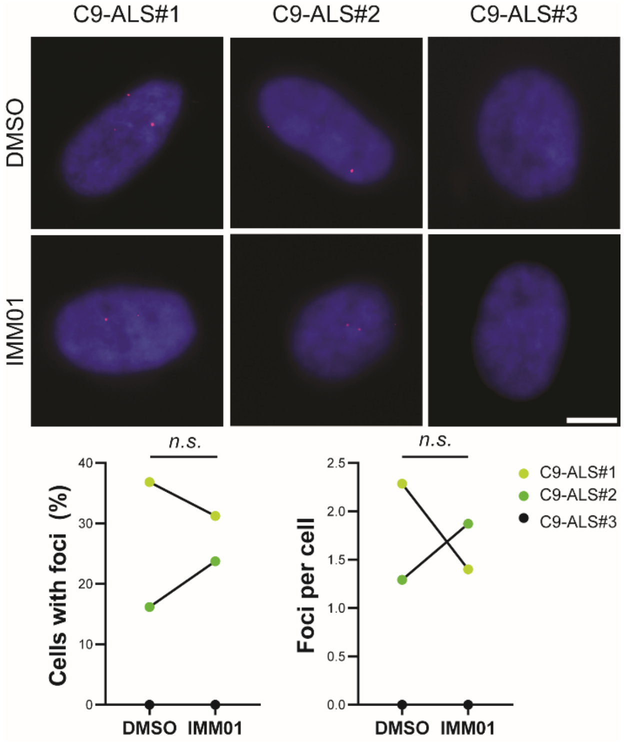

Supplementary Figure 14. IMM01 does not alter foci formation in C9-ALS fibroblasts. *Related to Figure 9.* Fibroblasts from 3 ALS patients were treated with 0.1µM IMM01 for 24 hr. and the percentage of cells with foci and the number of foci per cell were evaluated via FISH (*red*). No significant change was observed following treatment compared to DMSO control. DAPI (*blue*) was used to identify the cell nucleus. Paired t test, *n.s.* non-significant. Scale bar: 5µm.

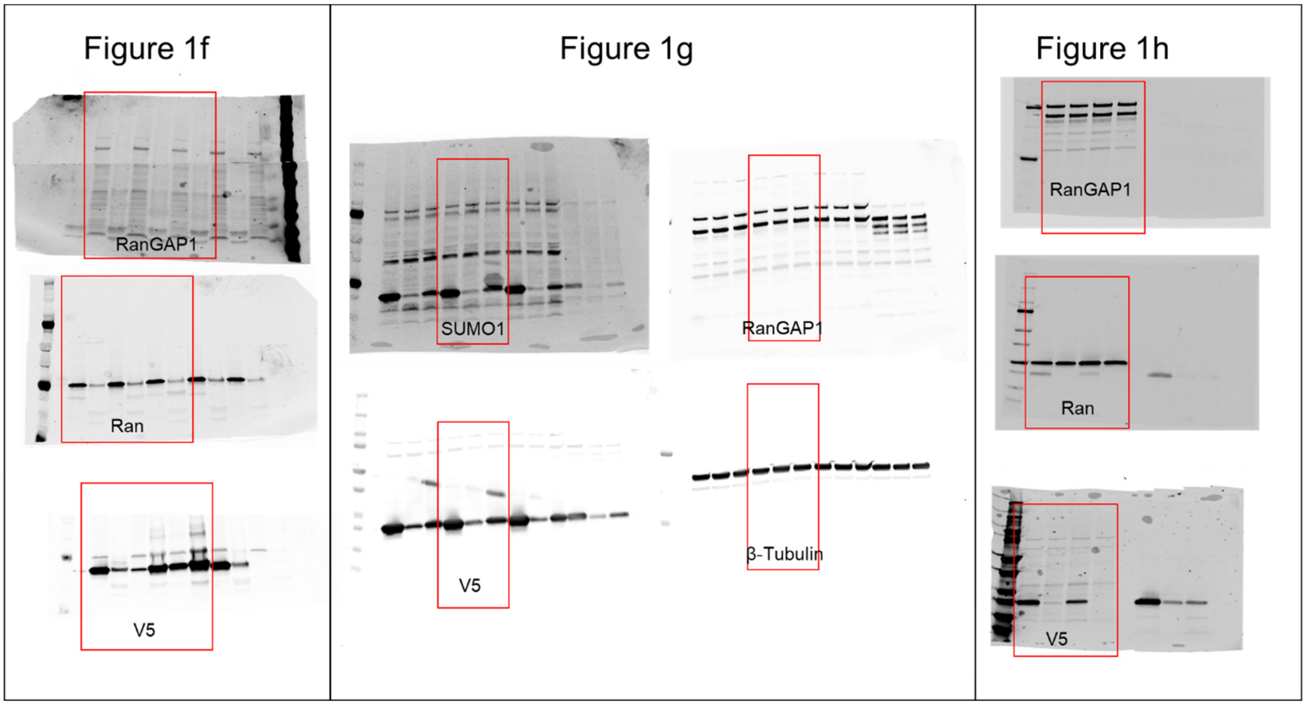

Supplementary Figure 15. Original uncropped scans for western blot assays. *Related to Figure 1.* Red boxes indicate the lanes shown in the cropped scans present in Figures 1f-h. The antibody used for hybridization is indicated for each blot.

	N	Mean±SEM	Statistical Test	Post *hoc* test	Confidence Interval; Degree of Freedom
**Figure 1**					
**1A**	WT: 3*<br>C71G: 3*<br>G118V: 3*	16.99%±4.99%<br>53.38%±2.38%<br>47.22%±6.41%	paired one way-ANOVA	Dunnett	WT vs.<br>C71G: -50.32 to -20.46; 2<br>G118V: -39.57 to -20.91; 2
**1B**	WT: 4*<br>C71G: 4*<br>G118V: 4*	22.53%±6.81%<br>68.71%±9.53%<br>49.26%±9.61%	paired one way-ANOVA	Dunnett	WT vs.<br>C71G: -63.99 to -28.36; 3<br>G118V: -52.37 to -1.075; 3
**1C**	WT: 5*<br>C71G: 5*<br>G118V: 5*	17.38%±5.81%<br>46.03%±5.83%<br>47.14%±7.34%	paired one way-ANOVA	Dunnett	WT vs.<br>C71G: 1.43 to 25.94; 4<br>G118V: 2.57 to 31.49; 4
**1D**	WT: 103<br>C71G: 85<br>G118V: 76	1.00±0.092<br>3.62±0.647<br>2.63±0.458	Kruskal-Wallis	Dunn	WT vs.<br>C71G: -3.94 to -1.30; 261<br>G118V: -2.99 to -0.27; 261
	WT: 103<br>C71G agg+: 22<br>C71G agg-: 63	1. 00±0.092<br>4.55±1.111<br>3.29±0.782	Kruskal-Wallis	Dunn	WT vs.<br>C71G: -5.89 to -1.22; 257<br>G118V: -3.89 to -0.70; 257
**Figure 2**					
**2B**	WT: 5*<br>C71G: 5*<br>G118V: 5*	24.85%±2.42%<br>52.21%±5.50 %<br>37.62%±3.94%	paired one way-ANOVA	Dunnett	WT vs.<br>C71G: -44.37 to -10.36; 4<br>G118V: -24.94 to -0.59; 4
**2C**	WT_normal: 44<br>WT_abnormal: 14<br>C71G_normal: 28<br>C71G_abnormal: 27<br>G118V_normal: 47<br>G118V_abnormal: 16	1.17±0.16<br>0.47±0.15<br>1.28±0.31<br>0.64±0.13<br>1.77±0.22<br>0.54±0.31	two way-ANOVA	Sidak	Normal - Abnormal"<br>WT: -0.20 to 1.60; 170<br>C71G: -0.16 to 1.43; 170<br>G118V: 0.38 to 2.09; 170
**Figure 3**					
**3Aa**	Ctrl_9512: 4*<br>Ctrl_7355:4*<br>Ctrl_9105:4*<br>6894_G118V: 4*<br>21001_C71G: 4*<br>9412_C71G: 4*	17.94%±2.06%<br>17.50%±6.29%<br>23.33%±6.94%<br>39.48%±6.00%<br>34.30%±7.07%<br>27.42%±5.12%	one way-ANOVA	Dunnett	Ctrls vs.<br>G118V_6894: -0.3667 to -0.03116; 20<br>C71G 9412: -0.3149 to 0.02069; 20<br>C71G_21001: -0.2461 to 0.08944; 20
**3Bb**	Ctrl_9512: 45<br>Ctrl_7355: 46<br>Ctrl_9105:48<br>6894_G118V: 45<br>21001_C71G: 47<br>9412_C71G: 47	1.01±0.10<br>0.89±0.08<br>1.08±0.15<br>1.34±0.28<br>1.55±0.19<br>1.34±0.12	one way-ANOVA	Dunnett	Ctrls vs.<br>G118V_6894: -0.858 to -0.00184; 271<br>C71G 9412: -1.055 to -0.2121; 271<br>C71G_21001: -0.845 to -0.0018; 271
**3Cc**	Ctrl_9512: 4*<br>Ctrl_7355:4*<br>Ctrl_9105:4*<br>6894_G118V: 4*<br>21001_C71G: 4*<br>9412_C71G: 4*	25.19%±5.98%<br>12.87%±4.70%<br>13.31%±3.95%<br>47.57%±6.06%<br>37.80%±7.02%<br>37.98%±7.00%	one way-ANOVA	Dunnett	Ctrls vs.<br>G118V_6894: -0.4837 to -0.1252; 20<br>C71G 9412: -0.3860 to -0.02750; 20<br>C71G_21001: -0.3878 to -0.02928; 20
**3Cc'**	Ctrl_9512: 4*<br>Ctrl_7355:4*<br>Ctrl_9105:4*<br>6894_G118V: 4*<br>21001_C71G: 4*<br>9412_C71G: 4*	21.47%±5.06%<br>16.27%±3.33%<br>9.86%±3.52%<br>30.69%±4.44%<br>43.23%±5.88%<br>35.06%±3.31%	one way-ANOVA	Dunnett	Ctrls vs.<br>G118V_6894: -0.2833 to -0.01314; 20<br>C71G 9412: -0.4087 to -0.1386; 20<br>C71G_21001: -0.3270 to -0.05687; 20

**Figure 4**					
**4B-C**	GFP: 44<br>WT: 44<br>C71G: 44	0.045±0.006<br>0.028±0.004<br>0.015±0.002	Kruskal-Wallis	Dunn	
**4D**	GFP: 4*<br>WT: 4*<br>C71G: 4*	0%±0%<br>5.74%±2.91%<br>39.04%±8.50%	one way-ANOVA	Tukey	GFPvWT: -30.32/18.85; 8<br>GFPvCG: -61.8/-16.28; 8<br>WTvCG: -57.89/-8.716; 8
**4E**	GFP: 22<br>WT: 37<br>C71G: 31	1±0.071<br>1.04±0.063<br>0.95±0.074	one way-ANOVA	N/A	GFPvWT: -0.29/0.20; 87<br>GFPvCG: -0.21/ 0.30; 87<br>WTvCG: -0.13/ 0.31; 87
**Figure 5**					
**5A**	WT: 42<br>C71G: 41<br>G118V: 34	1.00±0.192<br>3.56±1.262<br>2.32±0.668	Kruskal-Wallis	Dunn	
**5B**	WT: 45<br>C71G: 47<br>G118V: 44	1.00±0.209<br>4.07±1.142<br>3.07±0.901	Kruskal-Wallis	Dunn	
**5C** (Soma)	GFP: 40<br>WT: 29<br>C71G: 51	1.00±0.145<br>1.49±0.275<br>1.26±0.192	Kruskal-Wallis	Dunn	
**5C** (Axon)	GFP: 40<br>WT: 36<br>C71G: 53	1.00±0.096<br>0.91±0.097<br>0.64±0.063	Kruskal-Wallis	Dunn	
**5D**	Ctrls: 4*<br>6894_G118V: 4*<br>21001_C71G: 4*<br>9412_C71G: 4*	1.00±0.039<br>1.39±0.057<br>1.21±0.090<br>1.17±0.067	one way-ANOVA	Holm-Sidak	Mean Diff.±SE<br>Ctrl v 6894: -0.39±0.08<br>Ctrl v 21001: -0.21±0.08<br>Ctrl v 9412: -0.17±0.08
**Figure 6**					
**6A**	WT_DMSO:43<br>WT_KPT-276:40<br>C71G_DMSO:46<br>C71G_KPT-276:44	1.00±0.187<br>0.41±0.123<br>1.99±0.358<br>0.87±0.249	two way-ANOVA	Sidak	WT:D v CG:D: -2.5/-0.21; 168<br>WT:D v CG:K: -1.20/1.12; 168<br>CG:D v CG:K: 0.19/2.46; 168
**6B**	WT_DMSO:107<br>C71G_DMSO: 92<br>WT_KPT-276: 71<br>C71G_KPT-276:67	1.00±0.057<br>0.74±0.053<br>1.19±0.082<br>1.14±0.103	two way-ANOVA	Sidak	WT:D v CG:D: 0.01/0.50; 333<br>WT:D v CG:K: -0.41/0.13; 333<br>CG:D v CG:K: -0.67/-0.12; 333
**6C**	GFP_DMSO: 178<br>WT_DMSO: 207<br>C71G_DMSO: 164<br>GFP_KPT-276: 116<br>WT_KPT-276: 143<br>C71G_KPT-276: 158	1.00±0.058<br>1.04±0.048<br>0.67±0.054<br>0.80±0.063<br>0.83±0.063<br>1.04±0.066	two way-ANOVA	Sidak	GFP:D v WT:D:-0.25/0.18; 961<br>GFP:D v C71G:D: 0.11/0.56; 961<br>GFP:D v C71G:K:-0.27/0.19; 961<br>WT:D v C71G:D: 0.15/0.59; 961<br>WT:D v C71G:K: -0.22/0.22; 961<br>C71G:D v C71G:K: -0.61/-0.14; 961
**Figure 7**					
**7A**	-LatA: 4*<br>+LatA: 4*	18.94%±6.690%<br>46.77%±6.690%	Student's *t* test	N/A	13.87 to 41.78; 3
**7B**	-LatA: 45<br>+LatA: 50	1.00± 0.185<br>4.19±0.868	Student's *t* test	N/A	1.342 to 5.042; 93
**7C**	WT_GFP: 4*<br>C71G_GFP: 4*<br>WT_mDia1: 4*<br>C71G_mDia1: 4*	16.02%±9.814%<br>46.25%±5.708%<br>33.60%±2.290%<br>16.60%±6.273%	two way-ANOVA	Sidak	WT:GFP v WT:mDia: -0.45/0.10<br>WT:GFP v CG:GFP: -0.58/-0.03; 12<br>WT:GFP v CG:mDia: -0.28/0.27; 12<br>CG:GFP v CG:mDia: 0.02/0.57; 12

**7D**	WT_GFP: 73<br>C71G_GFP: 76<br>WT_mDia1: 67<br>C71G_mDia1: 61	1.00±0.098<br>1.54±0.232<br>0.933±0.133<br>1.00±0.112	two way-ANOVA	Tukey	WT:GFP v WT:mDia1: -0.42/0.57; 273<br>WT:GFP v CG:GFP: -1.01/-0.03; 273<br>WT:GFP v CG:mDia1: -0.51/ 0.47; 273<br>CG:GFP v CG: mDia1: 0.01/0.99; 273
**7E**	WT_DMSO: 3*<br>C71G_DMSO: 3*<br>WT_IMM01: 3*<br>C71G_IMM01: 3*	19.17%±3.63%<br>42.87%±5.86%<br>22.5%±2.04%<br>16.94%±4.03%	two way-ANOVA	Tukey	WT:D v WT:I: -26.05 to 19.38; 7<br>WT:D v C71G:D: -44.02 to -3.392; 7<br>WT:D v C71G:I: -18.09 to 22.54; 7<br>WT:I v C71G:D: -43.08 to 2.339; 7<br>WT:I v C71G:I: -17.16 to 28.27; 7<br>C71G:D v C71G:I: 5.614 to 46.24; 7
**7F**	WT_DMSO: 51<br>C71G_DMSO: 52<br>WT_IMM01: 31<br>C71G_IMM01: 51	1±0.24<br>2.83±0.51<br>1.54±0.40<br>1.26±0.23	two way-ANOVA	Tukey	WT:D v WT:I: -2.007 to 0.9274; 181<br>WT:D v C71G:D: -3.098 to -0.558; 181<br>WT:D v C71G:I: -1.534 to 1.018; 181<br>WT:I v C71G:D: -2.750 to 0.1737; 181<br>WT:I v C71G:I: -1.185 to 1.750; 181<br>C71G:D v C71G:I: 0.301 to 2.841; 181
**7G**	WT_DMSO: 50<br>C71G_DMSO: 60<br>WT_IMM01: 53<br>C71G_IMM01: 60	1.00±0.16<br>0.67±0.07<br>0.91±0.16<br>1.16±0.19	two way-ANOVA	Tukey	WT:D v WT:I: -0.4917 to 0.6710; 219<br>WT:D v C71G:D: -0.232 to 0.897; 219<br>WT:D v C71G:I: -0.726 to 0.4032; 219<br>WT:I v C71G:D: -0.313 to 0.7983; 219<br>WT:I v C71G:I: -0.8069 to 0.305; 219<br>C71G:D v C71G:I: -1.03 to 0.045; 219
**Figure 8**					
**8B-C**	WT_GFP: 47<br>C71G_GFP: 43<br>WT_mDia1: 50<br>C71G_mDia1: 44	0.077±0.002<br>0.043±0.001<br>0.058±0.001<br>0.063±0.002	two way-ANOVA	Sidak	WT:GFP v WT:mDia1: 0.01/0.02; 12<br>WT:GFP v CG:GFP: 0.03/0.04; 12<br>WT:GFP v CG:mDia1: 0.01/0.02; 12<br>CG:GFP v CG:mDia1: -0.03/-0.01; 12
**6D**	WT_GFP: 4*<br>C71G_GFP: 4*<br>WT_mDia1: 4*<br>C71G_mDia1: 4*	2.94±2.941<br>7.69±3.705<br>1.67±1.667<br>0±0	two way-ANOVA	N/A	WT:GFP v WT:mDia1: -9.25/11.8; 12<br>WT:GFP v CG:GFP: -15.28/5.78; 12<br>WT:GFP v CG:mDia1: -7.59/13.47; 12<br>CG:GFP v CG:mDia1: -2.84/18.22; 12
**Figure 9**					
**9A**	Vector_GFP:4*<br>Vector_mDia1:4*<br>$(G4C2)_{80}$_GFP:4*<br>$(G4C2)_{80}$_GFP:4*	13.91%±4.91%<br>23.30%±3.84%<br>35.84%±8.55%<br>18.39%±5.42%	two way-ANOVA	Sidak	Vec:GFP v Vec:mDia1: -4.58/23.36; 12<br>Vec:GFP v $(G4C2)_{80}$:GFP:7.96/35.91;12<br>Vec:GFP v $(G4C2)_{80}$:mDia1: -7.57/20.38; 12<br>$(G4C2)_{80}$:GFP v $(G4C2)_{80}$:mDia1: -29.5/-1.556; 12
**9B_ FG-nups**	Ctrl_DMSO:4*<br>C9-ALS_DMSO:4*<br>Ctrl_IMM01:4*<br>C9-ALS_DMSO:4*	25.96%±2.92%<br>47.82%±2.71%<br>23.62%±0.91%<br>36.80%±1.20%	two way-ANOVA	Tukey	Ctrls:D v. Ctrls:I: -6.598 to 11.28; 12<br>Ctrls:D v C9:D: -30.80 to -12.92; 12<br>Ctrls:D v C9:I: -19.77 to -1.900; 12<br>Ctrls:I v C9:D: -33.14 to -15.26; 12<br>Ctrls:I v C9:I: -22.11 to -4.239; 12<br>C9:D v C9:I: 2.088 to 19.96; 12
**9B_ Ran GAP1**	Ctrl_DMSO:5*<br>C9-ALS_DMSO:5*<br>Ctrl_IMM01:5*<br>C9-ALS_DMSO:5*	25.96%±%2.64<br>47.82%±3.08%<br>23.62%±%2.12<br>36.80%±1.95%	two way-ANOVA	Tukey	Ctrls:D v Ctrls:I: -8.781 to 11.35; 16<br>Ctrls:D v C9:D: -32.00 to -11.87; 16<br>Ctrls:D v C9:I: -21.00 to -0.8656; 16<br>Ctrls:I v C9:D: -33.29 to -13.15; 16<br>Ctrls:I v C9:I: -22.28 to -2.151; 16<br>C9:D v C9:I: 0.9363 to 21.07; 16

**9D-E**	Vector_GFP:64<br>Vector_mDia1:51<br>$(G4C2)_{80}$_GFP:52<br>$(G4C2)_{80}$_GFP:51	0.07±0.0005<br>0.04±0.0002<br>0.03±0.0005<br>0.05±0.0002	two way-ANOVA	Sidak	Vec:GFP v Vec:mDia1: 0.02/0.02; 14<br>Vec:GFP v $(G4C2)_{80}$:GFP: 0.03/0.03; 14<br>Vec:GFPv$(G4C2)_{80}$:mDia: 0.01/0.01; 14<br>$(G4C2)_{80}$:GFP v $(G4C2)_{80}$:mDia1:<br>-0.02 /-0.02; 14

Supplementary Table 1. Summary of experimental data and statistical analyses. *Related to Figures 1-9.*
N represents the number of cells collected from at least 3 independent experiments. If marked by an *, N equals the number of experiments.

REAGENT or RESOURCE	SOURCE	IDENTIFIER
Antibodies		
V5, mouse (1:10000)	Novus Bio NB100-62264	AB_965837
V5, rabbit (1:10000)	Novus Bio NB600-381	AB_10001084
FG-Nups (1:500)	Abcam Mab414	AB_24609
POM121 (1:100)	Protein Tech 15645-1-AP	N/A
TDP-43 (1:1000)	Protein Tech 12892-1-AP	AB_2200505
pTDP-43 (1:1000)	Protein Tech 22309-1-AP	AB_11182943
FUS (1:1000)	Bethyl Lab A300-293A	AB_263409
SMN (1:500)	BD 610646	AB_397973
FMRP (1:500)	Protein Tech 66548-1-Ig	N/A
Ran (1:500)	Bethyl Lab A304-297A	AB_2620493
RanGAP1 (1:200)	Everest BioTech EB06253	AB_2176966
RanGAP1 (1:500)	SantaCruz	sc-25630
Lamin A/C (1:500)	Sigma-Aldrich SAB4200236	AB_10743057
RanBP2 (1:100)	Bethyl Lab A301-796A	AB_1211503
SUMO1 (1:300)	Abgent AM1200a	AB_352490
β-tubulin (1:5000)	DSHB E7a	
GFP (1:5000)	Aves GFP-1020	AB_10000240
CRM1/XPO1 (1:200)	Bethyl Lab A300-469A	AB_451004
Importin-β (1:200)	Protein Tech 10077-1-AP	AB_2133977
Alexa Fluor 488 Phalloidin (1:500)	ThermoFisher A12379	AB_2315147
Alexa Fluor 546 Phalloidin (1:500)	ThermoFisher A22283	AB_2632953
Chemicals		
Latrunculin A	Cayman	10010630
DMSO	Sigma-Aldrich	D2650
KPT-276	Selleck	S7251
MG-132	Sigma-Aldrich	M7449
Leptomycin B	Enzo Life Science	ALX-380-100-C100
Intramimic-01	EMD Millipore	509583
Oligonucleotides		
POLDIP3_fwd	5'-gcttaatgccagaccgggagtt gga-3'	
POLDIP3_rev	5'-tcatcttcatccaggtcatataa att-3'	
GFP-PFN1_fwd	5'-ggtggctctggaggcggatccg ccgggtggaacgcctacatcg-3'	
GFP-PFN1_rev	5'-ttatctagatccggtggatcctcag Tactgggaacgccgaagg-3'	
GFP-mDia1$^{FH1-FH2}$_fwd	5'-gagactcgagccatggcttctctct ctgctg-3'	
GFP-mDia1$^{FH1-FH2}$_rev	5'-gagaggatccttagcttgcacg gccaac-3'	

Supplementary Table 2. List of critical reagents and resources. *Related to Figures 1-9.*

Probe #	Sequence	Probe #	Sequence
msNefl_1	gggggacctagagagaagaa	*msNefl_19*	ctaatgtctgcattctgctt
msNefl_2	cgtagccgaacgaactcatg	*msNefl_20*	tctccagtttgttgattgtg
msNefl_3	cgcttgtaggaggtcgaaaa	*msNefl_21*	catcttgacattgaggaggt
msNefl_4	agtagctggagtacgcggag	*msNefl_22*	ctgcaatctcgatgtccaag
msNefl_5	acggacagcgaggaggagac	*msNefl_23*	ccttccaagagttttctgta
msNefl_6	atcaaagagccagagctgga	*msNefl_24*	tgaaactgagcctggtctct
msNefl_7	ctcagatcgagattctccag	*msNefl_25*	aagacctgcgagctctgaga
msNefl_8	ggatagacttgaggtcgttg	*msNefl_26*	aagccactgtaagcagaacg
msNefl_9	atgaagctggcgaagcgatc	*msNefl_27*	gagcgagcagacatcaagta
msNefl_10	gaaggctcagagtgtttctg	*msNefl_28*	cagctttcgtagcctcaatg
msNefl_11	ttctcgttagtggcgtcttc	*msNefl_29*	ttgggaatagggctcaatct
msNefl_12	tcagcacttcttcctcatag	*msNefl_30*	attggggagaacttttcctg
msNefl_13	aaagctatctcgtccatcag	*msNefl_31*	tgtataggatctggaactca
msNefl_14	tctgagcatactggatctga	*msNefl_32*	cctaagtcatctcagaatta
msNefl_15	ttggaggacacgtccatctc	*msNefl_33*	tagcacaacattgaaagtcc
msNefl_16	ttgaaccactcttcggcgtt	*msNefl_34*	gatactctgcgtaaggagga
msNefl_17	tctcggttagcacggtgaag	*msNefl_35*	aaagccactctgcaagcaaa
msNefl_18	ttcgatctccagggtcttag	*msNefl_36*	ataagcatggaccatgcaca

Supplementary Table 3. Sequence information for the *Nefl* mRNA FISH probes. *Related to Figures 5 and 7.*
