# Supplementary material for: Modulation of actin polymerization affects nucleocytoplasmic transport in multiple forms of amyotrophic lateral sclerosis
Source: Nat Commun. 2019 Aug 23;10:3827. doi: 10.1038/s41467-019-11837-y (PMC6707192; doi:10.1038/s41467-019-11837-y)
Supplement: Supplementary file 3 — Reporting Summary [file 41467_2019_11837_MOESM3_ESM.pdf]

nature research

Corresponding author(s): Claudia Fallini, John Lander

Reporting Summary

Nature Research wishes to improve the reproducibility of the work that we publish. This form provides structure for consistency and transparency in reporting. For further information on Nature Research policies, see Authors & Referees and the Editorial Policy Checklist.

Statistical parameters

When statistical analyses are reported, confirm that the following items are present in the relevant location (e.g. figure legend, table legend, main text, or Methods section).

n/a	Confirmed	
☐	☒	The exact sample size (n) for each experimental group/condition, given as a discrete number and unit of measurement
☐	☒	An indication of whether measurements were taken from distinct samples or whether the same sample was measured repeatedly
☐	☒	The statistical test(s) used AND whether they are one- or two-sided<br>*Only common tests should be described solely by name; describe more complex techniques in the Methods section.*
☐	☒	A description of all covariates tested
☐	☒	A description of any assumptions or corrections, such as tests of normality and adjustment for multiple comparisons
☐	☒	A full description of the statistics including central tendency (e.g. means) or other basic estimates (e.g. regression coefficient) AND variation (e.g. standard deviation) or associated estimates of uncertainty (e.g. confidence intervals)
☐	☒	For null hypothesis testing, the test statistic (e.g. F, t, r) with confidence intervals, effect sizes, degrees of freedom and P value noted<br>*Give P values as exact values whenever suitable.*
☒	☐	For Bayesian analysis, information on the choice of priors and Markov chain Monte Carlo settings
☒	☐	For hierarchical and complex designs, identification of the appropriate level for tests and full reporting of outcomes
☒	☐	Estimates of effect sizes (e.g. Cohen's d, Pearson's r), indicating how they were calculated
☐	☒	Clearly defined error bars<br>*State explicitly what error bars represent (e.g. SD, SE, CI)*

Our web collection on statistics for biologists may be useful.

Software and code

Policy information about availability of computer code

Data collection	Nikon Elements for image acquisition; Autodeblur for image deconvolution
Data analysis	Fiji for image analysis

For manuscripts utilizing custom algorithms or software that are central to the research but not yet described in published literature, software must be made available to editors/reviewers upon request. We strongly encourage code deposition in a community repository (e.g. GitHub). See the Nature Research guidelines for submitting code & software for further information.

Data

Policy information about availability of data

All manuscripts must include a data availability statement. This statement should provide the following information, where applicable:

- Accession codes, unique identifiers, or web links for publicly available datasets
- A list of figures that have associated raw data
- A description of any restrictions on data availability

The datasets generated during the current study are available from the corresponding author on reasonable request.

Field-specific reporting

Please select the best fit for your research. If you are not sure, read the appropriate sections before making your selection.

☒ Life sciences ☐ Behavioural & social sciences ☐ Ecological, evolutionary & environmental sciences

For a reference copy of the document with all sections, see nature.com/authors/policies/ReportingSummary-flat.pdf

Life sciences study design

All studies must disclose on these points even when the disclosure is negative.

Sample size	for all experiments, a minimum of 10 cells were analyzed per conditions and the experiment was repeated at least 3 times. Thus, at least 30 cells per conditions were analyzed. We have employed this approach in the past for similar experimental set ups, and this sample size has been always sufficient to achieve statistical significance.
Data exclusions	no data were excluded from analysis
Replication	all experiments were replicated at least 3 times
Randomization	cells transfected and imaged with each set of constructs were selected randomly.
Blinding	analysis of protein localization to the NE was performed in blind

Reporting for specific materials, systems and methods

Materials & experimental systems

n/a	Involved in the study
☐	☒ Unique biological materials
☐	☒ Antibodies
☐	☒ Eukaryotic cell lines
☒	☐ Palaeontology
☒	☐ Animals and other organisms
☒	☐ Human research participants

Methods

n/a	Involved in the study
☒	☐ ChIP-seq
☒	☐ Flow cytometry
☒	☐ MRI-based neuroimaging

Unique biological materials

Policy information about availability of materials

Obtaining unique materials	patient derived lymphoblasts lines were obtained from Dr. Brown and previously published in Wu et al., 2011. The lines are available to researchers upon request. C9-ALS fibroblasts were obtained from Dr. Ratti and are available upon request

Antibodies

Antibodies used	a detailed description (name, Cat #, working concentration) is available in Supplementary Table 2
Validation	each antibody used was either validated by the manufacturer or in house by 1) optimizing working concentration, 2) testing in difference cell types, 3) comparing with other available antibodies

Eukaryotic cell lines

Policy information about cell lines

Cell line source(s)	Immortalized cell lines (HEK293 and N2a) were obtained from ATCC. Lymphoblast lines were obtained from Dr. Brown, fibroblasts were obtained from Dr. Ratti.
Authentication	lymphoblast lines and fibroblasts were subjected to sequencing to verify correct genotype. Fibroblasts were also subjected to FISH to confirm the presence of nuclear foci

Mycoplasma contamination	all lines were tested for mycoplasma contamination using Carnoy fixative method
Commonly misidentified lines<br>(See ICLAC register)	*Name any commonly misidentified cell lines used in the study and provide a rationale for their use.*
