# Supplementary material for: Modulation of actin polymerization affects nucleocytoplasmic transport in multiple forms of amyotrophic lateral sclerosis
Source: Nat Commun. 2019 Aug 23;10:3827. doi: 10.1038/s41467-019-11837-y (PMC6707192; doi:10.1038/s41467-019-11837-y)
Supplement: Supplementary file 4 — Description of Additional Supplementary Files [file 41467_2019_11837_MOESM4_ESM.docx]

**Description of Additional Supplementary Files**

File Name: Supplementary Movie 1
Description: Axonal outgrowth defects in KPT-267 treated and untreated PFN1-transfected motor neurons
