# Supplementary material for: Modulation of actin polymerization affects nucleocytoplasmic transport in multiple forms of amyotrophic lateral sclerosis
Source: Nat Commun. 2019 Aug 23;10:3827. doi: 10.1038/s41467-019-11837-y (PMC6707192; doi:10.1038/s41467-019-11837-y)
Supplement: Supplementary file 2 — Peer Review [file 41467_2019_11837_MOESM2_ESM.pdf]

Reviewers' comments:

Reviewer #1 (Remarks to the Author):

Giampetruzzi et al. explored an interesting connection between actin filament dynamics and NCT, two processes implicated in ALS, but the paper in its current version is problematic in both cell biology and translational studies.

A major weakness is that throughout the paper, the authors overexpressed PFN1, which is concerning because even overexpression of PFN1 WT causes defects. What is even more concerning is that their WT and mutant PFN1 levels, when overexpressed, are not comparable.
The authors need to genomically express mutant PFN1 to assess its toxicity (e.g. CRISP/Cas9).
Another major concern of this paper is whether the findings here are relevant to disease. Is there NCT defects in ALS patients with PFN1 mutants?
The authors should not call their defects pathology unless they show them in patient cells.
Also, can modulating actin filament dynamics suppresses NCT defects in published C9-ALS models?
The authors again overexpressed C9 repeats in cells to model C9-ALS and did not test whether mDia-1 affects the expression levels.
There are several C9-ALS iPS models that are not based on overexpressing system. Indeed, the authors concede that overexpressing C9 repeats in their cells did not result in any obvious defects in F-actin levels (Figure S10) that has been previously observed in primary MNs and patient derived cells and tissues harboring a G4C2 repeat expansion in the C9ORF72 locus (ref27). So the authors' model system used here is not suitable for their studies.

Reviewer #2 (Remarks to the Author):

Amyotrophic lateral sclerosis is a fatal motor neuron disease in which the majority of cases arise with no genetic component. However, the largest genetic cause of ALS is associated with a GGGGCC hexanucleotide repeat expansion in the first intron of the c9orf72 gene. Recent work has shown that nucleocytoplasmic transport deficits exist in C9ORF72 ALS. Nucleocytoplasmic transport impairment may also occur in other ALS subtypes. This is suggested by the cytoplasmic mislocalization of TDP43. The cause of protein mislocalization and nucleocytoplasmic transport is not fully understood. However, in this paper, Giampetruzzi and colleagues seek to identify a molecular mechanism by which nucleocytoplasmic transport impairment occurs in ALS.
These studies examine the impact of Profilin 1 (PFN1) mutations on nucleocytoplasmic transport. PFN1 regulates actin polymerization but can be mutated and aggregate in ALS. Therefore, this work seeks to examine whether PFN1 mutations cause nucleocytoplasmic transport impairment and the relationship between nucleocytoplasmic transport impairment and actin cytoskeleton integrity. These studies begin to adjust their hypotheses. However, the cohesiveness and fluidity of this work can certainly be improved upon.
Minor comments
1. Sample size should be indicated for each figure
2. Western blot experiments should include proper protein loading controls
3. Experiments focus on two PFN1 mutations. However, the prevalence and functional impact on these are not described in great detail. Please expand upon this information where appropriate.
4. Image quality should be improved upon.
5. Please describe the proper protein loading controls for soluble/insoluble fractionation as well as immunoprecipitation experiments presented.
6. Supplemental figure 2. Correct spelling of intensity.
7. Figure 6E is missing WT+mDia images
Major comments
1. The impact of PFN1 mutations on the NPC are described in figure 1. The text mentions the changes

in the NPC proteins in reference to nuclear envelope staining. However, nuclear envelope staining such as LaminB/B1 are presented. Please include also include nuclear envelope stain as point of reference. Further, the authors assess FG Nups. Which antibody was used to evaluate FG Nups? Please clarify which FG Nups are recognized by the chosen antibody and reference in the text.
2. Disruption of NPC in mutant PFN1 expressing cells is apparent. Is it possible that the expressing of PFN1 mutations are toxicity to the motor neurons? Please describe whether PFN1 mutations are toxic and if toxicity is occurring at timepoints in which NPC deficits are occurring.
3. Figure 1E discusses the formation of inclusions. Please clarify whether these are PFN1 inclusions.
4. Figure 1 shows altered NE distribution of various nucleocytoplasmic transport components with the expression of mutant PFN1. However, nuclear levels and whole cell levels are unchanged. Please describe whether changes in cytoplasm are also observed.
5. Karyopherins are essential for the trafficking of large proteins and RNA across the NPC. However, no change in importin beta was observed and a small reduction in XPO1 is presented in Figure S4. If nucleocytoplasmic transport is impaired with PFN1 one might expect karyopherin nuclear/cytoplasmic gradients to be disrupted. Please assess other the distribution and whole cell levels of other karyopherins.
6. Figure 2 nicely describes alterations in the nuclear envelope of Neuro2A cells expressing mutant PFN1 (C71G). Does this disruption occur with the other PFN1 mutation (G118V) relative to WT expressing cells? Furthermore, are these disruptions also observed in primary or iPSC neurons?
7. Figure 2 legend describes a slight reduction in overall lamin a/c levels in abnormal cells. However, nuclear intensity is not changed in abnormal cells relative to wild type expressing cells. Please elaborate on what this data may represent.
8. Nuclear import of molecules is facilitated by karyopherins and those karyopherins recognize a specific amino acid sequence. Depending upon the recognition site, this determines whether classical or nonclassical transport occurs. The authors show that transport rates are slowed in C71G PFN1 expressing cells (Figure 3). By determining the karyopherins responsible for trafficking the S-mCherry protein, this may aid in determining which karyopherins may show mislocalization in the PFN1 mutant expressing cells.
9. In order to compliment nuclear import impairment, the authors should show disruption of NPC selectivity barrier by dextran exclusion assay (Grote 2006 Nat. Protoc. 1 3034-3040 and Zhu 2016 J Mol Cell Biol 8(1),2-16). The authors should also assess nuclear export rates and validate that KPT-276 restores trafficking rates associated with the data collected in Figure 5.
10. The authors sought to connect disruption between nucleocytoplasmic transports and actin polymerization. In order to assess the role of actin polymerization on nucleocytoplasmic transport, the actin depolymerizing drug Latrunculin A (LatA) was used. Figure 6 shows that LatA disrupts RanGAP and Ran. It is important that the authors assess whether the cells are undergoing cell death at this time point. The relative Dapi Intensity appears to be increased in the presence of LatA and may be an indicator of cell death. Furthermore, the authors are seeking to determine whether modulating actin polymerization alters NPC defects. The authors should also assess whether nucleoporins are restored by mDia1.
11. GGGGCC-80 repeats did not show altered F-actin levels. However, mDia1 was able to rescue nuclear transport deficits. Is mDia1 altering the number of NPCs present. The authors should assess whether mDia1 alters nuclear pore complex components such as FG Nups and Pom121.

Reviewer #3 (Remarks to the Author):

This article provides convincing data for the potential of ALS-associated profilin 1 mutants to induce changes in nuclear envelope integrity, mislocalisation of proteins involved in nucleocytoplasmic transport and key RNA-binding proteins in ALS, and dysfunctional transport. The mutants are already known to reduce the promotion potential of actin polymerisation by profilin. A constitutively active formin (which can promote actin polymerisation independently of profilin) can rescue many of these defects from mutant profilin 1, as well as from overexpression of a C9orf72 repeat expansion.

Nucleocytoplasmic transport has gained attention as a key pathway in ALS pathogenesis, and this study gives mechanistic insight into pathways that feed into this common mechanism.
This study provides additional insight into pathomechanisms of neurodegeneration at the nuclear envelope, along with other recent studies. One of these, regarding effect of tau is not mentioned within the current manuscript probably as it was only recently published, but would be interesting for comparison as it regards another cytoskeletal protein (Eftekharzadeh et al., Neuron September 2018).

Overall, the data in this paper is of high quality and transparently presented, though requires improvement in places to satisfy conclusions drawn.

1. All results are caveated by the artefact of overexpression. The authors do not comment on this. Further efforts should be used to address this:
- Untransfected controls should be part of all analyses in the main manuscript – expression of WT protein does not 'seem' to have an impact on most results, but does effect the rate of nuclear import (figure 3B) – again, no comment on this from authors (also no N for this in table?).
- As patient PFN1 mutant cells are available to the authors (lymphoblasts, fig 4) and have a phenotype that the authors are concluding to be related to actin/nucleocytoplasmic transport, are the other key phenotypes observed in these cells?? (membrane integrity, Ran, RanGAP, nups, import…).

2. The authors also do not comment on the negative effects of actin polymerisation enhancement on WT neurons? (6F, 7A/C/D)

3. The authors claim "these results suggest that modulation of actin homeostasis directly modifies the stability of the NPC and/or NE, protecting it from disruption caused by ALS-associated gene mutations". No data is shown to support this, only that actin modulation (mDia) can rescue RanGAP1, TDP-43 and S-mCherry distribution, no nuclear envelope defects, no nuclear pore elements.

4. How is actin polymerisation rescuing these effects? How is actin/cytoskeleton at the nuclear envelope during changes and rescue?

5. The evidence for the involvement of mRNA post-transcriptional regulation is very limited, and no evidence for disruption via modulation of actin polymerisation is shown – as stated in abstract, neither is any link made to nucleocytoplasmic transport. Expression of mutant PFN1 reduces axonal neurofilament RNA, and one splicing event is changed in lymphoblasts from PFN1 mutant ALS lines (also why are controls pooled?). Does modulation of actin polymerisation/nuclear export rescue these changes?

6. Nuclear pore stability, structure and composition is frequently referred to, but only localisation of individual pore components is assessed.

7. Do the authors really consider that modulating actin – one of the main cytoskeletal components in all cells in the body - is a good therapeutic strategy?? Decreasing actin polymerisation is a target for metastasis in cancer.

Minor comments:
Some changes in clarity of description required.
- WT cells is ambiguous, these are not untransfected cells, but cells overexpression WT profilin1.
- Fig 1f,G require description
- state which proteins tested, not "no change in karyopherins", not all tested, p4
- role of NCT in toxicity "strongly" argues is a bit overstated, as no toxicity is tested/rescued in this study, discussion, p16
- what viscous cycle?, discussion, p16
No clear examples of profilin 1 forming aggregates are shown, some granular structures – is this what

the authors are referring to? What % of neurons have ‘aggregates’ versus diffuse, and is this related to the level of transfection/expression (total cell PFN level)?
Clarification of what is considered altered/abnormal/mislocalised should be given in the methods for each protein/structure assessed.
Table S1 – Both number of cells and number of experiments should be given. Please also explain statistical rationale for why number of experiments or cells is used as N in different experiments? Repeated measures/paired analyses can be carried out for non-parametric data using tests such as the Friedman test (if this is the reason).
No comment is given on frequent significant differences between mutants.
Why are some experiments done in cortical neurons?

RE: NCOMMS-18-28078

We thank the Reviewers for their insightful comments and helpful suggestions on how to improve our study by strengthening and supporting our conclusions with additional experimental evidence. Please find below a point-by-point response to the Reviewers' concerns. Changes in the manuscript are highlighted and marked by a side bar.

Reviewers' comments:

Reviewer #1 (Remarks to the Author):

Giampetruzzi et al. explored an interesting connection between actin filament dynamics and NCT, two processes implicated in ALS, but the paper in its current version is problematic in both cell biology and translational studies.

A major weakness is that throughout the paper, the authors overexpressed PFN1, which is concerning because even overexpression of PFN1 WT causes defects. What is even more concerning is that their WT and mutant PFN1 levels, when overexpressed, are not comparable. The authors need to genomically express mutant PFN1 to assess its toxicity (e.g. CRISP/Cas9).

We thank the Reviewer for raising such an important point. We have now performed an extensive analysis of nucleoporins and nucleoskeleton in lymphoblast cells derived from 3 ALS patient carrying either the C71G or G118V mutation. We found that endogenous expression of mutant PFN1 leads to changes in the localization and staining pattern of RanGAP1, Ran, and Lamin A/C, while FG-Nups - recognized by the mAb414 antibody (AbCam) – were significantly affected only in the G118V line, while both C71G mutant lines showed a small but non-significant change. Given that lymphoblast cells are actively dividing and thus refreshing their pool of nuclear pores at every cell division, it is not surprising to find slightly more subtle phenotypes in these cells compared to post-mitotic neurons. However, we cannot fully exclude a dose-dependent effect of mutant PFN1 expression on the mislocalization of these nucleoporins. Regardless, our new data support the hypothesis that mutant PFN1 expression causes alteration to the nuclear pore stability possibly leading to neuronal degeneration. We have included these new data in **Figure 3**.

We would also like to note that when transiently expressed in primary motor neurons, all V5-PFN1 constructs, regardless of the presence of the mutation, are expressed at similar levels. We have included a quantification of overall V5-PFN1 expression and subcellular localization (i.e. nucleus and cytoplasm) in supplementary **Supplementary Figure 1**. Although $PFN1^{C71G}$ is expressed at slightly lower levels compared to both WT and G118V mutants both in transfected motor neurons and lymphoblast cells, that difference does not reach statistical significance. No difference was observed between WT and G118V. We have included these data in **Supplementary Figure 1 and Supplementary Figure 6**.

Finally, we have performed an extensive analysis of the effect of $PFN1^{WT}$ overexpression on the localization of multiple nucleoporins and RNA-binding proteins, and we show no changes but for a minor effect on Lamin A/C staining (**Supplementary Figure 2**). We

believe together these data provide strong support to our hypothesis that mutant PFN1 affects the NPC rather than being an artifact of overexpression.

Another major concern of this paper is whether the findings here are relevant to disease. Is there NCT defects in ALS patients with PFN1 mutants?

Unfortunately, mutations in PFN1 are rare in the ALS population. Currently we have no access to patient brain tissue carrying PFN1 mutations, so this question is hard to address. However, our new data obtained from patient-derived lymphoblast cells showing disrupted nucleoporin and lamin staining (see **Figure 3**) support the hypothesis that the defects described here are relevant to the human disease as they are present in cells endogenously expressing mutant PFN1 compared to several control lines.

It is important to note that, although mutations in PFN1 are rare, changes to the cytoskeletal structure and function are prominent in both sporadic and familial ALS (1–7). Examining PFN1 mutants thus provides insights into the mechanisms by which alterations to the cytoskeleton can contribute to ALS. Furthermore, we would like to point out that defects to the NPC similar to what described here have been observed in many other forms of ALS for which post-mortem tissue is available, including C9ORF72, TDP-43, and sporadic ALS cases, as well as other neurodegenerative diseases (8–12). Our data show that modulating actin dynamics can attenuate NCT defects in neurons and patient-derived cells possessing the G4C2 repeat expansion in the C9orf72 locus. This stresses the importance of the findings we report for understanding ALS, not just ALS caused by PFN1 mutants.

The authors should not call their defects pathology unless they show them in patient cells.

We apologize for the lack of clarity. We have now rephrased this as “cellular defects”.

Also, can modulating actin filament dynamics suppresses NCT defects in published C9-ALS models?

On a similar note Reviewer#1 comments:

There are several C9-ALS iPS models that are not based on overexpressing system. Indeed, the authors concede that overexpressing C9 repeats in their cells did not result in any obvious defects in F-actin levels (Figure S10) that has been previously observed in primary MNs and patient derived cells and tissues harboring a G4C2 repeat expansion in the C9ORF72 locus (ref27). So the authors’ model system used here is not suitable for their studies.

In the study from Sivadasan et al., (2016) the authors show changes in F-actin levels following knock down of the C9ORF72 protein in primary motor neurons. However, only changes to actin dynamics - but not to F-actin overall levels - were reported in patient-derived cells (13). In our hands, primary motor neurons transfected with the G4C2 repeat expansion do not show obvious changes to F-actin levels at the growth cone. Since changes to the levels of the C9ORF72 protein are not expected under these conditions, we believe our data are not in conflict with the previous report.

Nonetheless, we agree with the reviewer that patient-derived cells carrying the G4C2 repeat expansion are a more suitable model to test the effect of cytoskeletal modulation on nucleocytoplasmic transport. As suggested by the reviewer, we have now performed

the analysis of nucleoporins FG-Nups (as detected by the mAb414 antibody) and RanGAP1 in primary fibroblasts derived from 3 C9-ALS patients and 3 controls. As shown in **Figure 9**, we find that a higher percentage of C9-ALS fibroblasts have disrupted or abnormal staining for both proteins, as previously reported (9,10,14). Treatment of these cells with IMM01, a selective activator of formins (15), leads to a significant rescue of both RanGAP1 and FG-Nups localization at the nuclear envelope. Together with our data in primary motor neurons and patient-derived lymphoblast lines, these results clearly show that positive or negative modulation of the actin cytoskeleton affects the nuclear pore structure and function in multiple models of ALS.

The authors again overexpressed C9 repeats in cells to model C9-ALS and did not test whether mDia-1 affects the expression levels.

We have now performed FISH assays to quantify the formation of C9ORF72-positive foci in C9-ALS fibroblasts in the presence or absence of IMM01. No change in the percentage of cells with fibroblasts or in the number of foci per cell was observed following IMM01 treatment. We have included this information in **Supplementary Figure 14**.

Reviewer #2 (Remarks to the Author):

Amyotrophic lateral sclerosis is a fatal motor neuron disease in which the majority of cases arise with no genetic component. However, the largest genetic cause of ALS is associated with a GGGGCC hexanucleotide repeat expansion in the first intron of the c9orf72 gene. Recent work has shown that nucleocytoplasmic transport deficits exist in C9ORF72 ALS. Nucleocytoplasmic transport impairment may also occur in other ALS subtypes. This is suggested by the cytoplasmic mislocalization of TDP43. The cause of protein mislocalization and nucleocytoplasmic transport is not fully understood. However, in this paper, Giampetruzzi and colleagues seek to identify a molecular mechanism by which nucleocytoplasmic transport impairment occurs in ALS.
These studies examine the impact of Profilin 1 (PFN1) mutations on nucleocytoplasmic transport. PFN1 regulates actin polymerization but can be mutated and aggregate in ALS. Therefore, this work seeks to examine whether PFN1 mutations cause nucleocytoplasmic transport impairment and the relationship between nucleocytoplasmic transport impairment and actin cytoskeleton integrity. These studies begin to adjust their hypotheses. However, the cohesiveness and fluidity of this work can certainly be improved upon.

Minor comments

1. Sample size should be indicated for each figure

We have now included that information in each figure legend in addition to more detailed information on statistical analysis presented in **Supplementary Table 1**.

2. Western blot experiments should include proper protein loading controls
Similarly, Reviewer #2 states:
5. Please describe the proper protein loading controls for soluble/insoluble fractionation as well as immunoprecipitation experiments presented.

For our western blots we have used β-tubulin as loading control (see Figure 1H). However, we are unaware of a loading control that could be used as a reference for solubility assays and co-IPs. The aim of the solubility assays was to evaluate the possible co-aggregation of different proteins such as Ran and RanGAP1 with mutant PFN1. As such, we have used PFN1 itself as a control for our assays as we and other have shown that mutations in PFN1 render the protein unstable and redistribute it to the insoluble fraction (16–20). Similarly, for co-IP assays we used PFN1 itself as our reference as previously done (17,19). We have repeated these assays multiple times and we are confident that the data presented here is solid and reproducible.

3. Experiments focus on two PFN1 mutations. However, the prevalence and functional impact on these are not described in great detail. Please expand upon this information where appropriate.

We apologize for the lack of information about PFN1 mutations. We have now included a short paragraph in the introduction describing the prevalence and effect of these mutations.

4. Image quality should be improved upon.

High resolution images have been uploaded through Nature Communications website.

6. Supplemental figure 2. Correct spelling of intensity.

This typo has been fixed.

7. Figure 6E is missing WT+mDia images

We have now included the missing images in Figure 6E.

Major comments

1. The impact of PFN1 mutations on the NPC are described in figure 1. The text mentions the changes in the NPC proteins in reference to nuclear envelope staining. However, nuclear envelope staining such as LaminB/B1 are presented. Please include also include nuclear envelope stain as point of reference.

For the analysis of nucleoporin localization, we have used DAPI staining as a reference for the nuclear membrane as commonly performed (8,9,11). Indeed, DAPI staining borders the Lamin A/C staining as shown in the image below. Thus, we consider DAPI to be a reliable marker for nuclear envelope. In addition, we find that Lamin A/C staining is disrupted in mutant PFN1 motor neurons, thus reducing its usefulness as a marker of the nuclear envelope. We have clarified this point in the Methods and Results sections (**pg. 4 and pg. 21**).

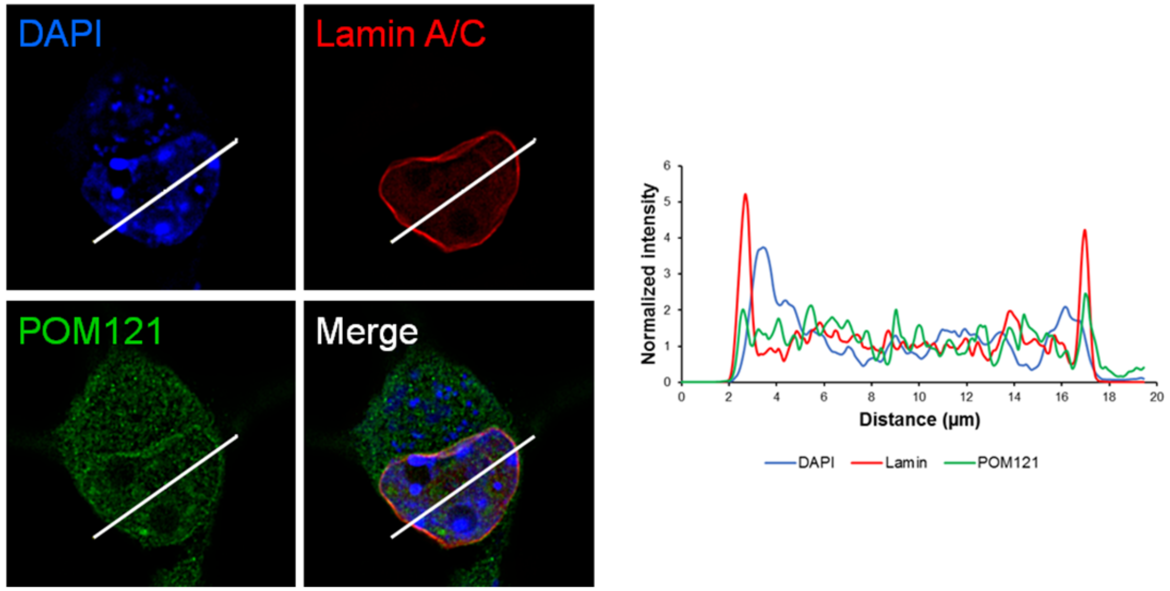

Figure 1. DAPI and Lamin A/C staining correlate with nucleoporin POM121 localization. Primary motor neurons were stained with Lamin A/C (red), POM121 (green), and DAPI (blue). Line plot shows the overlap between the signals.

Further, the authors assess FG Nups. Which antibody was used to evaluate FG Nups? Please clarify which FG Nups are recognized by the chosen antibody and reference in the text.

> We apologize for the oversight. We have fixed this by specifying both the antibody (i.e. mAb414, Abcam) and nucleoporins detected by it (i.e. Nup62, Nup90, and Nup52) in the Results section (**pg. 4**).

2. Disruption of NPC in mutant PFN1 expressing cells is apparent. Is it possible that the expressing of PFN1 mutations are toxicity to the motor neurons? Please describe whether PFN1 mutations are toxic and if toxicity is occurring at timepoints in which NPC deficits are occurring.

> We thank the reviewer for raising this important point. To address this potential issue, we have performed immunofluorescence analysis to quantify Caspase 3 activation in motor neurons expressing either WT or mutant PFN1, that would be indicative of the activation of the apoptotic cell death pathway. We did not detect any signs of apoptosis at the time tested, strongly suggesting that the effects on the NPC observed are not an epiphenomenon dependent on the degenerative process. We have included this data in **Supplementary Figure 1**.

3. Figure 1E discusses the formation of inclusions. Please clarify whether these are PFN1 inclusions.

> The aggregates shown in figure 1E are indeed $PFN1^{C71G}$-positive, as identified by V5 staining. We have indicated this in the text and figure legend (**pg. 5**).

4. Figure 1 shows altered NE distribution of various nucleocytoplasmic transport components with the expression of mutant PFN1. However, nuclear levels and whole cell levels are unchanged. Please describe whether changes in cytoplasm are also observed.

Our immunofluorescence analysis did not uncover any change to the overall levels of the Nups tested in the cell nucleus or cytoplasm. We have included this information in **Supplementary Figure 4**.

5. Karyopherins are essential for the trafficking of large proteins and RNA across the NPC. However, no change in importin beta was observed and a small reduction in XPO1 is presented in Figure S4. If nucleocytoplasmic transport is impaired with PFN1 one might expect karyopherin nuclear/cytoplasmic gradients to be disrupted. Please assess other the distribution and whole cell levels of other karyopherins.

On a similar note Reviewer #2 states:

8. Nuclear import of molecules is facilitated by karyopherins and those karyopherins recognize a specific amino acid sequence. Depending upon the recognition site, this determines whether classical or nonclassical transport occurs. The authors show that transport rates are slowed in C71G PFN1 expressing cells (Figure 3). By determining the karyopherins responsible for trafficking the S-mCherry protein, this may aid in determining which karyopherins may show mislocalization in the PFN1 mutant expressing cells.

The S-mCherry reporter we used was obtained through Addgene from the Di Ventura and Eils labs (Addgene # 72660). This reporter contains a synthetic NES sequence designed specifically to interact with XPO1, and a NLS sequence (AAKRVKLD) modeled upon c-Myc NLS, which is recognized by Importin α/β as described in (21). Indeed, our data show that XPO1 inhibitors block S-mCherry nuclear export (**Figure 4**). Thus, we believe XPO1 and Importin β are indeed the most relevant karyopherin to study in this context. We have analyzed the localization of a few other karyopherins such as XPO5 and importin α and found no change in their C:N ratios (not shown). Although we would be happy to include this data, we feel it would not provide additional insight into the PFN1-dependent defect of nuclear pore. It would be interesting to perform an in-depth analysis of all karyopherins' localization and function in the presence of mutant ALS proteins such as C9orf72 and PFN1, but we believe this goes beyond the scope of this study and will be the subject of future investigation.

6. Figure 2 nicely describes alterations in the nuclear envelope of Neuro2A cells expressing mutant PFN1 (C71G). Does this disruption occur with the other PFN1 mutation (G118V) relative to WT expressing cells? Furthermore, are these disruptions also observed in primary or iPSC neurons?

We performed EM analysis only on the $PFN1^{C71G}$ mutation compared to $PFN1^{WT}$ since this mutant showed the most severe disruption of nucleoporin staining in motor neurons. We have further confirmed these alterations to the nuclear envelope by immunostaining, using Lamin A/C as a specific marker. Similarly to what observed in N2a cells, motor neurons expressing both C71G and G118V mutants have irregular LaminA/C staining, supporting and expanding upon the observations obtained by EM. In addition, we have now performed the analysis of Lamin A/C staining in lymphoblasts lines derived from 3 ALS patients, and we find similar defects as observed in transiently transfected motor neurons (see **Figure 3**). Together, these data suggest that alterations to the nuclear envelope and Lamin A/C-positive nucleoskeleton are relevant and early defects caused by mutant PFN1 expression.

7. Figure 2 legend describes a slight reduction in overall lamin a/c levels in abnormal cells. However, nuclear intensity is not changed in abnormal cells relative to wild type expressing cells. Please elaborate on what this data may represent.

In our analysis of Lamin A/C expression levels, we found that cells with disrupted Lamin staining also had reduced levels of the protein. This might suggest that the mislocalized Lamin A/C could be targeted for degradation via the autophagy or proteosome systems. Interestingly, while PFN1 mutations increased the percentage of cells with abnormal Lamin A/C, the reduction in Lamin A/C levels was observed in all cells with abnormal staining, suggesting that this effect may not be a direct effect of PFN1 mutants but rather a consequence of the alteration in nuclear envelope stability. We have now included a better explanation of these data in the results section (**pg. 5**).

9. In order to compliment nuclear import impairment, the authors should show disruption of NPC selectivity barrier by dextran exclusion assay (Grote 2006 Nat. Protoc. 1 3034-3040 and Zhu 2016 J Mol Cell Biol 8(1),2-16).

We have now performed dextran exclusion assay in lymphoblasts lines derived from 3 ALS patients and 3 healthy controls. We found that the ALS lines are characterized by defects to the nuclear pore such as FG-Nups mislocalization, Ran cytoplasmic accumulation, and Lamin A/C alterations (see new **Figure 3**). However, no change in the membrane integrity has been observed, suggesting this is not an early event caused by mutant PFN1. However, as mentioned above, it is worth notice that lymphoblasts are dividing cells, so defects in these lines could be more subtle than postmitotic neurons, where the nuclear envelope is never dissolved and reassembled. We were unable to perform the same assay in these neurons for technical reasons, i.e. the incompatibility of the need to transfect, postfix and stain the neurons following the dextran assays. We have included these new data in **Supplementary Figure 6** and edited the manuscript accordingly.

The authors should also assess nuclear export rates and validate that KPT-276 restores trafficking rates associated with the data collected in Figure 5.

Our data suggest that nuclear export is not affected by mutant PFN1. Indeed, we see no changes in the nucleocytoplasmic distribution of mostly cytoplasmic proteins such as SMN, FMRP, PFN1, or the S-mCherry reporter itself. In addition, dextran exclusion assays revealed no alterations to the integrity of the nuclear membrane, excluding a generalized defect to the NCT. We have attempted to measure nuclear export rates via live cell imaging using the import inhibitor Importazole (Cayman) but since the S-mCherry reporter localizes very poorly to the nucleus, this makes it impossible to observe a cytoplasmic translocation.

As for the KPT-276 effect on export rates, we confirmed that the concentration used in our experiment (i.e. 50nM) was sufficient to modulate the cellular distribution of the S-mCherry reporter similarly to Leptomycin B (Figure 2). Indeed, both KPT276 and Leptomycin B have similar binding to XPO1 and a similar mechanism of action (22).

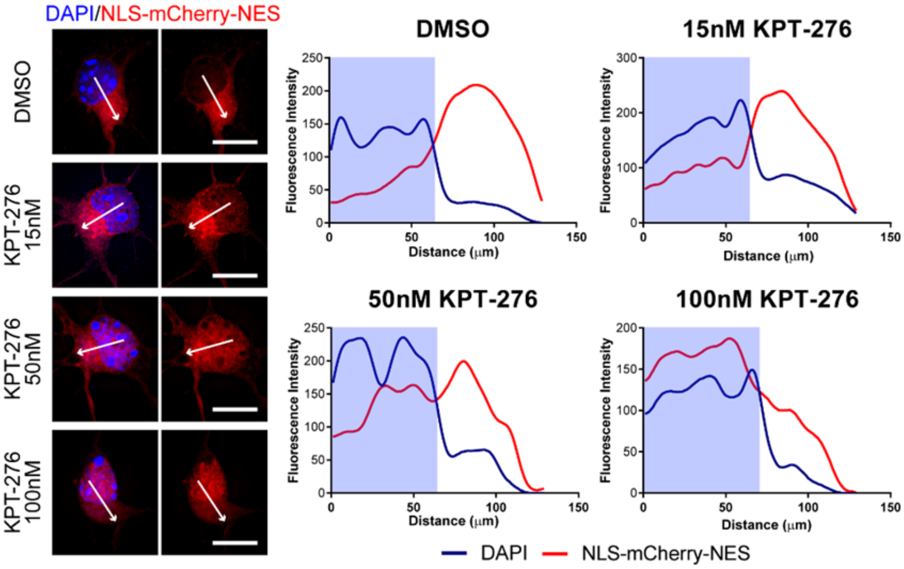

Figure 2. KPT-276 inhibits S-mCherry nuclear import. Representative images of MNs transfected with the nucleocytoplasmic reporter NLS-mCherry-NES (*red*) and treated with either DMSO or increasing concentrations of KPT-276. Line plots show the distribution of NLS-mCherry-NES (*red line*) relative to nuclear DAPI (*blue line*). Blue shaded rectangles delineate the nuclear volume. Scale bars: 10µm.

10. The authors sought to connect disruption between nucleocytoplasmic transports and actin polymerization. In order to assess the role of actin polymerization on nucleocytoplasmic transport, the actin depolymerizing drug Latrunculin A (LatA) was used. Figure 6 shows that LatA disrupts RanGAP and Ran. It is important that the authors assess whether the cells are undergoing cell death at this time point.

The relative Dapi Intensity appears to be increased in the presence of LatA and may be an indicator of cell death.

The Reviewer raises a very important point here. Indeed, in neurons treated with LatA, the DAPI mean fluorescence intensity is increased and the average nuclear area is decreased, suggesting that actin cytoskeleton in necessary to maintain proper nuclear morphology. However, we have not detected any signs of apoptosis, as defined by the presence of pyknotic nuclei or activation of Caspase 3, a classic indicator of apoptosis initiation. We have included these data in **Supplementary Figure 11**. Overall, these results suggest that nucleoporin mislocalization is directly linked to alterations to the actin network.

Furthermore, the authors are seeking to determine whether modulating actin polymerization alters NPC defects. The authors should also assess whether nucleoporins are restored by mDia1.

Similarly, Reviewer #2 comments:

11. GGGGCC-80 repeats did not show altered F-actin levels. However, mDia1 was able to rescue nuclear transport deficits. Is mDia1 altering the number of NPCs present. The authors should assess whether mDia1 alters nuclear pore complex components such as FG Nups and Pom121.

To further strengthen the causal link between actin modulation and nucleocytoplasmic transport, we have employed a new complementary pharmaceutical approach to modulate actin polymerization compared to the overexpression of constitutively active

mDia1. In order to enhance formin function, we treated primary motor neurons with the selective formin agonist Intramimic-01 (IMM01) (15). This small molecule prevents formin autoinhibition, thereby leading to a significant increase in its actin polymerization activity. Treatment of primary motor neurons with low concentrations of IMM01 (i.e. 0.1μM) for 24h led to the rescue of FG-Nups defective localization at the nuclear envelope, as well as of the Ran gradient and Neurofilament mRNA localization to the axon (see **Figure 7**). In primary fibroblasts derived from 3 ALS patients carrying G4C2 repeat expansions in *C9ORF72* gene, IMM01 was able to rescue RanGAP1 and FG-Nups localization as well (see **Figure 9**). Together, these data strongly support our conclusion that modulation of the actin cytoskeleton affect the structure and function of the nuclear pore in multiple cellular models of ALS.

Reviewer #3 (Remarks to the Author):

This article provides convincing data for the potential of ALS-associated profilin 1 mutants to induce changes in nuclear envelope integrity, mislocalisation of proteins involved in nucleocytoplasmic transport and key RNA-binding proteins in ALS, and dysfunctional transport. The mutants are already known to reduce the promotion potential of actin polymerisation by profilin. A constitutively active formin (which can promote actin polymerisation independently of profilin) can rescue many of these defects from mutant profilin 1, as well as from overexpression of a C9orf72 repeat expansion. Nucleocytoplasmic transport has gained attention as a key pathway in ALS pathogenesis, and this study gives mechanistic insight into pathways that feed into this common mechanism.

We would like to thank the Reviewer for their supportive comments of the significance of our study and the conclusive nature of our experiements.

This study provides additional insight into pathomechanisms of neurodegeneration at the nuclear envelope, along with other recent studies. One of these, regarding effect of tau is not mentioned within the current manuscript probably as it was only recently published, but would be interesting for comparison as it regards another cytoskeletal protein (Eftekharzadeh et al., Neuron September 2018).

The reviewer is correct that the paper in question was published after this manuscript was first submitted. We have included a discussion of this and few other new studies on **pg. 13**.

Overall, the data in this paper is of high quality and transparently presented, though requires improvement in places to satisfy conclusions drawn.

We again would like to thank the Reviewer for acknowledging the quality of our work and the mechanistic insight that our study adds to the field of neurodegeneration and nucleocytoplasmic transport research.

1. All results are caveated by the artefact of overexpression. The authors do not comment on this. Further efforts should be used to address this:
- Untransfected controls should be part of all analyses in the main manuscript – expression of WT protein does not 'seem' to have an impact on most results, but does effect the rate of

nuclear import (figure 3B) – again, no comment on this from authors (also no N for this in table?).

We have now performed an in-depth analysis of the effect of $PFN1^{WT}$ expression on the localization of all nucleoporin and RBPs tested. We found that $PFN1^{WT}$ expression does not affect the localization of all proteins but Lamin A/C (**Supplementary Figure 2**). This slight change in Lamin A/C staining pattern may explain why the rates of nuclear import are reduced in $PFN1^{WT}$ cells. We speculate that $PFN1^{WT}$ overexpression, by enhancing actin polymerization, may lead to a distortion of the nuclear envelope possibly via the LINC complex, thereby altering the functionality of the nuclear pore. A similar mechanism of action has been recently suggested for tau-dependent neurodegeneration in frontotemporal dementia (12,23). Future studies will be required to exactly tease out the details on how the actin cytoskeleton connection with the nucleus influences nucleocytoplasmic transport. We have included a brief discussion of these new data in the manuscript (**pg. 13**). In addition, we present in our updated manuscript that the endogenous expression of mutant PFN1 in lymphoblasts leads to changes in the localization and staining pattern of RanGAP1, Ran, Lamin A/C, and FG-Nups (**Figure 3**) suggesting are results are not merely artefacts of overexpression (see also below).

- As patient PFN1 mutant cells are available to the authors (lymphoblasts, fig 4) and have a phenotype that the authors are concluding to be related to actin/nucleocytoplasmic transport, are the other key phenotypes observed in these cells?? (membrane integrity, Ran, RanGAP, nups, import…).

We thank the Reviewer for raising such an important point, also shared by Reviewer #1. As discussed above, we have now performed an extensive analysis of nucleoporins and nucleoskeleton in lymphoblast cells derived from 3 ALS patient carrying either the C71G or G118V mutation. We found that endogenous expression of mutant PFN1 leads to changes in the localization and staining pattern of RanGAP1, Ran, Lamin A/C, and FG-Nups. We also tested for defects to the integrity of the nuclear membrane by the dextran exclusion assay, but no major difference was observed between ALS and control lines. Together, these new data strongly support our hypothesis that mutant PFN1 expression causes alteration to the nuclear pore stability possibly leading to neuronal degeneration. We have included these new data in **Figure 3** and **Supplementary Figure 6**.

2. The authors also do not comment on the negative effects of actin polymerisation enhancement on WT neurons? (6F, 7A/C/D)

We apologize for not having commented on this interesting observation. As mentioned above, we have now expanded our discussion to include this point (**pg. 13**).

3. The authors claim "these results suggest that modulation of actin homeostasis directly modifies the stability of the NPC and/or NE, protecting it from disruption caused by ALS-associated gene mutations". No data is shown to support this, only that actin modulation (mDia) can rescue RanGAP1, TDP-43 and S-mCherry distribution, no nuclear envelope defects, no nuclear pore elements.

As suggested by both Reviewer #3 and Reviewer #2, we have now further expanded our analysis of the effect of actin modulation on nucleocytoplasmic transport by employing a new complementary pharmaceutical approach to induce formin function. Primary motor

neurons expressing WT or mutant PFN1 were treated with low concentrations (i.e. 0.1µM) of the selective formin agonist Intramimic-01 (IMM01) (15) for 24h. Under these conditions, we found a significant of FG-Nups defective localization at the nuclear envelope, as well as of the Ran gradient and Neurofilament mRNA localization to the axon, significantly expanding upon our initial findings in the presence of the mDia1 overexpression. Overall, these data strongly support our hypothesis that modulation of actin polymerization directly influences nucleocytoplasmic transport. We have included these new data in **Figure 7 and Figure 9**.

4. How is actin polymerisation rescuing these effects? How is actin/cytoskeleton at the nuclear envelope during changes and rescue?

The Reviewer raises a very interesting question, namely how the actin cytoskeleton interacts with the nucleus to modulate NCT. Our data shows that genetic or pharmacologic modulation of actin polymerization directly affects the structure/function of the NPC. We believe that the dissection of the fine details of the molecular mechanism linking actin polymerization and nuclear pore stability will indeed be complex and is the subject of current investigations. As such, we feel these experiments are beyond the scope of this study. However, we do speculate within the Discussion on how changes to actin homeostasis caused by mutant PFN1 could affect the interactions between the LINC complex and the cytoskeleton, thus altering the integrity of the nuclear membrane and nuclear pores. The LINC complex physically connects the nuclear membrane to the actin cytoskeleton. Few studies have addressed the relevance of the LINC complex/actin association in post-mitotic neurons thus providing rich area of future discoveries. Interestingly, it should be noted that changes to actin polymerization through inhibition of Cofilin or increased Arp2/3 activity cause distortions and protrusions in the nuclear membrane (24–26), similarly to what we observed in mutant PFN1 cells. Furthermore, changes to microtubule polymerization caused by mutant tau have been shown to alter nuclear morphology in frontotemporal dementia models possibly through the LINC complex (12). We have now expanded our discussion to include these new evidence (**pg. 12-13**).

5. The evidence for the involvement of mRNA post-transcriptional regulation is very limited, and no evidence for disruption via modulation of actin polymerisation is shown – as stated in abstract, neither is any link made to nucleocytoplasmic transport. Expression of mutant PFN1 reduces axonal neurofilament RNA, and one splicing event is changed in lymphoblasts from PFN1 mutant ALS lines (also why are controls pooled?). Does modulation of actin polymerisation/nuclear export rescue these changes?

To address the Reviewer's concern and directly link modulation of actin polymerization/nuclear export rescue to changes in mRNA post-transcriptional regulation, we expanded on the data from our original manuscript that the improper localization of the RBP TDP-43 could be rescued by modulating actin in mutant PFN1 expressing neurons. We have now analyzed the axonal localization of the *Neurofilament* mRNA following rescue of actin polymerization in mutant PFN1 cells. To that end, we treated transfected motor neurons with the formin agonist IMM01 and performed FISH assays to measure the levels of axonal *Nefl* mRNA. We found that IMM01 treatment was able to rescue the localization defects observed in mutant PFN1 motor neurons, strongly supporting our hypothesis. We have included these new data in **Figure 7**.

6. Nuclear pore stability, structure and composition is frequently referred to, but only localisation of individual pore components is assessed.

Our data show that the localization at the NE of several essential nucleoporins such as FG-Nups and POM121 is altered, leading to functional changes to the process of nucleocytoplasmic transport. This suggests that nuclear pores are either reduced in number or structurally compromised because of the lack of essential nucleoporins. We apologize for the lack of clarity. We have amended the text accordingly (see **pg. 5**).

7. Do the authors really consider that modulating actin – one of the main cytoskeletal components in all cells in the body - is a good therapeutic strategy?? Decreasing actin polymerisation is a target for metastasis in cancer.

We agree with the Reviewer that modulating the actin cytoskeleton can have severe side effects that would render this strategy unsuitable for patient treatment. However, we argue that our data point to the nucleus/cytoskeleton interaction as a potential target for the development of novel strategies in ALS and potentially other neurodegenerative diseases. For instance, modulating the association of actin with the LINC complex could relieve excessive pressure on the nucleus thereby reverting some of the defects to the NCT in post-mitotic neurons. Indeed cytoskeletal-modulating drugs are currently under development and being tested not only in spinal cord injury but also in neurodegenerative diseases, including microtubule stabilizers and regulatory proteins such as ROCK inhibitors (27). We have revised the text to make this point clear.

Minor comments:

Some changes in clarity of description required.

- WT cells is ambiguous, these are not untransfected cells, but cells overexpression WT profilin1.

We have replaced WT with $PFN1^{WT}$ throughout the text.

- Fig 1f,G require description

We have revised the text accordingly (**pg. 5**).

- state which proteins tested, not "no change in karyopherins", not all tested, p4

We have revised the text accordingly (**pg. 5**).

- role of NCT in toxicity "strongly" argues is a bit overstated, as no toxicity is tested/rescued in this study, discussion, p16

We have revised the text accordingly (**pg. 13**).

- what viscous cycle?, discussion, p16

We are referring to a vicious (not viscous) cycle as a cascade of negative events that leads to neuronal toxicity.

No clear examples of profilin 1 forming aggregates are shown, some granular structures – is this what the authors are referring to? What % of neurons have 'aggregates' versus diffuse, and is this related to the level of transfection/expression (total cell PFN level)?

Mutant PFN1 forms small globular aggregates that are localized in the cytoplasm as shown in Figure 1. We have previously demonstrated that these aggregates are poly-ubiquitinated and partially colocalize with TDP-43 (17). The PFN1 C71G mutant is significantly more aggregation prone compared to the G118V variant, with 15.99±2.90% cells with aggregates for the C71G mutant, and 6.09±1.63% for the G118V. There is no correlation between aggregate formation and expression levels. We have included these data in **Supplementary Figure 1**.

Clarification of what is considered altered/abnormal/mislocalised should be given in the methods for each protein/structure assessed.

We have revised the text accordingly (**pg. 21**).

Table S1 – Both number of cells and number of experiments should be given

We have revised the text accordingly.

Please also explain statistical rationale for why number of experiments or cells is used as N in different experiments? Repeated measures/paired analyses can be carried out for non-parametric data using tests such as the Friedman test (if this is the reason).

We used n=experiment only when comparing %cells. For all other experiments evaluating fluorescence intensity or C:N ratio, we used n=cells.

No comment is given on frequent significant differences between mutants.

As mentioned above, the G118V mutant is less aggregation prone than the C71G variant. Since aggregates seem to worsen many of the phenotypes observed, as shown in Figure 1D, we think that this is the reason for the G118V often showing a more subtle phenotype. We have included this point in the Discussion (**pg. 11**).

Why are some experiments done in cortical neurons?

The main reason why cortical neurons were used for the import/export dynamic experiments is because motor neuron yields from individual preps were not sufficient for these assays. We would also like to note that in ALS both spinal and cortical neurons are affected, and the degeneration of cortical neurons is often associated to motor neuron loss, making cortical neurons a reasonable model to study ALS.

Bibliography

1. Taylor JP, Brown RH, Cleveland DW. Decoding ALS: from genes to mechanism. Nature. 2016 Nov 10;539(7628):197–206.

2. Cooper-Knock J, Kirby J, Ferraiuolo L, Heath PR, Rattray M, Shaw PJ. Gene expression profiling in human neurodegenerative disease. Nat Rev Neurol. 2012 Sep;8(9):518–530.

3. Kim NC, Andrews PC, Asselbergs FW, Frost HR, Williams SM, Harris BT, et al. Gene ontology analysis of pairwise genetic associations in two genome-wide studies of sporadic ALS. BioData Min. 2012 Jul 28;5(1):9.

4. Xiao S, Sanelli T, Dib S, Sheps D, Findlater J, Bilbao J, et al. RNA targets of TDP-43 identified by UV-CLIP are deregulated in ALS. Mol Cell Neurosci. 2011 Jul;47(3):167–180.

5. Egawa N, Kitaoka S, Tsukita K, Naitoh M, Takahashi K, Yamamoto T, et al. Drug screening for ALS using patient-specific induced pluripotent stem cells. Sci Transl Med. 2012 Aug 1;4(145):145ra104.

6. Chen H, Qian K, Du Z, Cao J, Petersen A, Liu H, et al. Modeling ALS with iPSCs reveals that mutant SOD1 misregulates neurofilament balance in motor neurons. Cell Stem Cell. 2014 Jun 5;14(6):796–809.

7. Fanara P, Banerjee J, Hueck RV, Harper MR, Awada M, Turner H, et al. Stabilization of hyperdynamic microtubules is neuroprotective in amyotrophic lateral sclerosis. J Biol Chem. 2007 Aug 10;282(32):23465–23472.

8. Grima JC, Daigle JG, Arbez N, Cunningham KC, Zhang K, Ochaba J, et al. Mutant huntingtin disrupts the nuclear pore complex. Neuron. 2017 Apr 5;94(1):93–107.e6.

9. Zhang K, Donnelly CJ, Haeusler AR, Grima JC, Machamer JB, Steinwald P, et al. The C9orf72 repeat expansion disrupts nucleocytoplasmic transport. Nature. 2015 Sep 3;525(7567):56–61.

10. Chou C-C, Zhang Y, Umoh ME, Vaughan SW, Lorenzini I, Liu F, et al. TDP-43 pathology disrupts nuclear pore complexes and nucleocytoplasmic transport in ALS/FTD. Nat Neurosci. 2018 Feb;21(2):228–239.

11. Eftekharzadeh B, Daigle JG, Kapinos LE, Coyne A, Schiantarelli J, Carlomagno Y, et al. Tau protein disrupts nucleocytoplasmic transport in alzheimer's disease. Neuron. 2018 Sep 5;99(5):925–940.e7.

12. Paonessa F, Evans LD, Solanki R, Larrieu D, Wray S, Hardy J, et al. Microtubules Deform the Nuclear Membrane and Disrupt Nucleocytoplasmic Transport in Tau-Mediated Frontotemporal Dementia. Cell Rep. 2019 Jan 15;26(3):582–593.e5.

13. Sivadasan R, Hornburg D, Drepper C, Frank N, Jablonka S, Hansel A, et al. C9ORF72 interaction with cofilin modulates actin dynamics in motor neurons. Nat Neurosci. 2016 Oct 10;19(12):1610–1618.

14. Freibaum BD, Lu Y, Lopez-Gonzalez R, Kim NC, Almeida S, Lee K-H, et al. GGGGCC repeat expansion in C9orf72 compromises nucleocytoplasmic transport. Nature. 2015 Sep 3;525(7567):129–133.

15. Lash LL, Wallar BJ, Turner JD, Vroegop SM, Kilkuskie RE, Kitchen-Goosen SM, et al. Small-molecule intramimics of formin autoinhibition: a new strategy to target the cytoskeletal remodeling machinery in cancer cells. Cancer Res. 2013 Nov 15;73(22):6793–6803.

16. Matsukawa K, Hashimoto T, Matsumoto T, Ihara R, Chihara T, Miura M, et al. Familial Amyotrophic Lateral Sclerosis-linked Mutations in Profilin 1 Exacerbate TDP-43-induced Degeneration in the Retina of Drosophila melanogaster through an Increase in the Cytoplasmic Localization of TDP-43. J Biol Chem. 2016 Nov 4;291(45):23464–23476.

17. Wu C-H, Fallini C, Ticozzi N, Keagle PJ, Sapp PC, Piotrowska K, et al. Mutations in the profilin 1 gene cause familial amyotrophic lateral sclerosis. Nature. 2012 Aug 23;488(7412):499–503.

18. Figley MD, Bieri G, Kolaitis R-M, Taylor JP, Gitler AD. Profilin 1 associates with stress granules and ALS-linked mutations alter stress granule dynamics. J Neurosci. 2014 Jun 11;34(24):8083–8097.

19. Tanaka Y, Nonaka T, Suzuki G, Kametani F, Hasegawa M. Gain-of-function profilin 1 mutations linked to familial amyotrophic lateral sclerosis cause seed-dependent intracellular TDP-43 aggregation. Hum Mol Genet. 2016 Apr 1;25(7):1420–1433.

20. Boopathy S, Silvas TV, Tischbein M, Jansen S, Shandilya SM, Zitzewitz JA, et al. Structural basis for mutation-induced destabilization of profilin 1 in ALS. Proc Natl Acad Sci U S A. 2015 Jun 30;112(26):7984–7989.

21. Niopek D, Wehler P, Roensch J, Eils R, Di Ventura B. Optogenetic control of nuclear protein export. Nat Commun. 2016 Feb 8;7:10624.

22. Lapalombella R, Sun Q, Williams K, Tangeman L, Jha S, Zhong Y, et al. Selective inhibitors of nuclear export show that CRM1/XPO1 is a target in chronic lymphocytic leukemia. Blood. 2012 Nov 29;120(23):4621–4634.

23. Cornelison GL, Levy SA, Jenson T, Frost B. Tau-induced nuclear envelope invagination causes a toxic accumulation of mRNA in Drosophila. Aging Cell. 2019 Feb;18(1):e12847.

24. Zallen JA, Cohen Y, Hudson AM, Cooley L, Wieschaus E, Schejter ED. SCAR is a primary regulator of Arp2/3-dependent morphological events in Drosophila. J Cell Biol. 2002 Feb 18;156(4):689–701.

25. Thiam H-R, Vargas P, Carpi N, Crespo CL, Raab M, Terriac E, et al. Perinuclear Arp2/3-driven actin polymerization enables nuclear deformation to facilitate cell migration through complex environments. Nat Commun. 2016 Mar 15;7:10997.

26. Wiggan O, Schroder B, Krapf D, Bamburg JR, DeLuca JG. Cofilin Regulates Nuclear Architecture through a Myosin-II Dependent Mechanotransduction Module. Sci Rep. 2017 Jan 19;7:40953.

27. Eira J, Silva CS, Sousa MM, Liz MA. The cytoskeleton as a novel therapeutic target for old neurodegenerative disorders. Prog Neurobiol. 2016 Apr 16;141:61–82.

Reviewers' comments:

Reviewer #1 (Remarks to the Author):

The authors have made progress towards addressing prior concerns most notably through the use of lymphoblast cell lines harboring endogenous levels of mutant PFN. However, the data still do not support their conclusions as the manuscript is currently written and many critiques have been poorly addressed. A few major concerns still remain.

Major Concerns:

The authors have now provided quantification comparing the OE of WT PFN to untransfected cells. However, this is still not the appropriate control. The authors need to compare both WT and mutant PFN OE to a transfection control. Since the PFN constructs are tagged with V5, the authors should transfect V5 alone and perform their comparisons. In later experiments using GFP tagged PFN variants, they appropriately use GFP only controls so the reviewer is unsure why they have not used this level of rigor throughout their analyses.

The authors have made an attempt to look at whether their phenotypes are an artifact of cell death and whether their pharmacological treatments lead to cell death. However, they have done so by performing caspase staining. There are multiple cell death pathways, not just apoptosis (necrosis is one example). A better measurement would be to conduct a live/dead analysis (PI incorporation, LDH uptake, Alamar blue etc). Many of the selected images appear to be of very unhealthy cells, some even include cytoplasmic DAPI staining! In addition, the authors mentioned the presence of condensed DNA in some of their analysis. This is sometimes an indicator of cell death as well. Thus, the reviewer is very unconvinced that the authors have ruled out the contribution of cell death to their observations.

The authors draw multiple conclusions regarding nuclear membrane integrity and irregularities throughout the paper based on Lamin A/C staining. However, they fail to even mention Lamin B1 which is another major component of the nuclear lamina.

Overall, many of the conclusions and interpretations of the results are not supported by the data presented. A few examples are listed below. However, this is a major flaw throughout the entire manuscript that must be addressed.
1. The authors mention multiple times that the nuclear pore is affected by PFN mutations and actin dynamics. They have very little data to support this claim. They look at 1 out of 30 nucleoporins and additionally use the 414 antibody which recognizes 4 highly mobile dynamic nups with heavy involvement in the transport process. They also look at RanGap1 which is indeed not a component of the nuclear pore, but a nuclear pore associated protein again involved in nucleocytoplasmic transport. They often mention that PFN affects the pore structure but have not conduced a comprehensive or even surface level analysis to support this claim. They even have EM images but have not looked at the structure of the pore- simply the nuclear membrane. As written, the entire manuscript is extremely misleading.
2. When describing the dextran assay the authors state “to test whether the changes in Ran localization were caused by alteration to the integrity of the nuclear membrane, we assessed the ability of a large inert molecule to bypass the NPC”. This statement is factually incorrect. Dextran assays will assess the passive permeability of the NPC itself, it has nothing to do with the nuclear membrane. Also, could the authors please describe the potential connection between Ran localization and passive diffusion changes given that Ran is heavily involved in active NCT, not passive. What is the size of Ran? If passive diffusion through the pore was affected one might expect this to be a possible explanation for the mislocalization of many proteins less than 40-60 kDa.
3. To conclude the data with lymphoblast cells the authors state “Together, these data support the hypothesis that endogenous levels of mutant PFN1 directly affects the nuclear pore structure/stability

in ALS patient cells.” The authors provide absolutely no data to support the claim of altered nuclear pore structure and stability as all of their experiments are primarily centered around a function of the NPC, NCT.
4. Additional examples of these issues can be found on lines 142 (the authors have up to this point provided no data to suggest a structural defect in the pore itself), 153/154, 228/229, 256/257 (there is no data presented regarding the stability of the pore).

The experiments conducted with endogenous levels of the C9orf72 HRE and mutant PFN were all done in dividing cells. One would expect both Nup/NPC and NCT dynamics to be very different in these cell types compared to neurons. Indeed, the authors discuss that this may be the case for some of their data in lymphoblast cells. Thus, given the limited availability of patient tissue or iPSC lines harboring PFN mutations, the authors might consider generating Crispr knock-in iPSC lines for PFN mutations to better address this concern. In addition, many C9orf72 iPSC lines are readily available and could be used instead of fibroblasts.

Minor Concerns:

The quantification of the nucleoporin and nuclear membrane abnormalities could be improved upon. Rather than % of cells that seem to be abnormal, the strength of the data and conclusions would greatly benefit from more thorough analysis (ie: nuclear volume occupied, number of folds/invaginations etc.)

The authors use fluorescence intensity to compare the levels of overexpression amongst PFN constructs. This is far from quantitative. The authors should run a western blot. Their constructs are tagged with V5 or GFP making this an even simpler request.

The authors often describe the literature inaccurately thus putting their lack of in depth knowledge on the subject matter presented on display. For example they state “interestingly, nucleoporins are the longest-lived proteins in the cell, and they are not replaces once the NPC is formed in postmitotic neurons.” This is not true. While some individual nucleoporins are extremely long-lived, namely the scaffolding structural nucleoporins, many are highly dynamic and rapidly turned, specifically those involved in directly facilitating the transport process. In addition, when describing Nups recognized by the 414 antibody, they list Nup62, Nup90, and Nup52. Only one of these (Nup62) is a mammalian Nup. Secondly, the 414 antibody recognizes Nup62, Nup153, Nup214, and Nup358 (RanBP2). A better understanding of the literature, specifically that in which they are relying on for the basis of these studies is essential.

Reviewer #2 (Remarks to the Author):

Nucleocytoplasmic transport deficits have been identified in the most common genetic cause of ALS, C9ORF72 ALS. A less common genetic cause of ALS is associated with mutations in Profilin1. Profilin1 is an actin binding protein. In this study, Giampetruzzi et al. sought to determine whether disruption in actin dynamics can contribute to nucleocytoplasmic transport impairment. A common concern for the reviewers was the overexpression of profilin1 variants. However, the authors addressed many of the concerns and brought to light some of the technical limitations associated with characterizing a mechanism in this rare genetic subtype. The authors elaborate on their work by incorporating additional genetic and pharmacological tools to establish the relationship between actin dynamics and nucleocytoplasmic transport. Furthermore, they determine that the altered transport molecules related to the cytoskeletal changes rather than apoptosis. Because nucleocytoplasmic transport deficits may play a role in neurodegenerative disease pathogenesis, this work may provide other investigators with new information on the role actin dynamics may play in this context. Given these improvements, this reviewer suggests this study is appropriate for publication in Nature Communications.

Minor concern:
The authors argue the nucleocytoplasmc transport impairment is associated with PFN1 mutations. Points 5 & 8 (referencing the absent to mild karyopherin distribution disruption) ask for further elaboration on transport mechanism. Transport by the S-mCherry reporter construct is driven by karyopherins that show little to no change in distribution. This is not well addressed in the authors responses.

Reviewer #3 (Remarks to the Author):

The authors have answered all the points raised adequately from the previous review.

RE: NCOMMS-18-28078

We thank the Reviewers for their insightful comments. Please find below a point-by-point response to the Reviewers' concerns. Changes in the manuscript are highlighted and marked by a side bar.

Reviewers' comments:

Reviewer #1 (Remarks to the Author):

The authors have made progress towards addressing prior concerns most notably through the use of lymphoblast cell lines harboring endogenous levels of mutant PFN. However, the data still do not support their conclusions as the manuscript is currently written and many critiques have been poorly addressed. A few major concerns still remain.

Major Concerns:

The authors have now provided quantification comparing the OE of WT PFN to untransfected cells. However, this is still not the appropriate control. The authors need to compare both WT and mutant PFN OE to a transfection control. Since the PFN constructs are tagged with V5, the authors should transfect V5 alone and perform their comparisons. In later experiments using GFP tagged PFN variants, they appropriately use GFP only controls so the reviewer is unsure why they have not used this level of rigor throughout their analyses.

We would first like to clarify that cells were not untransfected, but rather mock transfected meaning they went through all steps of the transfection protocol but did not received any plasmid. We apologize for not making this distinction clearer in the manuscript. It is unfortunately not technically feasible to perform the experiment as suggested by the reviewer since it is not possible to detect the expression of the V5 peptide, which is only 14 amino acids in length, by immunofluorescence (or western blot), making it impossible to detect transfected cells (**Figure 1**). Since transfected cells are very few compared with the overall neuronal population on the coverslip, cells randomly picked in the field of view of V5-empty transfected cells would most likely be untransfected – or rather mock transfected. We already provide the mock transfection control in our manuscript for all proteins analyzed (see Supplementary Fig. 2).

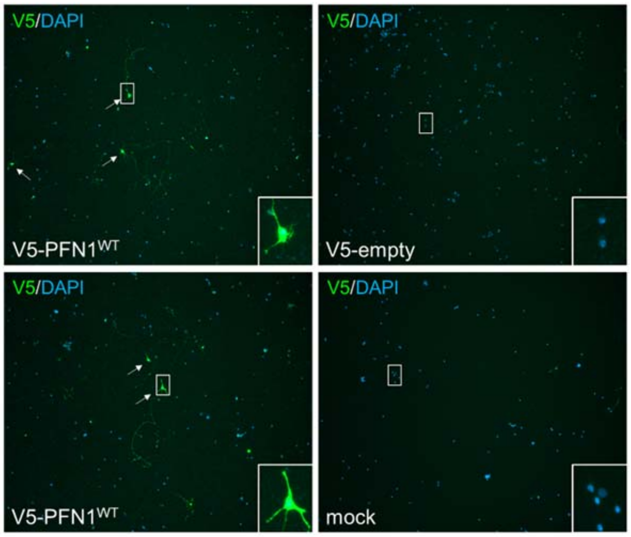

Figure 1. V5 is not detectable in transfected motor neurons. Primary motor neurons were transfected with V5-PFN1^{WT}, V5-empty vector, or no vector (mock). V5 expression was assessed by immunofluorescence using anti-V5 antibody (Novus Bio). No expression was detected in either V5-empty or mock transfected cells, in stark contrast with V5-PFN1^{WT}-expressing cells. Arrows indicate the few cells transfected in the field of view. White boxes indicate the region enlarged in the insets.

We would also like to emphasize that mock-transfections are commonly used as a control for immunofluorescence assays exactly for the reasons outlined above. In the case of GFP, we compared PFN1-expressing cells with GFP alone because (1) GFP alone can be detected allowing to visualize and select transfected cells for imaging, and (2) GFP is a large protein consisting of 238 amino acids (27 kDa) that is known to have the potential for causing cell toxicity due to increased ROS production, especially for live cell imaging experiments (Ansari et al., 2016; Liu et al., 1999). This is in stark contrast to the V5 epitope tag which is only 14 amino acids in length (1.4 kDa). No evidence of toxicity has ever been reported nor observed by the authors related to the expression of the V5 tag.

The authors have made an attempt to look at whether their phenotypes are an artifact of cell death and whether their pharmacological treatments lead to cell death. However, they have done so by performing caspase staining. There are multiple cell death pathways, not just apoptosis (necrosis is one example). A better measurement would be to conduct a live/dead analysis (PI incorporation, LDH uptake, Alamar blue etc). Many of the selected images appear to be of very unhealthy cells, some even include cytoplasmic DAPI staining!

As stated in the manuscript, we do not see increased rates of cell death by apoptosis in our MN culture due to expression of PFN1. The blue DAPI-positive staining that is present in the cytoplasm of some of the cells is not nuclear DNA, but rather staining coming from the magnetic nanobeads used for transfection. These beads are taken up by the cell by endocytosis and can linger in the cytoplasm for several days in some cells. We observe this regardless of the construct transfected. We have clarified this in the manuscript (see pg. 15). We are unsure of what parameters the Reviewer is using to state that "*Many of the selected images appear to be of very unhealthy cells*". These cells are expressing a mutant protein that is linked to MN death, causing severe changes to nuclear morphology and function, so being not perfectly healthy is to be expected. To further prove that no cell death is occurring during our experimental time frame, we have stained V5-PFN1 transfected cells with propidium iodide (PI), a membrane impermeable DNA intercalant agent that has been extensively used to detect cell death occurring by both apoptosis or necrosis. No PI staining was detected in any condition. We have included this data in Supplementary Figure 1.

In addition, the authors mentioned the presence of condensed DNA in some of their analysis. This is sometimes an indicator of cell death as well.

The presence of dense DNA in the nuclei of LatA-treated cells was pointed out by Reviewer #2 during the previous round of review. High concentrations and long incubation times with both Latrunculin and IMM01 have been shown to induce caspase-dependent cell death (Lash et al., 2013; Thomas et al., 2006). Hence, we decided to assess this potential side effect via cleaved caspase3 staining, which we deemed the most appropriate analysis for those situations. Of note, Reviewer #2 was satisfied with the data provided.

Thus, the reviewer is very unconvinced that the authors have ruled out the contribution of cell death to their observations.

We and others have proposed that NPC defects drive neuronal degeneration in ALS, rather than being the consequence of cell death. Our additional data using PI staining

further prove that no increased rates of death are observed in our cells within our experimental time frame. To further prove this point, we looked at cell doubling time for our patient-derived lymphoblast cultures. In these lines we detected several phenotypes related to the NPC, such as loss of nucleoporins at the nuclear envelope, defect in Lamin A/C staining, and loss of Ran gradient. Yet the cell growth rate and doubling time is unchanged between ALS and control lines, strongly suggesting cell death is not a prominent phenotype in these lines. We provide this data for the Reviewer, but we would be glad to add it to the manuscript if deemed necessary.

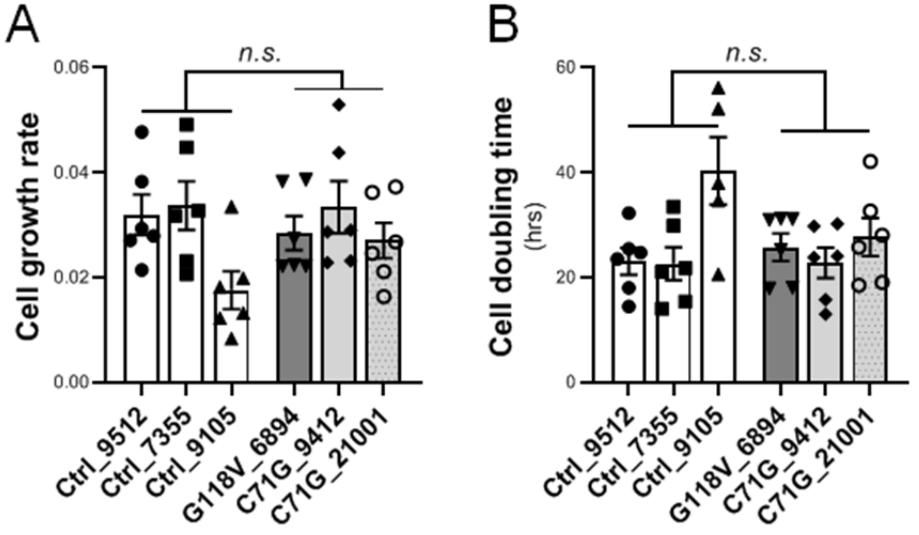

Figure 2. Quantification of cell growth rate (*A*) and doubling time (*B*) in lymphoblast lines derived from ALS patients and healthy controls. No difference was observed, suggesting no effect on cell proliferation due to the mutation. Bars are mean and SEM. One-way ANOVA, N=6.

The authors draw multiple conclusions regarding nuclear membrane integrity and irregularities throughout the paper based on Lamin A/C staining. However, they fail to even mention Lamin B1 which is another major component of the nuclear lamina.

It is true that nucleoskeleton is not composed on Lamin A/C alone, although this is one of the major components. There are many more proteins that constitute the nuclear lamina, including Lamin B1, B2, and other integral membrane proteins such as emerin, Lamin B receptor, Nurim, MAN1, LAP1A-C, and LAP2. We have added a more in-depth description of the nuclear lamina (see pg. 5), but for the purpose of our study we feel that looking at Lamin A/C is sufficient to make the point that the nuclear lamina is affected in mutant PFN1 cells. We would like to note that many other studies published in high impact journals, such as Nature and Cell, showing lamina defects in ALS models have looked at either lamin A/C or Lamin B, but not at both (Chou et al., 2018; Haase et al., 2016; Padmakumar et al., 2005; Paonessa et al., 2019; Zhang et al., 2006, 2018).

Overall, many of the conclusions and interpretations of the results are not supported by the data presented. A few examples are listed below. However, this is a major flaw throughout the entire manuscript that must be addressed.
1. The authors mention multiple times that the nuclear pore is affected by PFN mutations and actin dynamics. They have very little data to support this claim.

We respectfully disagree with the Reviewer’s criticism. Here, we clearly show that both genetic (i.e. mutant PFN1, mDia1 expression) and pharmacological (i.e. Latrunculin A and IMM01) modulation of actin has consequences on the localization to the nuclear envelope of nucleoporins and associated proteins (i.e. mAb414, RanGAP1), and on the nuclear import (import dynamics, localization of RBPs). We feel these data together strongly support our claim. Furthermore, Reviewers #2 and #3 have directly stated their support of our conclusions and publication of our study.

They look at 1 out of 30 nucleoporins and additionally use the 414 antibody which recognizes 4 highly mobile dynamic nups with heavy involvement in the transport process. They also look at RanGap1 which is indeed not a component of the nuclear pore, but a nuclear pore associated protein again involved in nucleocytoplasmic transport.

We respectfully disagree with the Reviewer’s assessment. Within our study, we utilized the mAb414 antibody, a well-established marker of nuclear pores used in countless studies published in high impact journals that detects 4 different Nups located in different regions of the NPC (e.g. (Eftekharzadeh et al., 2018; Eibauer et al., 2015; Gigante et al., 2017; Schachtrup et al., 2015). Although several of these studies strictly used mAb414 in their studies, we extended our characterization to include POM121 and RanBP2/Nup358. Thus, our analysis included a total of 5 Nups, not 1 as the Reviewer stated. We maintain this is a sufficient number of nuclear pore-associated proteins to support our conclusions. Furthermore, we made no claim in our manuscript that RanGAP1 is a component of the pore, but clearly state that is associated to it.

They often mention that PFN affects the pore structure but have not conduced a comprehensive or even surface level analysis to support this claim. They even have EM images but have not looked at the structure of the pore- simply the nuclear membrane. As written, the entire manuscript is extremely misleading.

The reviewer is correct that we do not have EM data to show structural defects to NPCs. We infer that the NPC structure or numbers must be defective based on the mislocalization and lack of staining at the nuclear envelope of the Nups tested. We specify this in the manuscript clearly:

“In all, these data suggest that in the presence of mutant PFN1 NPCs are either reduced in number or structurally compromised because of the lack of essential nucleoporins, and additional key players in NCT are abnormally distributed.”

It is our opinion that performing an in-depth EM analysis of NPC structure is well-beyond the scope of this manuscript. Furthermore, we again would like to point out that no published study evaluating NCT defects in ALS models have performed such an extensive EM analysis on NPC structure. Again, these include studies in high impact journals including Cell, Nature Neuroscience and Nature (Chou et al., 2018; Eftekharzadeh et al., 2018; Freibaum et al., 2015; Grima et al., 2017; Zhang et al., 2015, 2018).

2. When describing the dextran assay the authors state “to test whether the changes in Ran localization were caused by alteration to the integrity of the nuclear membrane, we assessed the ability of a large inert molecule to bypass the NPC”. This statement is factually incorrect.

Dextran assays will assess the passive permeability of the NPC itself, it has nothing to do with the nuclear membrane.

The Reviewer may not be considering that two different dextrans are used in this assay. A 70KDa dextran that can leak in the nucleus only if the NPC permeability is compromised, and a larger 500KDa dextran that can enter the nucleus only if the membrane is compromised.

Previous publications have described the Dextran Assay as a measure of nuclear membrane integrity, intended as a combination of nuclear membrane and NPC permeability. Such descriptions include:

"Potential defects in nuclear membrane integrity and the permeability barrier of NPCs can be directly tested by using fluorescent dextrans with defined molecular weights because molecules larger than 60 kDa are excluded from intact nuclei" (D'Angelo et al., 2009)

The paper cited by D'Angelo et a. defines the dextran assay as a measurement of the breakdown of the nuclear envelope (Lénárt et al., 2003)

"*Fluorescence intensity in the nucleus is a measure of nuclear penetration of 70 kDa dextran and thus an indicator of passive permeability of the nuclear envelope*." (Strasser et al., 2012)

"*We therefore set out to evaluate whether loss of* O-*GlcNAc and decreased incorporation of Nups would compromise the NPC selectivity barrier using inert fluorescent dextrans of different molecular weights in a dextran exclusion assay (Lenart and Ellenberg, 2006). This assay enables the integrity of nuclei to be studied, and is reportedly more sensitive than active transportation assays for evaluating NPC function (Lenart et al., 2003)"(Zhu et al., 2016)*

Also, could the authors please describe the potential connection between Ran localization and passive diffusion changes given that Ran is heavily involved in active NCT, not passive. What is the size of Ran? If passive diffusion through the pore was affected one might expect this to be a possible explanation for the mislocalization of many proteins less than 40-60 kDa.

Changes to Ran localization are a typical phenotype associated with defects to the NPC. Ran is 25 KDa and can freely diffuse through the nuclear pore, yet in normal cells it is highly enriched in the nucleus thanks to an efficient nuclear import system. Ran mislocalization to the cytoplasm could thus be caused by diffusion through a dysfunctional pore or defective nuclear import. Our data suggest the latter, although we cannot fully exclude alternative explanations.

3. To conclude the data with lymphoblast cells the authors state "Together, these data support the hypothesis that endogenous levels of mutant PFN1 directly affects the nuclear pore structure/stability in ALS patient cells." The authors provide absolutely no data to support the claim of altered nuclear pore structure and stability as all of their experiments are primarily centered around a function of the NPC, NCT.

And similarly,

4. Additional examples of these issues can be found on lines 142 (the authors have up to this point provided no data to suggest a structural defect in the pore itself), 153/154, 228/229, 256/257 (there is no data presented regarding the stability of the pore).

In our revised manuscript we show that mAb414 and RanGAP1 staining at the nuclear envelope is altered, Lamin A/C staining is disrupted, and that Ran gradient is dissolved in 3 independent patient derived lymphoblast lines. Thus, we conclude that NPC structure/stability is compromised. With the caveat that we have not performed an extensive EM analysis of the nuclear pore (see above), we strongly believe our conclusions are fully supported by our experimental data. Although not the only function, NCT is the major function performed by the NPC so linking the two together is a logical step.

The experiments conducted with endogenous levels of the C9orf72 HRE and mutant PFN were all done in dividing cells. One would expect both Nup/NPC and NCT dynamics to be very different in these cell types compared to neurons. Indeed, the authors discuss that this may be the case for some of their data in lymphoblast cells. Thus, given the limited availability of patient tissue or iPSC lines harboring PFN mutations, the authors might consider generating Crispr knock-in iPSC lines for PFN mutations to better address this concern. In addition, many C9orf72 iPSC lines are readily available and could be used instead of fibroblasts.

We agree this is an important future direction but we maintain creating these cell lines for the current manuscript is far beyond the scope of this study. Looking at patient derived lymphoblasts or fibroblasts, although actively dividing cells, is a well-accepted method, as for instance shown in Chou et al., 2018. It should be noted that because lymphoblasts and fibroblasts refresh their pool of NPCs at every cell division, the effect of mutant ALS proteins would be more subtle in these cell types than in post-mitotic neurons, where defect can accumulate over time. Thus, our data may be underestimating the effect of mutant PFN1 on the NPC in patient derived cells.

Minor Concerns:

The quantification of the nucleoporin and nuclear membrane abnormalities could be improved upon. Rather than % of cells that seem to be abnormal, the strength of the data and conclusions would greatly benefit from more thorough analysis (ie: nuclear volume occupied, number of folds/invaginations etc.)

It is our opinion that the analysis performed was the most accurate method to evaluate NPC defects in primary MNs. Since MN cell bodies are very large, nuclei tend to have complex morphologies with several folds even in untransfected normal cells.

The authors use fluorescence intensity to compare the levels of overexpression amongst PFN constructs. This is far from quantitative. The authors should run a western blot. Their constructs are tagged with V5 or GFP making this an even simpler request.

We cannot perform WB on MNs cultures as yields are too low and transfection efficiency is less than 30% in this cell type. We strongly believe immunofluorescence is as quantitative as WBs, as it has been shown in study after study, with the advantage of being able to look at individual cells rather than a mix of all (transfected and untransfected) cells.

The authors often describe the literature inaccurately thus putting their lack of in depth knowledge on the subject matter presented on display. For example they state "interestingly, nucleoporins are the longest-lived proteins in the cell, and they are not replaces once the NPC is formed in postmitotic neurons." This is not true. While some individual nucleoporins are extremely long-lived, namely the scaffolding structural nucleoporins, many are highly dynamic and rapidly turned, specifically those involved in directly facilitating the transport process.

We do not disagree with the Reviewer that we are not experts of the NPC. Rather, our expertise is in the area of ALS. However, this should not preclude us from performing a study of the NPC in ALS. It is our belief that such studies spanning multiple disciplines are of high significance. This is again illustrated by previous studies carried by ALS experts describing NPC defects in ALS in high impact journals. However, we should clarify that our statements were not to be interpreted as **all** nucleoporins are never replaced in post-mitotic neurons. We apologize for the lack of clarity and have rephrased the sentence in question to be more precise. We are well aware that many Nups are highly dynamic and move in and out of the NPC to regulate gene expression, chromatin structure, and more. However, it has been reported that synthesis of new Nups sharply decreases in post-mitotic cells and that aging causes a decrease in NPC turnover (Bano et al., 2010; D'Angelo et al., 2009; Hetzer, 2010; Savas et al., 2012). For instance, Savas et al (2012) published a very elegant study in Science where they performed a comprehensive Mass Spec analysis of long-lived proteins in rat brains fed with heavy isotopes for 6 weeks after birth. The authors found that heavy isotope-labelled NPC components were still present in the brains of 12-month-old animals. Thus, they concluded:

"Thus, unlike other large protein complexes in which all components have similar turnover values (1, 2), the individual components of NPCs have very different lifetimes. This supports the idea that NPCs are built to last the entire life span of the cell and are not completely removed and assembled anew in postmitotic cells. Rather, NPC maintenance in nondividing cells relies on the non- or extremely slow exchange of scaffold and rapid replacement of peripheral Nups."

In addition, when describing Nups recognized by the 414 antibody, they list Nup62, Nup90, and Nup52. Only one of these (Nup62) is a mammalian Nup. Secondly, the 414 antibody recognizes Nup62, Nup153, Nup214, and Nup358 (RanBP2). A better understanding of the literature, specifically that in which they are relying on for the basis of these studies is essential.

We thank the Reviewer for pointing out this oversight. Indeed, it was listed as such on the manufacturer's product sheet as shown.

Epitope

ab24609 recognizes the conserved domain FXFG repeats in nucleaporins like the p62, p152, p90.

https://www.abcam.com/nuclear-pore-complex-proteins-antibody-mab414-chip-grade-ab24609.html

We have amended that section to reference the correct Nups as described in (Cronshaw et al., 2002).

Reviewer #2 (Remarks to the Author):

Nucleocytoplasmic transport deficits have been identified in the most common genetic cause of ALS, C9ORF72 ALS. A less common genetic cause of ALS is associated with mutations in Profilin1. Profilin1 is an actin binding protein. In this study, Giampetruzzi et al. sought to determine whether disruption in actin dynamics can contribute to nucleocytoplasmic transport impairment. A common concern for the reviewers was the overexpression of profilin1 variants. However, the authors addressed many of the concerns and brought to light some of the technical limitations associated with characterizing a mechanism in this rare genetic subtype. The authors elaborate on their work by incorporating additional genetic and pharmacological tools to establish the relationship between actin dynamics and nucleocytoplasmic transport. Furthermore, they determine that the altered transport molecules related to the cytoskeletal changes rather than apoptosis. Because nucleocytoplasmic transport deficits may play a role in neurodegenerative disease pathogenesis, this work may provide other investigators with new information on the role actin dynamics may play in this context. Given these improvements, this reviewer suggests this study is appropriate for publication in Nature Communications.

> We would like to thank Reviewer #2 for their very supportive comments for our manuscript and their statement that the "study is appropriate for publication in Nature Communications."

Minor concern:
The authors argue the nucleocytoplasmc transport impairment is associated with PFN1 mutations. Points 5 & 8 (referencing the absent to mild karyopherin distribution disruption) ask for further elaboration on transport mechanism. Transport by the S-mCherry reporter construct is driven by karyopherins that show little to no change in distribution. This is not well addressed in the authors responses.

> We agree with the Reviewer that this an important finding worthy of additional investigation. Indeed, we plan to continue our studies into PFN1-dependent disruption of NCT, including an in-depth analysis of the distribution and dynamics of additional karyopherins.

Reviewer #3 (Remarks to the Author):

The authors have answered all the points raised adequately from the previous review.

> We would like to thank Reviewer #3 for their supportive comment for our manuscript and for their view that we have addressed all previous concerns.

Bibliography

Ansari, A.M., Ahmed, A.K., Matsangos, A.E., Lay, F., Born, L.J., Marti, G., Harmon, J.W., and Sun, Z. (2016). Cellular GFP toxicity and immunogenicity: potential confounders in in vivo cell tracking experiments. Stem Cell Rev *12*, 553–559.

Bano, D., Hengartner, M.O., and Nicotera, P. (2010). Nuclear pore complex during neuronal degeneration: cracking the last barrier! Nucleus *1*, 136–138.

Chou, C.C., Zhang, Y., Umoh, M.E., Vaughan, S.W., Lorenzini, I., Liu, F., Sayegh, M., Donlin-Asp, P.G., Chen, Y.H., Duong, D.M., et al. (2018). TDP-43 pathology disrupts nuclear pore complexes and nucleocytoplasmic transport in ALS/FTD. Nat Neurosci *21*, 228–239.

Cronshaw, J.M., Krutchinsky, A.N., Zhang, W., Chait, B.T., and Matunis, M.J. (2002). Proteomic analysis of the mammalian nuclear pore complex. J Cell Biol *158*, 915–927.

D'Angelo, M.A., Raices, M., Panowski, S.H., and Hetzer, M.W. (2009). Age-dependent deterioration of nuclear pore complexes causes a loss of nuclear integrity in postmitotic cells. Cell *136*, 284–295.

Eftekharzadeh, B., Daigle, J.G., Kapinos, L.E., Coyne, A., Schiantarelli, J., Carlomagno, Y., Cook, C., Miller, S.J., Dujardin, S., Amaral, A.S., et al. (2018). Tau protein disrupts nucleocytoplasmic transport in alzheimer's disease. Neuron *99*, 925–940.e7.

Eibauer, M., Pellanda, M., Turgay, Y., Dubrovsky, A., Wild, A., and Medalia, O. (2015). Structure and gating of the nuclear pore complex. Nat Commun *6*, 7532.

Freibaum, B.D., Lu, Y., Lopez-Gonzalez, R., Kim, N.C., Almeida, S., Lee, K.H., Badders, N., Valentine, M., Miller, B.L., Wong, P.C., et al. (2015). GGGGCC repeat expansion in C9orf72 compromises nucleocytoplasmic transport. Nature *525*, 129–133.

Gigante, C.M., Dibattista, M., Dong, F.N., Zheng, X., Yue, S., Young, S.G., Reisert, J., Zheng, Y., and Zhao, H. (2017). Lamin B1 is required for mature neuron-specific gene expression during olfactory sensory neuron differentiation. Nat Commun *8*, 15098.

Grima, J.C., Daigle, J.G., Arbez, N., Cunningham, K.C., Zhang, K., Ochaba, J., Geater, C., Morozko, E., Stocksdale, J., Glatzer, J.C., et al. (2017). Mutant huntingtin disrupts the nuclear pore complex. Neuron *94*, 93–107.e6.

Haase, K., Macadangdang, J.K., Edrington, C.H., Cuerrier, C.M., Hadjiantoniou, S., Harden, J.L., Skerjanc, I.S., and Pelling, A.E. (2016). Extracellular forces cause the nucleus to deform in a highly controlled anisotropic manner. Sci Rep *6*, 21300.

Hetzer, M.W. (2010). The role of the nuclear pore complex in aging of post-mitotic cells. Aging *2*, 74–75.

Lash, L.L., Wallar, B.J., Turner, J.D., Vroegop, S.M., Kilkuskie, R.E., Kitchen-Goosen, S.M., Xu, H.E., and Alberts, A.S. (2013). Small-molecule intramimics of formin autoinhibition: a new strategy to target the cytoskeletal remodeling machinery in cancer cells. Cancer Res *73*, 6793–6803.

Lénárt, P., Rabut, G., Daigle, N., Hand, A.R., Terasaki, M., and Ellenberg, J. (2003). Nuclear envelope breakdown in starfish oocytes proceeds by partial NPC disassembly followed by a rapidly spreading fenestration of nuclear membranes. J Cell Biol *160*, 1055–1068.

Liu, H.S., Jan, M.S., Chou, C.K., Chen, P.H., and Ke, N.J. (1999). Is green fluorescent protein toxic to the living cells? Biochem Biophys Res Commun *260*, 712–717.

Padmakumar, V.C., Libotte, T., Lu, W., Zaim, H., Abraham, S., Noegel, A.A., Gotzmann, J., Foisner, R., and Karakesisoglou, I. (2005). The inner nuclear membrane protein Sun1 mediates the anchorage of Nesprin-2 to the nuclear envelope. J Cell Sci *118*, 3419–3430.

Paonessa, F., Evans, L.D., Solanki, R., Larrieu, D., Wray, S., Hardy, J., Jackson, S.P., and Livesey, F.J. (2019). Microtubules Deform the Nuclear Membrane and Disrupt Nucleocytoplasmic Transport in Tau-Mediated Frontotemporal Dementia. Cell Rep *26*, 582–593.e5.

Savas, J.N., Toyama, B.H., Xu, T., Yates, J.R., and Hetzer, M.W. (2012). Extremely long-lived nuclear pore proteins in the rat brain. Science *335*, 942.

Schachtrup, C., Ryu, J.K., Mammadzada, K., Khan, A.S., Carlton, P.M., Perez, A., Christian, F., Le Moan, N., Vagena, E., Baeza-Raja, B., et al. (2015). Nuclear pore complex remodeling by p75(NTR) cleavage controls TGF-β signaling and astrocyte functions. Nat Neurosci *18*, 1077–1080.

Strasser, C., Grote, P., Schäuble, K., Ganz, M., and Ferrando-May, E. (2012). Regulation of nuclear envelope permeability in cell death and survival. Nucleus *3*, 540–551.

Thomas, S.G., Huang, S., Li, S., Staiger, C.J., and Franklin-Tong, V.E. (2006). Actin depolymerization is sufficient to induce programmed cell death in self-incompatible pollen. J Cell Biol *174*, 221–229.

Zhang, J., Ito, H., Wate, R., Ohnishi, S., Nakano, S., and Kusaka, H. (2006). Altered distributions of nucleocytoplasmic transport-related proteins in the spinal cord of a mouse model of amyotrophic lateral sclerosis. Acta Neuropathol *112*, 673–680.

Zhang, K., Donnelly, C.J., Haeusler, A.R., Grima, J.C., Machamer, J.B., Steinwald, P., Daley, E.L., Miller, S.J., Cunningham, K.M., Vidensky, S., et al. (2015). The C9orf72 repeat expansion disrupts nucleocytoplasmic transport. Nature *525*, 56–61.

Zhang, K., Daigle, J.G., Cunningham, K.M., Coyne, A.N., Ruan, K., Grima, J.C., Bowen, K.E., Wadhwa, H., Yang, P., Rigo, F., et al. (2018). Stress granule assembly disrupts nucleocytoplasmic transport. Cell *173*, 958–971.e17.

Zhu, Y., Liu, T.W., Madden, Z., Yuzwa, S.A., Murray, K., Cecioni, S., Zachara, N., and Vocadlo, D.J. (2016). Post-translational O-GlcNAcylation is essential for nuclear pore integrity and maintenance of the pore selectivity filter. J Mol Cell Biol *8*, 2–16.

REVIEWERS' COMMENTS:

Reviewer #1 (Remarks to the Author):

The authors have provided much needed clarification and explanation of their data in the revised manuscript. The true novelty and contribution of this manuscript is the effects of PFN1 mutations on the nuclear lamina. The nuclear lamina is known to be stabilized by actin and microtubule filaments and actin dynamics have previously been linked to nuclear membrane stability (reviewed in Houben et al 2007). Thus, the authors provide data that suggests PFN1 mediated disruptions of actin dynamics may contribute to altered nuclear membrane integrity in ALS.

While most concerns have been addressed, it is still disappointing that the authors fail to conduct further analyses regarding nuclear pore structure on their already obtained EM images. Recent reports (Saberi et al. 2018, Acta Neuropathologica, Vatsavayal et al. 2019, Acta Neuropathologica) have suggested that alterations in the structure of the nuclear lamina (which the authors define here for PFN1 variants) sometimes lead to misinterpretation of alterations in the nuclear distribution of nucleoporins (also observed for "FG Nups" and POM121 but the authors). The existence of pores along nuclear invaginations ultimately affects distribution but may not necessarily fully affect pore integrity. Thus, if this is the case, much of the data presented here regarding NPCs, takes on a slightly different interpretation. It is disappointing that this possibility is not addressed or discussed especially given that the EM data presented could begin to shed light on this.

RE: NCOMMS-18-28078

Please find below a point-by-point response to the Reviewer' concerns and Editorial requests. Changes in the manuscript are marked by the "Track Changes" feature.

Reviewer #1 (Remarks to the Author):

The authors have provided much needed clarification and explanation of their data in the revised manuscript. The true novelty and contribution of this manuscript is the effects of PFN1 mutations on the nuclear lamina. The nuclear lamina is known to be stabilized by actin and microtubule filaments and actin dynamics have previously been linked to nuclear membrane stability (reviewed in Houben et al 2007). Thus, the authors provide data that suggests PFN1 mediated disruptions of actin dynamics may contribute to altered nuclear membrane integrity in ALS.

We thank the Reviewer for acknowledging our efforts and the novelty of our study.

While most concerns have been addressed, it is still disappointing that the authors fail to conduct further analyses regarding nuclear pore structure on their already obtained EM images. Recent reports (Saberi et al. 2018, Acta Neuropathologica, Vatsavayal et al. 2019, Acta Neuropathologica) have suggested that alterations in the structure of the nuclear lamina (which the authors define here for PFN1 variants) sometimes lead to misinterpretation of alterations in the nuclear distribution of nucleoporins (also observed for "FG Nups" and POM121 but the authors). The existence of pores along nuclear invaginations ultimately affects distribution but may not necessarily fully affect pore integrity. Thus, if this is the case, much of the data presented here regarding NPCs, takes on a slightly different interpretation. It is disappointing that this possibility is not addressed or discussed especially given that the EM data presented could begin to shed light on this.

We thank the Reviewer for raising this very important point. The EM images were obtained with the purpose of analyzing the nuclear membrane structure and were thus individual focal plan rather than 3D stacks. This prevented us from using them to investigate the structure of the nuclear pore as suggested by the Reviewer. However, we appreciate the concern of the Reviewer about misinterpretation of folded nuclei as diffuse staining after 3D images are compressed via max projections to 2D images, as described in Saberi et al., and Vatsavayal et al. We would like to point out that the analyses of nucleoporin localization and staining pattern were all performed on 3D stacks of individual optical slices, that were not compressed to 2D images. Thus, we were able to discriminate between loss of staining at the nuclear membrane, diffuse cytoplasmic/nuclear staining, and folded and invaginated membrane. We have included a more thorough description of the analysis protocol in the Methods section.
